# Molecular Recognition of Tyrosine-Containing Polypeptides with Pseudopeptidic Cages Unraveled by Fluorescence and NMR Spectroscopies

**DOI:** 10.1021/acs.bioconjchem.3c00455

**Published:** 2023-12-11

**Authors:** Naiara Solozabal, Lucía Tapia, Jordi Solà, Yolanda Pérez, Ignacio Alfonso

**Affiliations:** † NMR Facility, Institute for Advanced Chemistry of Catalonia, IQAC-CSIC, Jordi Girona 18-26, Barcelona 08034, Spain; ‡ Department of Biological Chemistry, Institute for Advanced Chemistry of Catalonia, IQAC-CSIC, Jordi Girona 18-26, Barcelona 08034, Spain

## Abstract

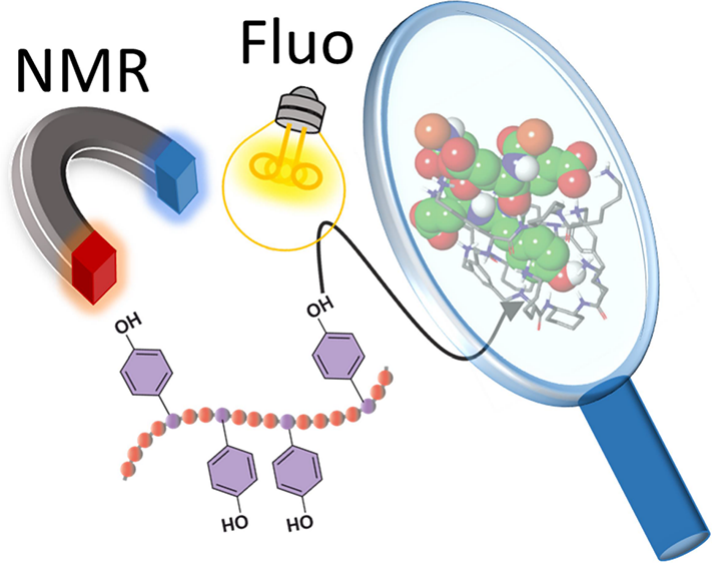

The molecular recognition of Tyr-containing peptide copolymers with pseudopeptidic cages has been studied using a combination of fluorescence and NMR spectroscopies. Fluorescence titrations rendered a reasonable estimation of the affinities, despite the presence of dynamic quenching masking the unambiguous detection of the supramolecular complexes. Regarding NMR, the effect of polypeptide (PP) binding on relaxation and diffusion parameters of the cages is much more reliable than the corresponding chemical shift perturbations. To that, purification of the commercial PPs is mandatory to obtain biopolymers with lower polydispersity. Thus, the relaxation/diffusion-filtered ^1^H spectra of the cages in the absence vs presence of the PPs represent a suitable setup for the fast detection of the noncovalent interactions. Additional key intermolecular NOE cross-peaks supported by molecular models allow the proposal of a structure of the supramolecular species, stabilized by the Tyr encapsulation within the cage cavity and additional attractive polar interactions between the side chains of cage and PP, thus defining a binding epitope with a potential for implementing sequence selectivity. Accordingly, the cages bearing positive/negative residues prefer to bind the peptides having complementary negative/positive side chains close to the target Tyr, suggesting an electrostatic contribution to the interaction. Overall, our results show that both techniques represent a powerful and complementary combination for studying cage-to-PP molecular recognition processes.

## 1 Introduction

**Figure 1:**
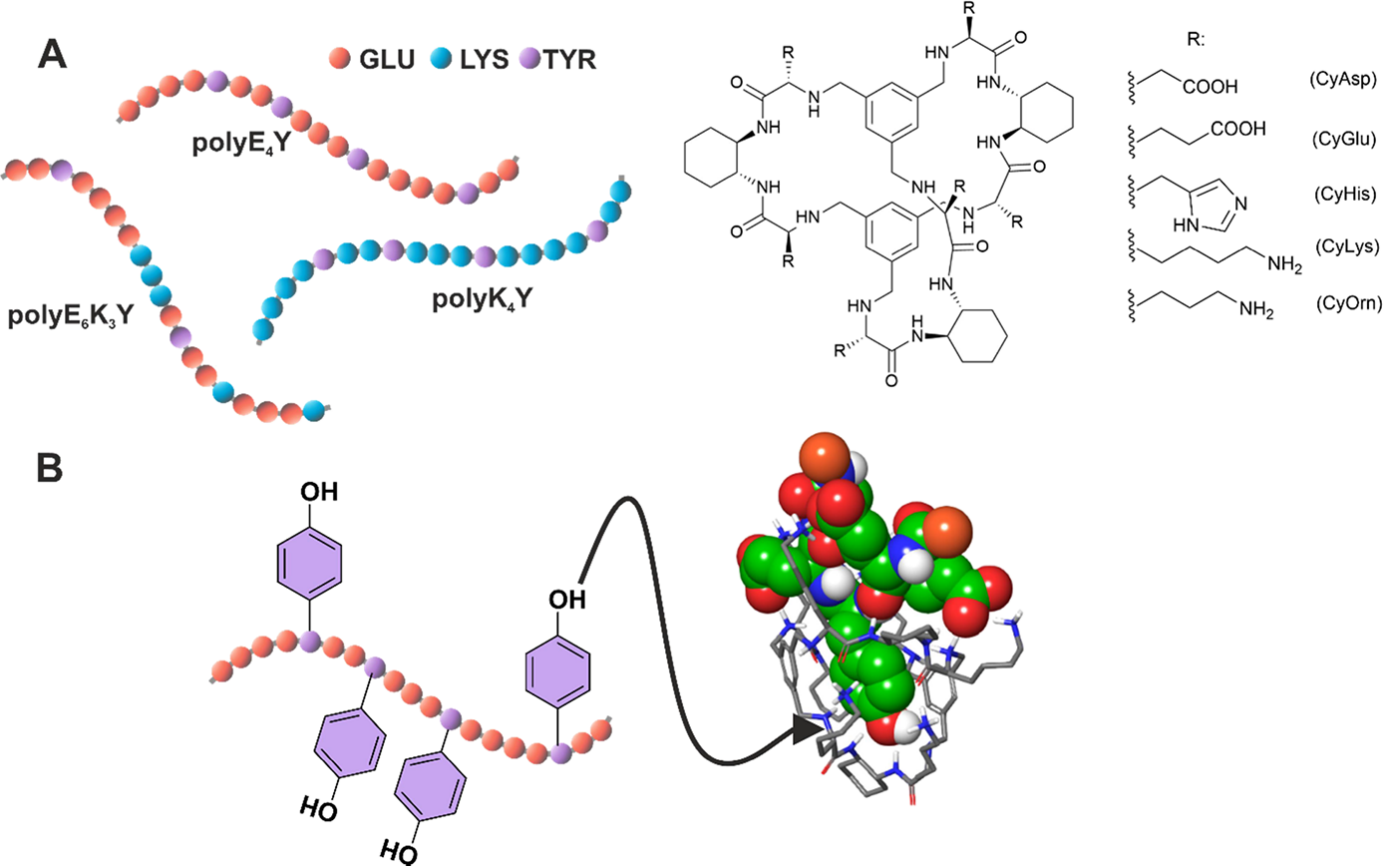
(A) Schematic representation of tyrosine random copolymers and molecular structures of the cages investigated in this work. (B) Proposed structure (Macro Model) for the [CyLys·Ac-EEYEE-NH_2_] supramolecular complex (nonpolar H atoms omitted and peptide substrate in green CPK).

The molecular recognition of protein–protein interaction (PPI) surfaces for the modulation of protein function with synthetic molecules is a hot topic in supramolecular chemistry and chemical biology.^1−5^ However, the design of efficient receptors for protein surfaces encounters several challenges.^6,7^ On the one hand, PPI surfaces are typically solvent-exposed and highly solvated, forcing the binders to overcome hydration energies. Moreover, they are relatively shallow binding sites, leading to flat and flexible ligands with concomitant entropic cost upon complexation. As a special case of PPI sites, Tyr residues and the consensus sequences that flank them are relevant biological targets, since Tyr phosphorylation by protein tyrosine kinases (PTKs) is one the most prevalent posttranslational modifications in signal transduction and cell regulation.^8^ These specific PPI sites are usually located in disordered regions of the regulated proteins, complicating the rational design of receptors due to the lack of reliable structural information on many of these epitopes.^9,10^ However, Tyr recognition in proteins is appealing as a PTK-modulation mechanism alternative (or complementary) to targeting catalytic or ATP-binding sites, which is the most common one for PTK inhibitors.^11−13^ The ATP-binding site in PTKs is highly conserved, which makes their putative ligands suffer from modest selectivity.^14^ Regarding that, in previous work, our group designed pseudopeptidic cages as efficient receptors for short peptides in organic or aqueous–organic solvents, showing a good selectivity for the Ac-EYE-NH_2_ sequence.^15,16^ More recently, we have reported a thorough structural analysis of the supramolecular complexes between these cages and the minimal Ac-EYE-NH_2_ binding epitope, both in buffered aqueous solution and in the gas phase.^17^ Additionally, we have shown that pseudopeptidic cages efficiently protect substrates from the action of the c-Src PTK, precluding the corresponding Tyr phosphorylation by selective Tyr encapsulation. In some of these studies and in line with previous reports,^18^ we used a synthetic random copolymer (polyE_4_Y) as a protein proxy. These polymeric substrates are commercially available, have a high concentration of binding sites, and show lower Michaelis constants (*K*_m_) as PTK substrates than shorter peptide consensus sequences.^19^ Despite these promising results, for a more suitable design of improved cages as Tyr receptors in proteins, a deeper knowledge of the binding phenomena is required. However, the recognition of Tyr side chains within a macromolecular structure and embedded in different chemical environments is an underexplored topic. Accordingly, we decided to tackle the binding abilities of the pseudopeptidic cages against different polypeptides (PPs) bearing Tyr surrounded by differently charged amino acids (Figure 1). The polymeric nature of the peptide substrates makes this task especially challenging due to the very peculiar properties of the corresponding supramolecular complexes, requiring a particular combination of experimental techniques. On the one hand, the Tyr residue fluorescence emission seems an obvious and convenient option since it could render a good estimation of the association constants and a molecular picture of the Tyr environment upon cage binding.^20^ The high sensitivity of the technique will allow for minimal sample consumption and relatively easy performance. As a drawback, the molecular interpretation of the results is not always trivial. As a complementary technique, NMR is extremely powerful in obtaining additional structural and dynamic information on the supramolecular complexes.^21^ Thus, NMR spectroscopy is specifically suitable to study moderate host–guest interactions (*K*_D_ > 1 × 10^–4^ M). Moreover, NMR allows to detect which protons/regions of the cages and PPs are in contact (the so-called binding region). For NMR ligand-based methods used in this context, the biopolymer must have a much larger size than the small molecule. From that point of view, our cage–Tyr-PP supramolecular complexes are unconventional systems, as they are composed of a medium-size receptor (host and cage) and a large multivalent ligand (guest and PP). In addition, the degree of conformational flexibility of those medium-sized pseudopeptides is relatively high. Therefore, we aimed to evaluate the scope and limitations of different experimental techniques for studying the corresponding cages Tyr-PP supramolecular complexes in buffered water. The long-term objective of this optimization is to set up a fast-screening protocol (either by fluorescence, NMR, or a combination of both) to evaluate protein recognition of pseudopeptidic cages targeting PPI sites.

## 2 Results and Discussion

### 2.1 Cage-PP Binding Studied by Fluorescence Emission

**Figure 2:**
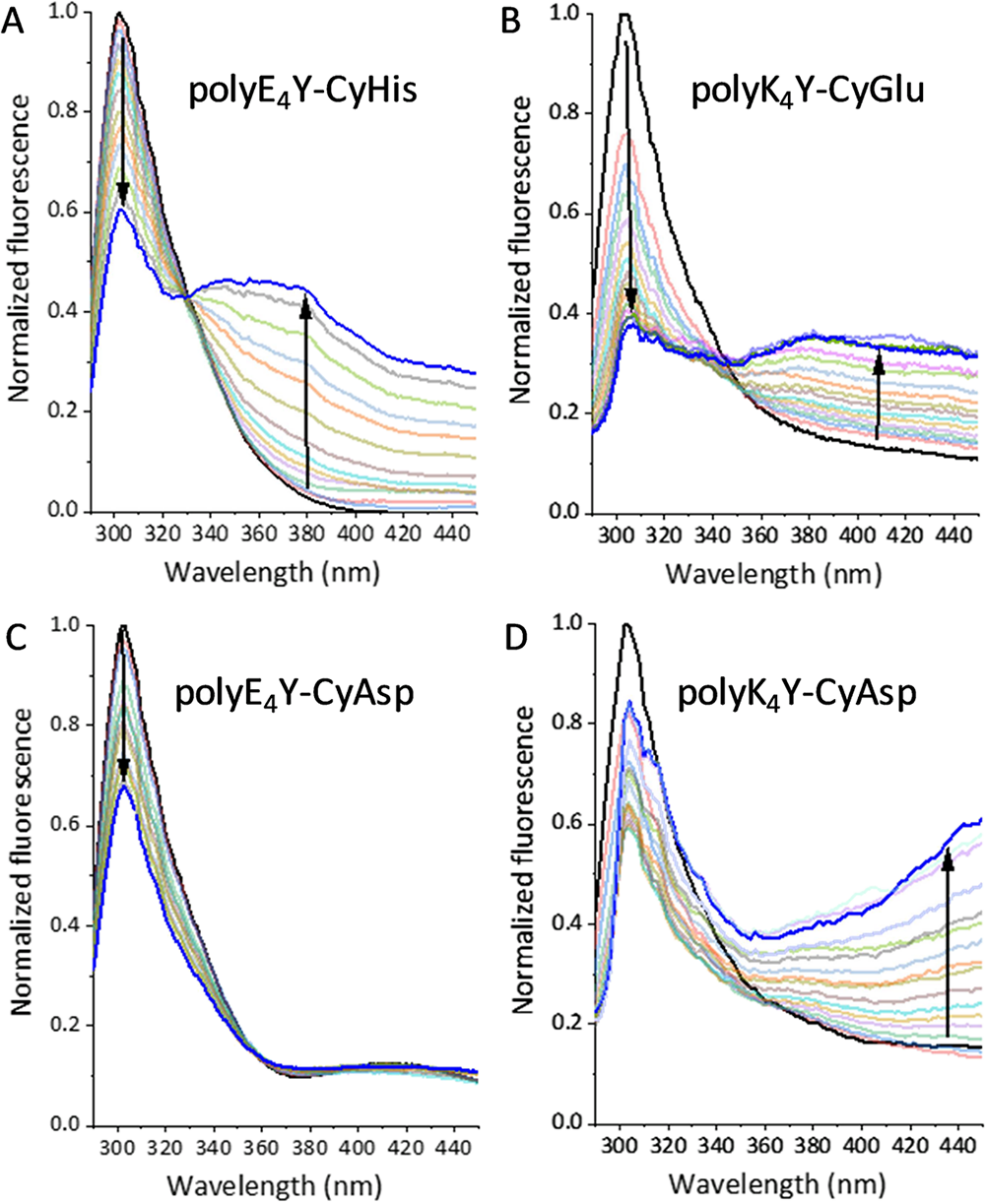
(A–D) Normalized fluorescence emission spectra (λ_exc_ = 276 nm) of a solution of the PPs (2 × 10^–5^ or 2 × 10^–4^ M in the corresponding repeating units, 50 mM Tris-HCl buffer, and 293 K) upon addition of increasing amount of a pseudopeptidic cage. The specific PP and cage are depicted in each panel.

The molecular recognition of PPs by pseudopeptidic cages comprises the inclusion of Tyr residue within the cage cavity,^22,23^ which strongly affects the fluorescence emission spectrum of the peptides upon excitation at 276 nm.^17,24^ These spectral changes can be used to detect and characterize the binding. The titration of aqueous solutions (Tris-HCl buffer) of the PPs with increasing amounts of the cages led to different spectral variations depending on both PP and cage nature (Figure 2). In most cases (Figure 2A,B), the interaction produced a decrease in the Tyr emission at 302 nm with the concomitant appearance of a lower energy emission broadband suggesting the formation of a different species in solution, ascribed to the Tyr-cage inclusion complex.^25^ In this regard, red-shifted emission of Tyr fluorophore has been related to the formation of stable tyrosinate anion in the excited state as a consequence of strongly H-bound Tyr complexes in the ground state.^26^ In these cases, the growing band was globally fitted to a 1:1 binding model with respect to the Tyr residues present in the samples (Table 1). This approximation assumes all the Tyr residues are equivalent regardless of their position in the PPs, and that no cooperative (positive or negative) binding occurs for successive attachment of cage molecules to a given polymeric chain, thus meaning isolated equivalent binding epitopes. In other cases, only quenching of the Tyr monomer emission was observed (Figure 2C), and they were analyzed using the Stern–Volmer equation.^27^ The observed linear trends suggest a dynamic quenching process that could not be disentangled from the possible formation of supramolecular complexes. Thus, we can assume that any putative binding cannot be stronger than the observed quenching since the Sterm–Volmer constant (*K*_SV_) and the binding constant for a 1:1 complex would follow a similar dependence on the cage concentration.^28^ Accordingly, we estimated an upper limit for the interaction, therefore lower limit of *K*_d_ > 1/*K*_SV_ (entries 4, 7, 14, and 15 in Table 1). Two borderline cases were observed with measurements performed at different concentrations of some PPs, which allowed the accurate Stern–Volmer linear plot on a concentrated sample and observation of the lower energy-emitting species with diluted samples (entries 5 and 9 in Table 1). Alternative analysis of the different titration experiments to render either *K*_SV_ or *K*_d_ confirmed our initial hypothesis fulfilling the assumption that *K*_d_ > 1/*K*_SV_. Overall, despite the number of approximations assumed, the fluorescence titration experiments rendered a reasonable estimation of the apparent affinity of the cages toward Tyr residues surrounded by different amino acids in large PPs. Therefore, general trends can be extracted from the values depicted in Table 1.

**Table 1:** Apparent Affinity Values (*K*_d_, μM) for the Molecular Recognition of Tyr-Containing PPs by Pseudopeptidic Cages, Obtained by Fluorescence Emission Titrations (λ_exc_ = 276 nm, 50 mM Tris-HCl Buffer, and 293 K)^a^

entry	PP	cage	*K*_d_ (μM)	*K*_SV_ (M^–1^)	*T*_1ρ_ filter reduction (%)	*T*_2_ filter reduction (%)	*D* filter change (%)
1	polyE_4_Y	CyOrn	920 ± 24^b^				
2	polyE_4_Y	CyLys	450 ± 20^b^		98.1	100	80.3
3	polyE_4_Y	CyHis	61 ± 6		8.6	17.6	22.3
4	polyE_4_Y	CyAsp	>1225^c^	816 ± 16	–10.6	–18.9	17.3
5	polyE_4_Y	CyGlu	1738 ± 84	628 ± 17			
6	polyK_4_Y	CyOrn	no fit^d^	no fit^d^			
7	polyK_4_Y	CyLys	>825^c^	1212 ± 25			
8	polyK_4_Y	CyHis	93 ± 2		–8.3	–13.8	11.6
9	polyK_4_Y	CyAsp	398 ± 10	4048 ± 60	23.6	32.0	62.4
10	polyK_4_Y	CyGlu	400 ± 10				
11	polyE_6_K_3_Y	CyOrn	114 ± 2				
12	polyE_6_K_3_Y	CyLys	27.5 ± 0.6		31.9	41.1	34.6
13	polyE_6_K_3_Y	CyHis	194 ± 3		15.0	15.4	51.2
14	polyE_6_K_3_Y	CyAsp	>462^c^	2168 ± 36	0.3	5.3	8.2
15	polyE_6_K_3_Y	CyGlu	>526^c^	1902 ± 56			

a In the case of dynamic quenching, the systems were analyzed by Stern–Volmer plots (*K*_SV_, M^–1^). Quantitative analysis of the relaxation/diffusion-edited NMR experiments for the combination of the three cages (CyHis, CyLys, and CyAsp) with some of the three types of PPs (polyE_4_Y, polyK_4_Y, and polyE_6_K_3_Y), % values calculated for the aromatic proton resonances of the pseudopeptidic cages.

b From ref (24).

c Estimated lower limit assuming *K*_d_ > 1/*K*_SV_.

d The fluorescence titration experiments did not reliably fit any reasonable simple model.

The recognition of polyE_4_Y is stronger with cages bearing basic side chains (entries 1 and 2) than with acidic residues (entries 4 and 5). CyHis (entry 3) shows the strongest interaction with polyE_4_Y, as a result of the amphoteric nature of the imidazole ring, with a p*K*_a_ value close to neutrality that allows it to act as both an acid and a base at the pH used for the titrations. These results agree with the trends observed in the molecular recognition of Ac-EYE-NH_2_ by the same cages.^17^ This tripeptide can be considered the simplest Tyr binding epitope within polyE_4_Y. Titrations with the PP bearing basic residues (polyK_4_Y, Table 1, entries 6–10) rendered complementary trends with a more efficient interaction with CyAsp and CyGlu cages, again pivoting on the amphoteric CyHis. The data with polyE_6_K_3_Y (Table 1, entries 11–15) suggest that this PP globally behaves as an anionic species since the interaction seems stronger with positively charged cages. However, the results obtained with this random copolymer must be critically considered. The positive/negative complementarity of the side chains can promote aggregation and folding, possibly occluding Tyr side chains and making them inaccessible to the cages. Moreover, among the tested PPs, polyE_6_K_3_Y is the one showing statistically more heterogeneous Tyr residues, surrounded by either glutamic or lysine side chains. Then, the assumed approximations for the fitting procedures are scarcely applicable: the quantitative values in entries 11–15 of Table 1 are less reliable and should be used with caution.

Despite our simplified model, some general conclusions can be extracted from these experiments. First of all, fluorescence titration experiments are useful to screen pseudopeptidic cage-Tyr recognition in high molecular weight (*M*_w_) PPs. However, the results are more reliable when new lower energy-emitting bands are observed since dynamic quenching is also present, especially when the cage–Tyr interaction is weaker. For instance, this is illustrated by comparing the effect of a cage with acidic side chains, CyAsp, on two complementary PPs: dynamic quenching of anionic polyE_4_Y (Figure 2C), while the formation of a new emissive species with cationic polyK_4_Y (Figure 2D). On the other hand, the Tyr inclusion within the cage cavity is additionally modulated by the secondary interactions between the amino acids surrounding the Tyr in the peptide and the side chains decorating the pseudopeptidic cages (as also illustrated by Figure 2C,D and values in Table 1). This last conclusion agrees with the results obtained in previous studies with peptides of different lengths and sequences.^16,17,24^ Overall, these findings pave the way toward the selective molecular recognition of solvent-exposed Tyr residues in macromolecular PPs since the side chains of the cages establish noncovalent attractive/repulsive contacts with the amino acids in close proximity to the Tyr, thus mapping its chemical environment. The singular case of CyHis is noteworthy: despite it generally showing the strongest binding to the measured peptides, the amphoteric nature of the imidazole side chains reduces any potential sequence selectivity. Besides, we reported that CyHis also binds to Phe,^16^ possibly competing with the Tyr recognition in natural peptide sequences or proteins.

### 2.2 NMR Characterization of Pseudopeptidic Cages and Peptide Copolymers

#### 2.2.1 NMR Characterization of Pseudopeptidic Cages

In earlier studies, we observed low chemical shift perturbation of cages upon binding to a peptide.^17^ Thus, considering the different potential NMR parameters to monitor binding (chemical shifts, relaxation, and diffusion rates or nuclear Overhauser effects (NOEs)), we decided to evaluate changes in *T*_1_/*T*_2_ relaxation times and translational self-diffusion coefficients (*D*) of host cages upon binding to a guest. First, we measured the diffusion coefficients of the cages, and we calculated their hydrodynamic radii (*r*_H_) (Table S1) and overall correlation times (τ_C_) of 0.9 ns.^29^ These results reveal that the motions of the pseudopeptidic cages are near the region of minimum *T*_1_ at the magnetic field used (500 MHz) and do not satisfy the extreme narrowing conditions.^30^ Moreover, the results confirmed the monomeric nature of the cages in the aqueous solution. Next, we evaluated the need of water suppression for *T*_1_ and *T*_2_ measurements (Tables S2 and S3).^31,32^ Good fitting of the data was obtained only for the aromatic (singlet) and benzylic (doublets) proton signals using the standard CPMG pulse sequence,^30^ as J-modulation distorted the phase in the rest of the signals. The water residual signal also caused phase distortions and resulted in poor fitting for all of the signals. Thus, the best experimental results for relaxation rate fitting were obtained by water presaturation in a CPMG-PROJECT pulse sequence.^33^ Monoexponential decays were typically observed in CPMG and inversion recovery experiments for all of the ^1^H resonances. Calculated *T*_2_ and *T*_1_ values were in the ranges of 0.1–0.3 and 1.0–1.2 s, respectively (Tables S2 and S3). The relatively fast spin–spin relaxation (short *T*_2_) for those pseudopeptidic cages may be attributed to both long correlation times and chemical exchange contributions to spin–spin relaxation due to cage conformational flexibility and acid–base prototropic equilibria.

#### 2.2.2 NMR Parameters of PPs from Different Batches

Since polymer lengths and compositions may slightly differ between PP commercial batches, we wondered about their impact on the measured NMR parameters. Thus, the ^1^H *T*_1_/*T*_2_ and *D* values of two different commercial batches of polyE_4_Y were measured at different pHs and polymer concentrations. Remarkably, one of the two batches rendered shorter *T*_1_ and *T*_2_ regardless of sample concentration (Tables S4 and S5, entries 2 and 3 versus entries 5 and 6). We also observed small differences in measured diffusion coefficients between polyE_4_Y polymer batches (Table S6). In a random coil polymer chain, motions may be roughly classified as collective (involving motions of large portions of the chain, and including overall tumbling or rotatory diffusion) or local (involving only one or a few monomer units within the chain or side chains), so the application of the isotropic rotational motion model and its general dependence of the relaxation times (*T*_1_ and *T*_2_) on correlation time would be inaccurate.^34,35^ On the other hand, as we are comparing PP chains with the same amino acid compositions and local side chain motions and obtained monoexponential decays for all ^1^H measured resonances, we could consider a simplified model in which the general dependence of the relaxation times (*T*_1_ and *T*_2_) on correlation time is similar to the one observed for isotropic motions, even when a variety of motions are present. Thus, we postulate that the differences in relaxation time values between commercial batches are mainly caused by broadly different distributions of MWs of the polymeric chains. Moreover, these results are consistent with the different dependence on molecular size of relaxation rates and diffusion. Translational diffusion is inversely proportional to the hydrodynamic radius and therefore not a strong function of the *M*_w_ (*r*_H_ ∝ *M*_w_^1/3^, then *D* ∝ 1/*M*_w_^1/3^). Relaxation rates depend on τ_C_, which is proportional to the third power of the hydrodynamic radius (*r*_H_) of a molecule (assuming spherical molecules) and, therefore, is directly proportional to the *M*_w_ (τ_C_ ∝ *r*_H_^3^, then τ_C_ ∝ *M*_w_) of the polymers.

#### 2.2.3 Purification of Commercial PPs and NMR Characterization of Fractioned Samples

**Figure 3:**
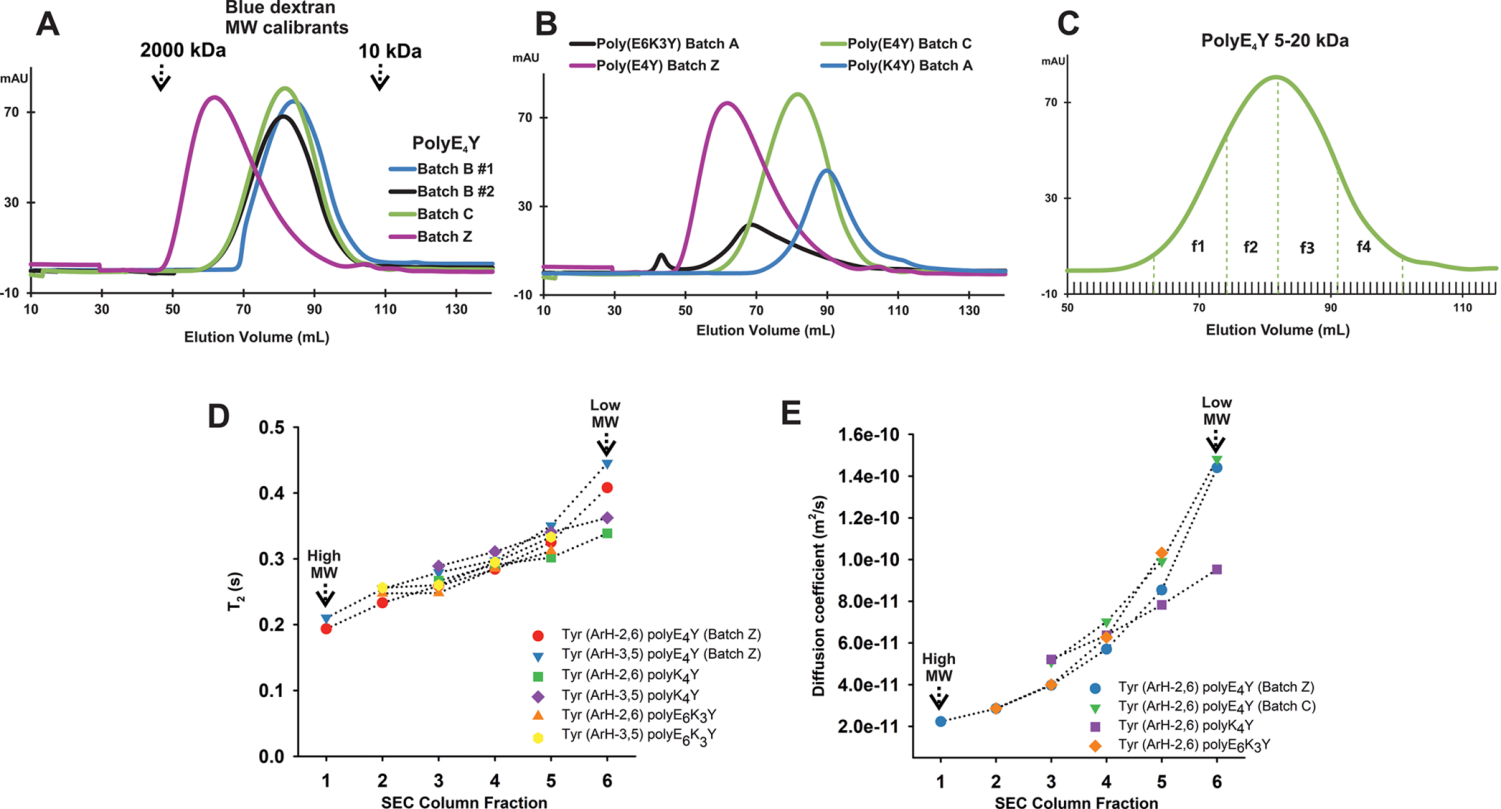
(A–C) SEC profiles of (A) three different batches (B/C (5–20 kDa) and Z (20–50 kDa)) of polyE_4_Y PP, the upper arrows indicate where Blue dextran 2000 and 10 kDa elute, (B) three tyrosine copolymers used in this work, and (C) polyE_4_Y (Batch C) with the four fractions collected for NMR analysis. (D, E) Representation of the variation of (D) *T*_2_ and (E) self-diffusion rate in collected fractions corresponding to the polymer samples of entries 2–4 in Table 2. To account for the differences in *M*_w_, greater for polyE_4_Y and polyE_6_K_3_Y than for polyK_4_Y, fractions (3–6) of polyK_4_Y on the (D) and (E) graphs correspond to collected fractions 1–4.

Taking into account the variability between commercial batches detected by NMR, we purified the commercial PPs to obtain less polydispersed fractions and, therefore, samples with more homogeneous NMR characteristics. A size-exclusion column (SEC) designed for protein MW separation in the range of 10–600 kDa was used with UV detection at 280 nm. Figure 3A,B illustrates the SEC separation of the PPs, showing the appearance of very broad peaks during elution, an observation compatible with the polydisperse nature of commercial polymer batches. We combined SEC with diffusion-ordered NMR spectroscopy to separate different fractions with less polymer polydispersity and characterize their MW with higher accuracy.^36,37^ To estimate the average *M*_w_s of the purified PP fractions, we built an *M*_w_ calibration function by measuring NMR diffusion coefficients of commercial dextran analytical standards with SEC quality (with known and narrow *M*_p_, the *M*_w_ of the highest peak; Figure S17). For each batch, several fractions were collected (Figures 3C and S18), concentrated, buffer exchanged, and *T*_1_, *T*_2_, and D NMR parameters were measured (Tables S7–S10) for all fractions of the three PPs. All these NMR measurements were acquired at the same low polymer concentration (0.6 mM in Tyr) to avoid aggregation, viscosity changes, or interference between polymer molecules. The measured diffusion coefficients were almost identical when comparing the same collected fractions for all 5–20 kDa polyE_4_Y three runs (B#1, B#2 and C, blue/black/green lines in Figure 3A), showing excellent separation reproducibility (Table S7). If we compare the diffusion coefficients of the individually collected fractions with the value for a sample prior to SEC, we qualitatively confirm the broad distribution of diffusion coefficients (*M*_w_s) present in the unfractioned samples (Table S7A versus Table S7B–D values). As shown in Table 2, our diffusion measurements showed some discrepancies between the estimated MWs and those provided by the supplier (see Tables S11–S13 for details).

**Table 2:** Commercial PP *M*_w_ Ranges after Characterization by Diffusion NMR and Fractions Used for NMR Interaction Studies (Coefficient Diffusion Values for Each Fraction Listed in Tables S11–S13)

entry	PP	*M*_w_ supplier (kDa)	batch	*M*_w_ experimental by SEC + DOSY (kDa)	fractions for NMR interaction (*M*_p_, kDa)
1	polyE_4_Y	5–20	C	3–26	
2	polyE_4_Y	20–50	Z	3–154	f3 (49)
3	polyK_4_Y	20–50	A	7–27	f1 (27)
4	polyE_6_K_3_Y	20–50	A	7–100	f3 (46)

Furthermore, considering the results for polyE_4_Y high MW batch (3–154 kDa), with sufficient material in each isolated fraction to measure NMR parameters of all of them, we observed that the measured *T*_1_ was mostly constant over all collected fractions whereas *T*_2_ increased with fraction number (i.e., *T*_2_ decreasing with higher *M*_w_s, Figure S22). Graphic representations of *T*_2_, diffusion coefficients, and *T*_1_ measured for tyrosine aromatic protons for each collected fraction of the three PPs are shown in Figures 3D,E and S25, respectively. In Figure S26, we represent the *T*_1_ or *T*_2_ values for selected Tyr and Glu resonances of polyE_4_Y for comparison purposes. For further NMR experiments, we selected those PP fractions with higher *M*_w_s to maximize the spectroscopic changes of cages upon binding and with more similar NMR observed parameters to be able to compare the results between different PPs (Table 2).

### 2.3 Characterization of Cage-PP Binding by NMR

The chemical composition of tyrosine PPs (only two or three different amino acids in a high *M*_w_ random copolymer) and the derived poor resolution of their NMR spectra complicate the application of a typical biopolymer-based NMR approach to detect binding. Alternatively, we decided to apply NMR ligand-based methods (STD-NMR, *T*_1_/selective *T*_1_/*T*_2_/*T*_1ρ_/diffusion filters or waterLOGSY)^38^ to evaluate those intermolecular interactions. In the present case, the realization of STD-NMR experiments was troublesome due to the overlapping of cage and PP resonances, precluding selective excitation of the biopolymer. Moreover, some of the cage-PP relative concentrations needed for waterLOGSY experiments showed unsuitable turbidity and/or precipitation (20:1 cage:PP, see Tables S16 and S17). Additionally, as a consequence of *T*_1_ dipolar relaxation specific dependence on correlation time, larger and medium-sized molecules may show similar *T*_1_ values.^30^ This is observed when comparing aromatic protons of the CyLys cage (Table S2) with tyrosine protons of polyE_4_Y (Table S8). Alternatively, selective *T*_1_ relaxation, where only one nucleus is excited selectively in each measurement, shows a dependence on correlation time similar to *T*_2_ and could be applicable to study cage binding to PPs. However, as for STD-NMR, selective excitation is not accessible in our host–guest mixtures.

**Figure 4:**
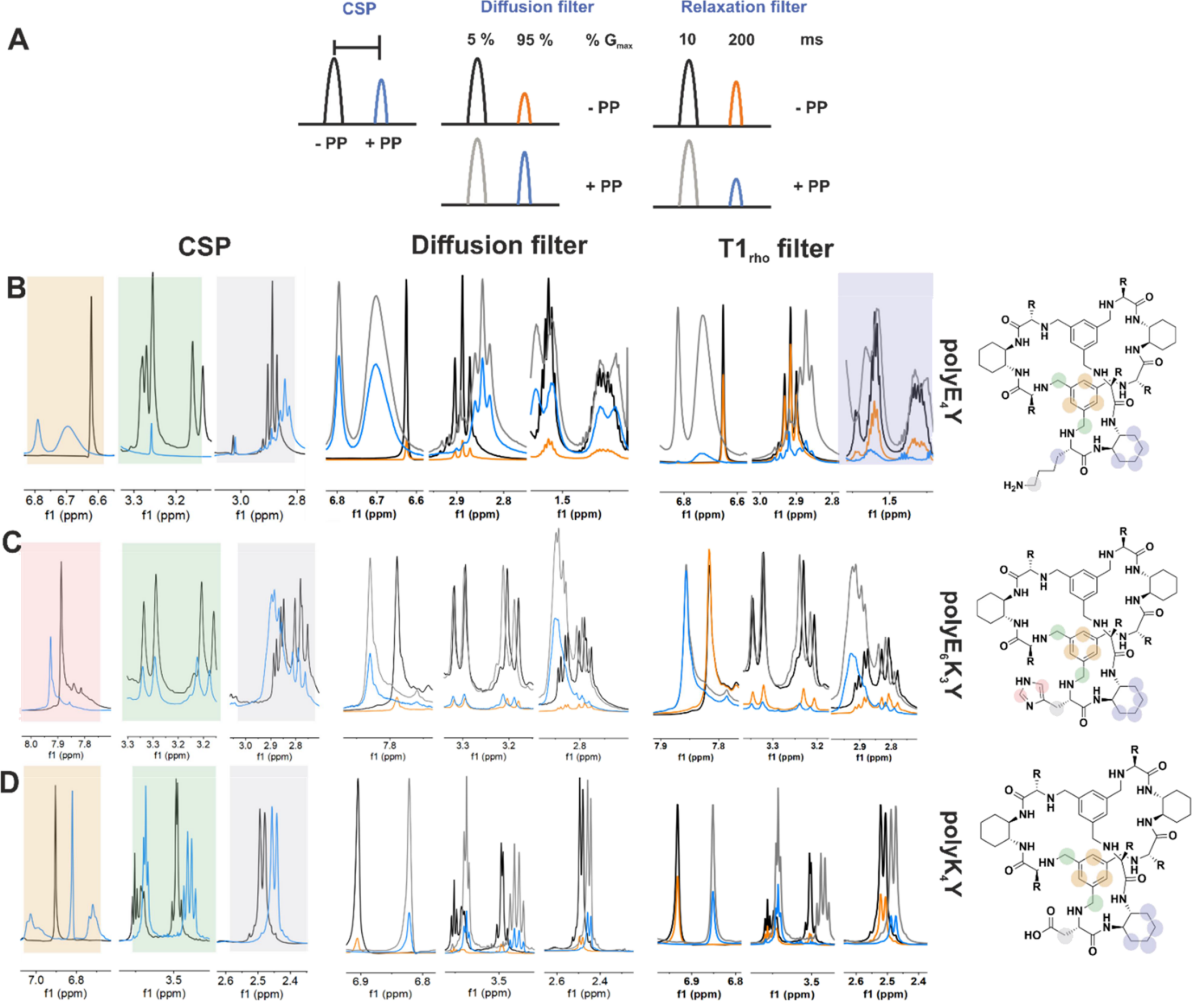
(A) Schematic representation of the NMR experiments used to identify the binding of cages to PPs. (B–D) Analysis of cage binding to PPs using chemical shift changes and relaxation/diffusion-edited approaches. For chemical shift changes (CSP), black (without PP)/blue (with PP). For diffusion/relaxation filters: black/orange, cage without PP spectra, low field gradient (5%) or short *T*_1ρ_ filter (10 ms)/high field gradient (95%) or long *T*_1ρ_ filter (200 ms); gray/blue, cage with PP spectra, low field gradient (5%) or short *T*_1ρ_ filter (10 ms)/high field gradient (95%) or long *T*_1ρ_ filter (200 ms). (B) CyLys/polyE_4_Y; (C) CyHis/polyE_6_K_3_Y; and (D) CyAsp/polyK_4_Y. The shifted protons are mapped out on the cage structures.

In view of the previous observations and the values measured for cages and PPs alone, we tried diffusion- and *T*_2_/*T*_1ρ_ relaxation-based approaches for identifying these interactions (Figure 4A). We measured *T*_2_ relaxation times and translational self-diffusion coefficients for the combination of three cages (CyLys, CyHis, and CyAsp) with three selected fractions of PPs (Table 2). In order to obtain the largest possible effects on the cages, the PPs were used in stoichiometric amounts or even in slight excess (based on Tyr concentration), which was also favorable in terms of solubility (Tables S16 and S17). In this way, (1) the fraction of bound cage and the detection of changes in the NMR spectra are maximized, allowing to observe both relatively weak and strong interactions and (2) we avoid the problem of working with ligands (and observed resonances) in excess, situation in which a small population of a bound signal with slow binding kinetics could be undetectable.^38^ From these measurements, we detected changes in *T*_2_ or diffusion coefficient values (in bold, Tables S18 and S19) for several cages/PP combinations compared with the cages without guest in solution and hypothesized that these differences were enough to acquire 1D NMR experiments with relaxation/diffusion filters to detect binding.

Relaxation/diffusion filters unavoidably reduce spectral sensitivity due to the delays or pulse and gradient elements included in the NMR pulse sequences. To correct this effect, we also acquired reference spectra of each host cage alone, with short/long relaxation times and low-/high-gradient filters (Figure 4A).^39^ Selected results are shown in Figure 4B–D, and the rest are shown in Figures S27–S30, where we compare the *T*_1ρ_-relaxation (10 and 200 ms) and diffusion-filter-based 1D ^1^H spectra (5 and 95% maximum Gz gradient) for cages alone and for the mixture of host cage and guest PP. We calculated the percentage of signal loss due to relaxation/diffusion-filtered ^1^H experiments (Table 1) and the trends observed are in agreement with the differences observed when comparing the measured parameters (Tables S18 and S19). These results are also qualitatively summarized in Table 3, including the evaluation of observed chemical shift perturbations (CSP).

**Table 3:** Qualitative Evaluation of CSP or Effects in ^1^H 1D Spectra with Relaxation/Diffusion Filters^a^

cage	polyE_4_Y			polyK_4_Y			polyE_6_K_3_Y		
	CSP	relaxation	diffusion	CSP	relaxation	diffusion	CSP	relaxation	diffusion
CyLys	**+++**	**+++**	**+++**	nm	nm	nm	**++**	**++**	**+**
CyHis	+	**+**	**–**	–	–	–	++	+	++
CyAsp	++	–	–	**+**	**++**	**++**	+	–	–

a nm, not measured; −, no changes; and + to +++, low-to-high changes in NMR spectra. (+ to +++) In agreement with established intensity loss levels in the Materials and Methods section and Table 1: diffusion, no binding <25%, weak <50%, medium <80%, and strong >80%; relaxation, no binding <15%, weak <40%, medium <60%, and strong >60%.

Filtered 1D ^1^H NMR experiments in the absence or presence of the PPs can be used to screen quickly the binding of different pseudopeptidic cages. The effects in *T*_1ρ_-(or CPMG)-based experiments are influenced by both binding constant and the relaxation rate of every observed proton. Since the relaxation rates vary between different protons of the same cage and between similar protons of different cages (Table S19), these experiments do not directly reflect affinity ranking or epitope mapping. For diffusion-based experiments and interactions in the fast exchange regime, there is a direct correlation between affinity and changes in signal intensity, due to the exclusive dependence on the bound cage fraction of the diffusion rate (which will be population-weighted). As the PP fractions for polyE_4_Y and polyE_6_K_3_Y have similar *M*_p_ (Table 2) and the binding is in the fast exchange regime, diffusion-based experiments could be used to rank the cage–PP interaction according to their affinity.

The most noticeable changes in CyLys cage chemical shift in the presence of polyE_6_K_3_Y are seen for the aromatic and benzylic resonances, shifting 0.08–0.09 ppm downfield and broadening significantly (Figure S30A). In the presence of polyE_4_Y, the aromatic protons resonances of CyLys shift 0.17 ppm downfield and the peaks for the benzylic protons broadened to become barely visible (Figure 4B). CyHis aromatic signals moved slightly downfield in the presence of polyE_4_Y or polyK_4_Y, but the chemical shift of the rest of resonances was not affected (Figures S27D-I and S28D-I), unlike in the presence of polyE_6_K_3_Y (Figure 4C). The hot spots in the binding of CyHis to this copolymer were the protons of the His side chain (CH_2_β, CH ε_1_/δ_2_) and aromatic protons. We also detected a significant peak height reduction of CyHis ^1^H 1D-unfiltered spectrum resonances in the presence of polyE_6_K_3_Y PP but not with polyE_4_Y or polyK_4_Y (Figures 4C and S27D,G and S28D,G). 1D ^1^H *T*_1ρ_-relaxation and diffusion-filter-based experiments revealed the binding of CyHis to polyE_6_K_3_Y and polyE_4_Y, while the interaction with polyK_4_Y was not detected with this technique (Figures S27E,F and S28E,F).

For the CyAsp cage, we unexpectedly observed an increase in intensity and a large chemical shift change in the presence of polyE_4_Y, in particular for cage aromatic resonances (Figure S29G). Additionally, in relaxation filter-based ^1^H 1D proton experiments, we also observed a small intensity increase for the CyAsp/polyE_4_Y sample (Table 1 and Figure S29H), compatible with a small increase in *T*_2_ (Table S19, entries 1 and 2). Finally, we observed too small changes in the diffusion-filtered spectra to be ascribed to binding, which was confirmed by measuring the diffusion coefficients of the samples (Table S18, entries 1 and 2). Accordingly, these results seem to indicate a weak interaction between CysAsp and polyE_4_Y. This inversion of relative intensities was not observed for CyAsp in the presence of polyE_6_K_3_Y or polyK_4_Y. We detected small chemical shift changes for CyAsp in the presence of polyE_6_K_3_Y that did not correlate with changes in intensity in the 1D ^1^H filtered experiments (Figure S29A–C). Finally, filter-based screening experiments clearly showed the interaction between CyAsp and polyK_4_Y (Figure 4D).

Our results show that a combination of several NMR screening experiments is recommendable to assess binding between medium-sized hosts and polymeric substrates. Besides, relaxation and diffusion-filtered NMR experiments seem more suitable than CSP. For instance, the observation of chemical shift changes for CyAsp cage protons after adding polyE_4_Y but no relevant peak intensity changes for diffusion or relaxation-based experiments indicates poor binding (Figure S29G–I), as also detected by fluorescence titrations. Most likely, the observed CSP in this case might be related to different interactions with positively charged ions in this sample (buffer and salt), which has a stronger impact on chemical shifts than on relaxation or diffusion parameters.^40^

If we globally analyze the NMR results in Tables 1 and 3, strong-medium binding is detected with the following cage-PP combinations: [CyLys-polyE_4_Y], [CyAsp-polyK_4_Y], [CyLys-polyE_6_K_3_Y], and [CyHis-polyE_6_K_3_Y], which are in good agreement with the fluorescence titration experiments (Table 1). On the other hand, the NMR experiments with [CyAsp-polyE_4_Y] and [CyAsp-polyE_6_K_3_Y] suggest a much weaker interaction, also in line with the fluorescence data (Table 1). The [CyHis-polyE_4_Y] interaction was apparently weaker by NMR than by fluorescence, and a clear disagreement was obtained in the case of [CyHis-polyK_4_Y] since the binding detected by fluorescence was undetectable by NMR. These two last observations may suggest that the CyHis cage produces a strong effect in Tyr emission that could overestimate fluorescence changes upon titration. Actually, a strong stabilization of excited-state tyrosinate by the proximal His residue has been reported for angiotensin II analogues.^41^ Alternatively, unfavorable on/off kinetics of these complexes could hinder their accurate detection by NMR. Despite that, NMR (Table 1 and Figure 4) and fluorescence spectroscopy (Table 1) studies show the same general trends that the Tyr inclusion within the cage cavity is further enforced by attractive polar interactions between the respective side chains of cages and PPs.

### 2.4 Structure of the Cage-PP Supramolecular Complexes

**Figure 5:**
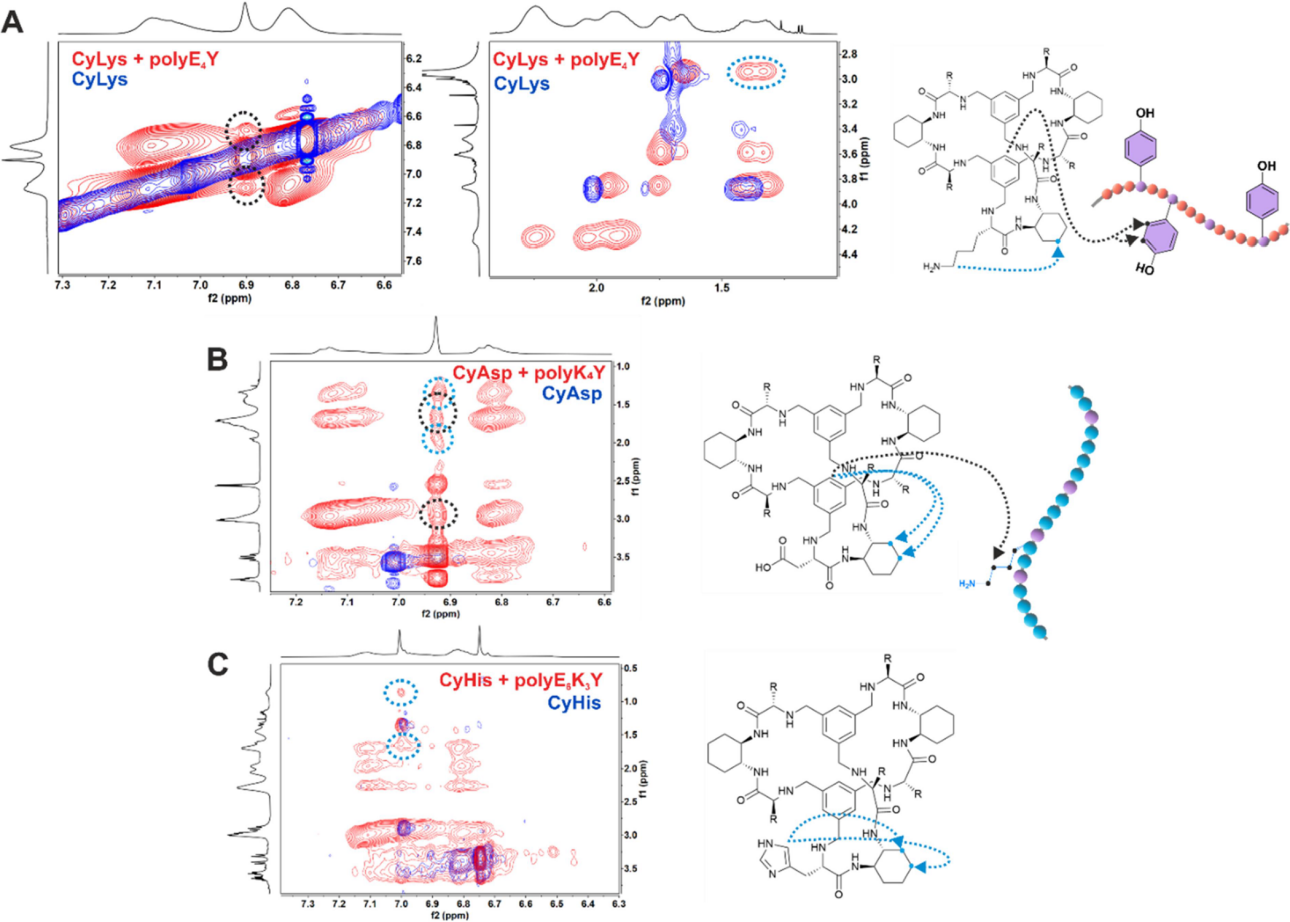
Superimposed 2D ^1^H–^1^H NOESY spectra of cages alone (blue) and in the presence of tyrosine copolymers (red). (A) [CyLys-polyE_4_Y], (B) [CyAsp-polyK_4_Y], and (C) [CyAsp-polyE_6_K_3_Y]. New cross-peaks are marked with black (intermolecular) or blue (intramolecular) dotted circles. The protons involved are represented with black and blue arrows in the represented structures.

To get additional information about the solution structure of host–guest complexes, we acquired 2D ^1^H–^1^H NOESY experiments of the cage-PP mixtures versus those of the cages alone. For CyLys and polyE_4_Y, we found two new cross-peaks associated with close contact between the aromatic protons of CyLys and polyE_4_Y (intermolecular; black dotted line in Figure 5A) and between Lys Hε and cyclohexyl CH_2_ protons (intramolecular; blue dotted line in Figure 5A). The intermolecular NOE effect here observed confirms the inclusion of the Tyr side chain within the cage cavity and is in good agreement with the fluorescence emission observations. For CyAsp in the presence of polyK_4_Y (Figure 5B), cross-peaks were observed between the cage aromatic and protons in the region of 1.2–3.0 ppm, corresponding to the cyclohexane moiety (intramolecular NOE) and lysine side chain methylenes (intermolecular NOEs). For CyHis in the presence of polyE_6_K_3_Y, we only observed two unambiguous additional cross-peaks, between the δ proton of the histidine side chain and cyclohexyl CH_2_ protons (Figure 5C). These observed new intramolecular contacts suggest cage conformational change on binding to the PP.

**Figure 6:**
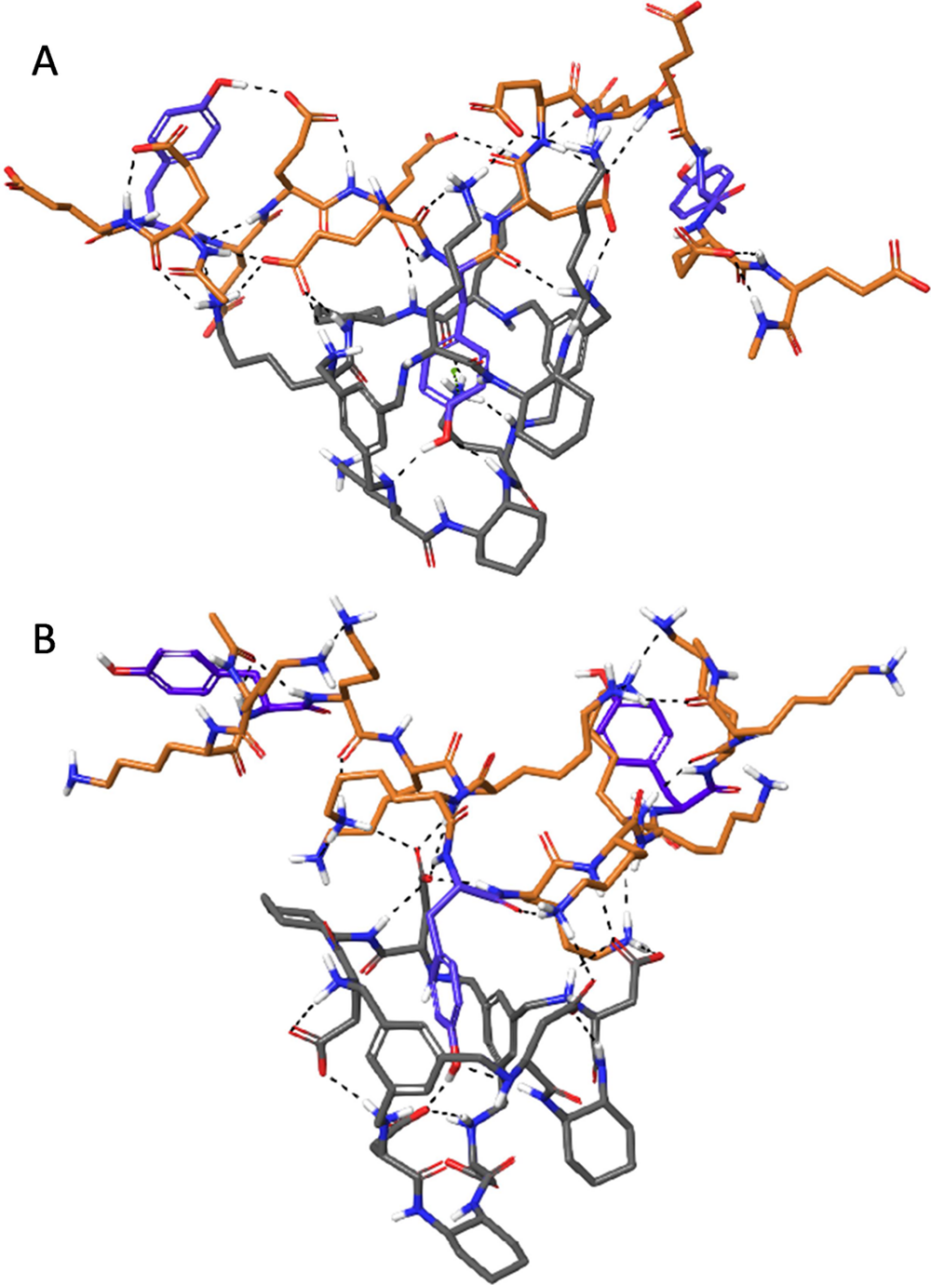
Optimized model structures for the (A) [CyLys-polyE_4_Y] and (B) [CyAsp-polyK_4_Y] complexes. For clarity, nonpolar H atoms are omitted. Color code of C-atoms: cage in gray, PP in orange, and Tyr in purple. H-bonds are shown as black dashed lines.

In order to obtain a more detailed picture of the supramolecular complexes, we performed molecular modeling calculations with a simplified model in two cases where the supramolecular complexes were unambiguously characterized with both experimental techniques including key intermolecular NOEs, namely, [CyLys-polyE_4_Y] and [CyAsp-polyK_4_Y]. As PP binding motives, we used sequences of the type Ac-XX**Y**XXXX**Y**XXXX**Y**XX-NHMe, where **Y** states for Tyr and X for either Glu or Lys amino acids. Thus, we constrained the PP to three of the most probable repeating units in each case, capping with acetyl and *N*-methylamide at the N and C termini, respectively. We manually docked the corresponding cages (either CyLys or CyAsp) to the central Tyr and performed Monte Carlo conformational searches in implicit water with OPLS4 force field minimizations as implemented in the Macro Model. The global minima thus located are shown in Figure 6.

Several general conclusions can be extracted from these cases. First of all, the bound Tyr residue remains within the cage cavity in both cases, establishing attractive interactions with the cage core and side chains. For instance, a cation-π contact is established between the Lys side chain of CyLys and the Tyr aromatic ring of polyE_4_Y (Figure 6A). In the two modeled examples, the Tyr hydroxyl is H-bound to several cage functional groups (see Figure 6A,B), explaining the lower energy fluorescence emission by a strongly polarized Tyr group. Besides, the distances between the host–guest aromatic rings in the [CyLys-polyE_4_Y] complex (Figure 6A) are in good agreement with the observed intermolecular NOE (Figure S33). The intermolecular NOE detected in the [CyAsp-polyK_4_Y] complex is also consistent with the structure in Figure 6B, as depicted by the distances between a Lys side chain of the polymer and the aromatic core of CyAsp (Figure S36). Moreover, the two new intramolecular NOEs detected in both complexes can be also explained by interproton distances <5 Å in the located minima (Figures S33 and S36). Regarding the secondary host–guest side chain-side chain polar interactions, both complexes show many carboxylate-ammonium salt bridges and H-bonds in the simulations, thus supporting the conclusions extracted from the experiments. For both optimized complexes, the recognition of the central Tyr leaves the other two proximal Tyr residues exposed enough for successive binding of additional cages, somehow supporting our initial assumption of independent equivalent epitopes (Figures S32 and S35). Additionally, the truncated models with the minimal expression of the binding epitopes (corresponding to Ac-XX**Y**XX-NHMe peptides) showed a high similarity with the optimized structures depicted in Figure 6 (see Figures S37–S38) suggesting that the flexibility of the free PP moiety would have a minimal impact on the interaction site.

## 3 Conclusions

In this work, we combined two experimental techniques to study a specially challenging supramolecular system in an aqueous solution: pseudopeptidic cages (medium-sized hosts) and PTK PP substrates (large multivalency guests). Three polymers (polyE_4_Y, polyK_4_Y, and polyE_6_K_3_Y) were selected, which have been previously used to analyze binding and specificity in closely related kinases such as c-Src and Lck. The Tyr residue from the PPs is a convenient fluorescence probe for detecting and quantifying the binding through titration experiments, rendering a new emissive species, while the possibility of dynamic quenching must be carefully considered. As a complementary technique, NMR allows the study of complexes through simple and fast experiments. However, the *M*_w_ heterogeneity of commercially available PPs is a drawback in NMR experiments, and accordingly, the purification to less polydispersed fractions is mandatory in this case. We found that to identify binding selectivity on different cage–PP combinations, changes in translational self-diffusion rates and relaxation times are more reliable parameters than chemical shift perturbation. Our results confirm that diffusion or relaxation-based filtered 1D ^1^H NMR experiments allow to study the binding of a pseudopeptidic cage (a medium-size molecule) to a high *M*_w_ guest (PPs). Moreover, key intermolecular NOEs further support the formation of supramolecular complexes in solution. We used all the experimental results to propose a reasonable mode of binding, where the recognition of Tyr residue in PPs is modulated by the complementary cage–PP side chain electrostatic interactions. We concluded that the suitable characterization of these challenging supramolecular complexes requires a wise combination of both fluorescence and NMR since in this case they have been shown to be complementary in practice.

## 4 Experimental Section

### 4.1 Fluorescence Titration Experiments

Fluorescence emission spectra were acquired on a Photon Technology International Instrument, the Fluorescence Master Systems, with an excitation bandwidth: 9 nm, emission bandwidth: 15 nm, light source: Xenon flash lamp (1 J/flash), and emission read every 1 nm. All the fluorescence experiments were performed at 20 °C in cuvettes with a 10 mm path length. The different PP-cage titrations were conducted in a 700 μL fluorescence cuvette following a protocol similar to those previously described.^16,22^ A solution of the peptide copolymer (200 or 20 μM in the repeating units) was prepared in buffered water (50 mM Tris-HCl, pH 7.5). 300 μL of the PP solution was titrated with a solution of the cage (1–4 mM) in buffered water (50 mM Tris-HCl, pH 7.5) containing the titrated PP at the same concentration to keep it constant throughout the whole titration. The PP concentration refers to that of the polymer repeating unit (equal to the Tyr residue concentration), and it was adjusted for each titration considering the observed fluorescence changes, solubility issues, and the possibility to obtain meaningful experimental points for the fitting. The excitation wavelength was λ_ex_: 276 nm and the recorded emission window was adjusted for each PP to observe a representative part of the emission band for the excimer (typically 290–500/550 nm). Replicates were carried out to ensure reproducibility, and for selected examples, different concentrations of PP were assayed. HypSpec software (http://www.hyperquad.co.uk/HypSpec.htm) was used to fit the fluorescence titration data to a simplified interaction model (1:1 with respect to the Tyr residues). This software performs the global fitting of the whole emission band (or a selected range) for each titration point, to satisfy the interaction model in each case and render the global formation constants of the corresponding complexes (Log β).^42,43^ When only quenching of the Tyr emission was observed, we fitted the emission maximum to the Stern–Volmer equation (*F*_0_/*F* = 1 + *K*_SV_ [cage]).^27^ The fluorescence titration experiments of polyE_4_Y with CyLys and CyOrn have been reported,^24^ although a different nonlinear regression method was used to fit the data. Fitting those titrations and new replicates with HypeSpec led to the same results as those reported (within the confidence range). The corresponding fluorescence emission titration spectra and fitting curves at selected wavelengths (the software uses all wavelengths within a considered range) are shown in Figures 2 and S1–S16. For most of the cases, the titration data was satisfactorily fitted to a simple 1:1 model considering the Tyr residues as equivalent isolated binding epitopes.

### 4.2 Purification of Commercial polyE_4_Y, polyK_4_Y, and polyE_6_K_3_Y

Three different types of PPs were purchased from Sigma-Aldrich: Poly(Glu, Tyr) sodium salt (Glu:Tyr 4:1, *M*_w_ 5000–20,000 and 20,000–50,000), Poly(Lys, Tyr) hydrobromide (Lys:Tyr 4:1, *M*_w_ 20,000–50,000), and Poly(Glu, Lys, Tyr) sodium salt (Glu:Lys:Tyr 6:3:1, *M*_w_ 20,000–50,000). Blue Dextran 2000 was part of the Gel Filtration Calibration Kit purchased from GE Healthcare and Blue Dextran 10 was purchased from Sigma-Aldrich, both used for the initial calibration of the SEC column. Commercial dextran analytical standards for SEC and poly(4-styrenesulfonate acid) (PSS) sodium salt were also purchased from Sigma-Aldrich.

The PPs were purified by SEC using a HiLoad 16/600 Superdex 200 pg column on a KTA Purifier system (Cytiva Life Sciences). The conditions for PP purification were changed depending on the PP composition. PolyE_4_Y was eluted with the same buffer used for NMR measurements (15 mM HEPES and 50 mM NaCl, pH 7.4). PP polyK_4_Y and polyE_6_K_3_Y got stuck to the column under these conditions. Thus, after trying different settings, we could purify them in 100 mM phosphate, 150 mM NaCl at pH 3.5 for the former, and the same buffer at pH 6.2 for the latter. PP samples of 1 mL were injected and a flow rate of 1 mL/min was used. PP elution was followed by a coupled UV detector by measuring the absorbance at 280 nm. The eluted PP was collected in fractions of 1 mL. These smaller fractions were put together into samples of around 8–10 mL, to obtain four (in the case of low *M*_w_ polyE_4_Y and polyK_4_Y) or six (in the case of high *M*_w_ polyE_4_Y and polyE_6_K_3_Y) larger fractions, as shown in Figures 3C and S18. To prepare the PPs for analysis by NMR, the obtained fractions were concentrated using Amicon Ultra-4 (cutoff of 3000 Da) centrifugal units (Merck Millipore) by washing first with H_2_O and then with D_2_O to 500 μL. The PP concentrations of these stock solutions were determined from Abs_280_ measurements on a NanoDrop 8000 (Thermo Fisher Scientific) and were calculated at the molar concentration of tyrosine. The samples were diluted as needed and HEPES-d_18_ and NaCl were added to have a final concentration of 0.6 mM PP, 15 mM HEPES-d_18_, 50 mM NaCl, and pH 7.0 for all samples.

Three SEC runs of the 5–20 kDa samples (batch B × 2 injections and batch C × 1 injection) and one batch of 20–50 kDa (batch Z) were completed for polyE_4_Y (Figure 3A), which allowed us to assess reproducibility despite differences between commercial batches with the same and different *M*_w_ ranges. We selected commercial dextrans with average *M*_w_ over the range of purchased PPs (from 5 to 70 kDa) and, additionally, PSS, a charged and linear aromatic polymer, more similar to tyrosine polyamino acids. The measured self-diffusion coefficients for the dextran standards were in agreement with previously published results and followed a linear relationship between log *D* and log *M*_p_ (Figure S17).^36^ The calculated regression equation was used as a calibration function for the evaluation of the tyrosine copolymer *M*_p_ by measuring the corresponding diffusion coefficients of the collected SEC fractions. We were aware that the solution structure of branched dextran polysaccharide and the linear PPs differ, but the need to have *M*_w_ polymer standards commercially available, soluble in water, and with a diverse and narrow range of *M*_w_s was a limiting factor. In any case, the lateral branches of dextran molecules from *Leuconostoc* spp. usually consist of one or two glucose residues; as previously described, those dextrans had less than 5% α-(1 → 3) branch.^44^

### 4.3 NMR Spectroscopy

The NMR experiments were carried out on a Bruker Avance III HD spectrometer operating at 500 MHz (^1^H resonance frequency), using a 5 mm helium-cooled TCI (^1^H/^13^C/^15^N) cryoprobe equipped with a z-gradient coil (55 G/cm). NMR spectra were acquired using Bruker TopSpin 3.6 and processed with Bruker TopSpin 4.0 and MNova 14 software (Mestrelab Research). All the relaxation and diffusion data were analyzed using Bruker Dynamics Center 2.5.

The samples for the analysis of the NMR relaxation and diffusion properties of the pseudopeptidic cages and the PPs were prepared to have a final concentration of 0.5–2.0 mM of a given compound in D_2_O, 15 mM HEPES-d_18_, and 50 mM NaCl, pH 7.0. The exact composition of the prepared samples is specified in theSection 2 for each case. All of them were prepared in Shigemi NMR tubes and acquired at 298 K. The pulse programs used for spectra acquisition were zggpw5 (1D ^1^H with water suppression using Watergate W5 pulse sequence) and stebpgp1s19 (2D sequence for diffusion measurement using stimulated echo and bipolar gradient pulses, Δ = 120 ms, δ = 2 ms, the gradient strength was incrementally increased in a linear manner from 5 to 95% of the maximum gradient strength) from Bruker’s pulse sequence library, and 2dt1irpr_cwvd (*T*_1_ measurement using inversion–recovery with continuous wave excitation for water presaturation during the relaxation delay and the variable τ delay) and project_cpmgpr2d (pseudo 2D sequence for *T*_2_ measurement using the CPMG-PROJECT pulse sequence for J-modulation suppression with added water presaturation) from the literature.^33,45^

The samples for the analysis of binding between the pseudopeptidic cages and the purified PPs were composed of 0.4 mM CyLys, CyAsp, or CyHis and either an equimolar amount or an excess of polyE_4_Y (fraction 3Z), polyK_4_Y (fraction 1A), or polyE_6_K_3_Y (fraction 3A) in D_2_O, 15 mM HEPES-d_18_, 50 mM NaCl, pH 7.0–7.5 (Figure S18). The pulse programs used for spectra acquisition were noesyfpgpphwg (2D ^1^H–^1^H NOESY with Watergate water suppression, mixing time = 100 ms), t1rho_esgp2d (pseudo 2D *T*_1ρ_-filtered ^1^H, two spectra acquired with 10 and 200 ms filter), cpmg_esgp2d (pseudo 2D *T*_2_-filtered ^1^H, two spectra acquired with 10 and 300 ms filter) and stebpgp1s191d (1D diffusion-filtered ^1^H, two spectra acquired with 5 and 95% gradient strength, Δ = 120 ms, δ = 2 ms) from Bruker’s pulse sequence library, in addition to the experiments mentioned in the previous paragraph.

The *T*_1ρ_/*T*_2_/diffusion filter reduction/change is the partial loss of ratios from relative peak integrals between 200/300 ms or 95% Gz and 10/10 ms or 5% Gz of a proton signal in the *T*_1ρ_/CPMG or diffusion 1D ^1^H spectra of the fragment in the presence and absence of the PP (or vice versa in the case of the diffusion filter) given as percentage and is calculated using the following equation.^46^ If the percentage of reduction of a cage in the presence of PP is ≥15% (relaxation filter) or >25% (diffusion filter), then it is considered as a binder (Table S20):
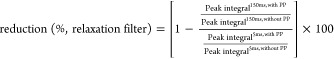

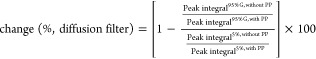


To reduce integration errors due to the presence of excessive noise or nearby peaks, we used Mnova deconvolution (line fitting feature) for accurate spectral integration of the cage aromatic Tyr protons. Qualitatively, we have defined an arbitrary scale to compare the different experiments (Table 3). For diffusion, no binding <25%, weak (+) <50%, medium (++) <80%, and strong (+++) >80%; relaxation, no binding <15%, weak (+) <40%, medium (++) <60%, and strong (+++) >60%.

The diffusion values obtained from the NMR measurements of the pseudopeptidic cages were used to calculate their corresponding hydrodynamic radius, *r*_H_. Two different methods were applied, and the results are compared in Table S1. The first approach was to calculate the *r*_H_ with the Stokes–Einstein equation, which assumes that the solute acts as a hard sphere with the hydrodynamic radius *r*_H_, at an infinite dilution in a continuum fluid with the viscosity η. To calculate the diffusion coefficient *D*, the thermal energy of the system (*k*_B_*T*, where *k*_B_ is the Boltzmann constant and *T* is the temperature) is balanced by the friction acting on the particle:
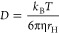


The Stokes–Einstein equation can give good estimates for the diffusion coefficients of large species (nanometers and larger) but does not work as well for smaller molecules due to the limitations of the model (molecules are not hard spheres moving through a continuous fluid). The Stokes–Einstein–Gierer–Wirtz estimation (SEGWE) is a data-based method that obtains better predictions for small molecules.^47,48^ The spreadsheet made available by the Manchester NMR Methodology Group (https://www.nmr.chemistry.manchester.ac.uk/) was used for the calculation of the hydrodynamic radius using this second approach.

## Supporting Information Available

Fluorescence spectroscopy experiments: spectra and titration fittings; NMR characterization of pseudopeptidic cages: diffusion and relaxation data; NMR characterization of polypeptides, before and after size exclusion chromatography: diffusion and relaxation data; NMR studies of pseudopeptide cage binding to polypeptides using chemical shift changes and relaxation/diffusion-edited approaches; and molecular models of cage-polypeptide complexes (PDF)
