# Supplementary material for: Molecular Recognition of Tyrosine-Containing Polypeptides with Pseudopeptidic Cages Unraveled by Fluorescence and NMR Spectroscopies
Source: Bioconjug Chem. 2023 Dec 11;34(12):2345–57. doi: 10.1021/acs.bioconjchem.3c00455 (PMC10859922; doi:10.1021/acs.bioconjchem.3c00455)
Supplement: Supplementary file 1 — bc3c00455_si_001.pdf [file bc3c00455_si_001.pdf]

Supporting Information for:

Molecular recognition of tyrosine-containing polypeptides with pseudopeptidic cages unraveled by fluorescence and NMR spectroscopies

Naiara Solozabal,[a,]† Lucía Tapia,[b,]† Jordi Solà,[b] Yolanda Pérez,[a,] Ignacio Alfonso[b,]**

†Equal contribution

a. NMR Facility, Institute for advanced Chemistry of Catalonia, IQAC-CSIC, Jordi Girona 18-26, 08034, Barcelona, Spain.

E-mail: yolanda.perez@iqac.csic.es.

b. Department of Biological Chemistry, Institute for advanced Chemistry of Catalonia, IQAC-CSIC, Jordi Girona 18-26, 08034, Barcelona, Spain.

E-mail: ignacio.alfonso@iqac.csic.es.

Table of contents:

Materials and methods **S3**

Fluorescence spectroscopy experiments **S4**

Titration of poltE$_4$Y with different cages **S4**

Titration of polyK$_4$Y with different cages **S6**

Titration of polyE$_6$K$_3$Y with different cages **S9**

NMR characterization of pseudopeptidic cages **S12**

NMR characterization of commercial polypeptides **S14**

Purification of commercial polypeptides **S17**

NMR characterization of the purified fractions of the polypeptides **S19**

Studies of cages binding to polypeptides by NMR **S34**

Molecular modeling of supramolecular complexes **S42**

References **S46**

Materials and methods

Synthesis of pseudopeptidic cages

Reagents and solvents were purchased from commercial suppliers (Sigma-Aldrich, Fluka or Merck) and were used without further purification. Compounds CyOrn, CyLys, CyHis, CyAsp and CyGlu were synthetized as previously described.[1,2] Preparative reverse phase purifications were performed on a Isolera Biotage instrument (KP-C18-HS, CH_3CN and water with 0.1% TFA). Analytical RP-HPLC was performed with a Hewlett Packard Series 1100 (UV detector 1315A) modular system using a reverse-phase Kromasil 100 C8 (15 x 0.46 cm, 5 μm) column. CH_3CNH_2O mixtures containing 0.1% TFA at 1 ml/min were used as mobile phase and monitoring wavelengths were set at 220, 254 and 280 nm.

Molecular modeling.

All the molecular models were performed with Macromodel as in Maestro version 13.6.121, MMshare Version 6.2.121, Release 2023-2, from Schrödinger. Conformational searches were carried out with the MCMM/LMCS sampling approach that combines Monte Carlo conformational searches with low mode sampling.[3-5] The generated geometries were optimized with the OPLSE4 force field[6] in implicit water,[7] and conformers within ca. 21 kJ/mol were stored for further comparison. Different initial geometries were tested and the local minima within 10.46 kJ/mol (2.5 kcal/mol) were superposed and analyzed within the Maestro package. The optimized geometry of each complex with the lowest potential energy was considered the global minima.

Fluorescence spectroscopy experiments

Titration of polyE$_4$Y with CyHis

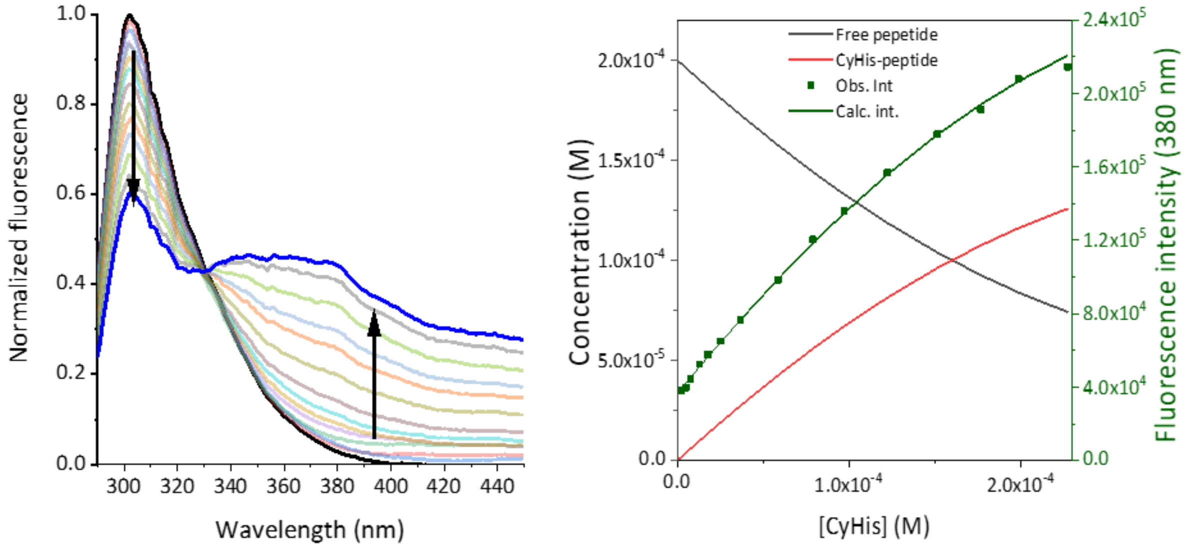

Figure S1. Normalized emission spectra of polyE$_4$Y (left, [polyE$_4$Y] = $2\cdot10^{-4}$ M, λexc = 276 nm) in buffered water (50 mM TRIS-HCl buffer) upon addition of CyHis. Right: species distribution and fluorescence intensity at 380 nm (observed and calculated) for the titration with a fitting model for the formation of a 1 : 1 complex using HypSpec. Log β = 4.22 ± 0.04, K_d = 60 ± 6 μM

Titration of polyE$_4$Y with CyAsp

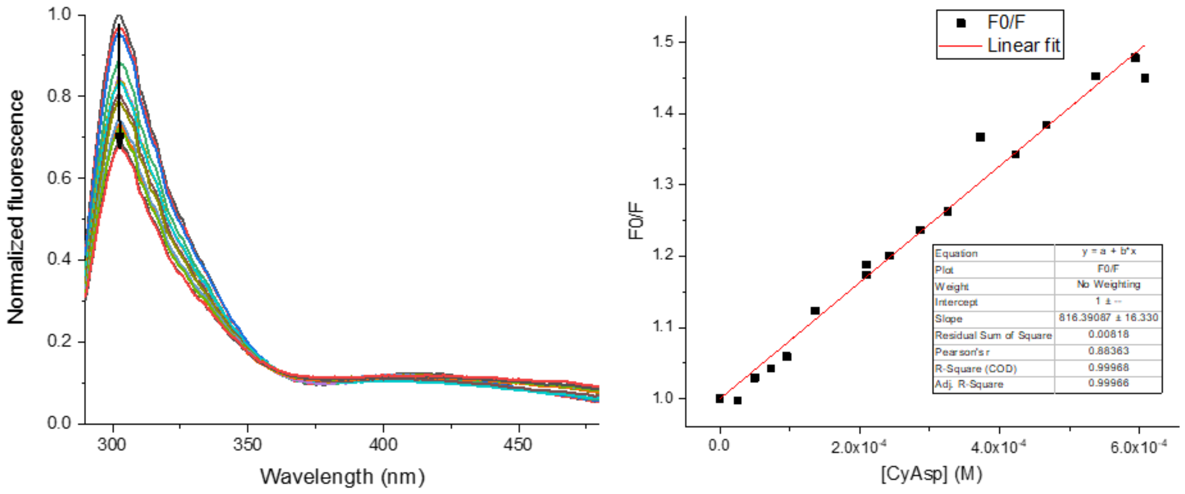

Figure S2. Normalized emission spectra of polyE$_4$Y (left, [polyE$_4$Y] = $2\cdot10^{-4}$ M, λexc = 276 nm) in buffered water (50 mM TRIS-HCl buffer) in absence and in presence of different amounts of CyAsp. Stern-Volmer plot (right) of the titration of polyE$_4$Y with CyAsp at 302 nm and linear fit of the data rendering K_{sv} = 816 ± 16 M^{-1}.

Titration of polyE$_4$Y with CyGlu

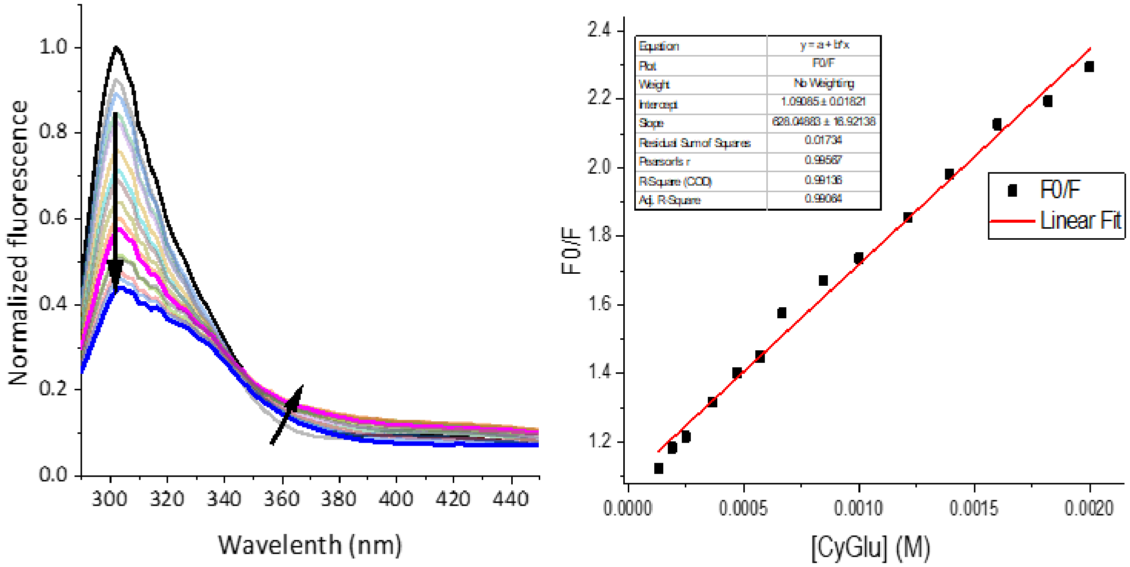

Figure S3. Normalized emission spectra (left, [polyE$_4$Y] = 2·10^{-4} M, λexc = 276 nm) of polyE$_4$Y in buffered water (50 mM TRIS-HCl buffer) upon addition of CyGlu. Stern-Volmer plot (right) of the titration and linear fit of the data rendering K_{sv} = 628 ± 17 M^{-1}.

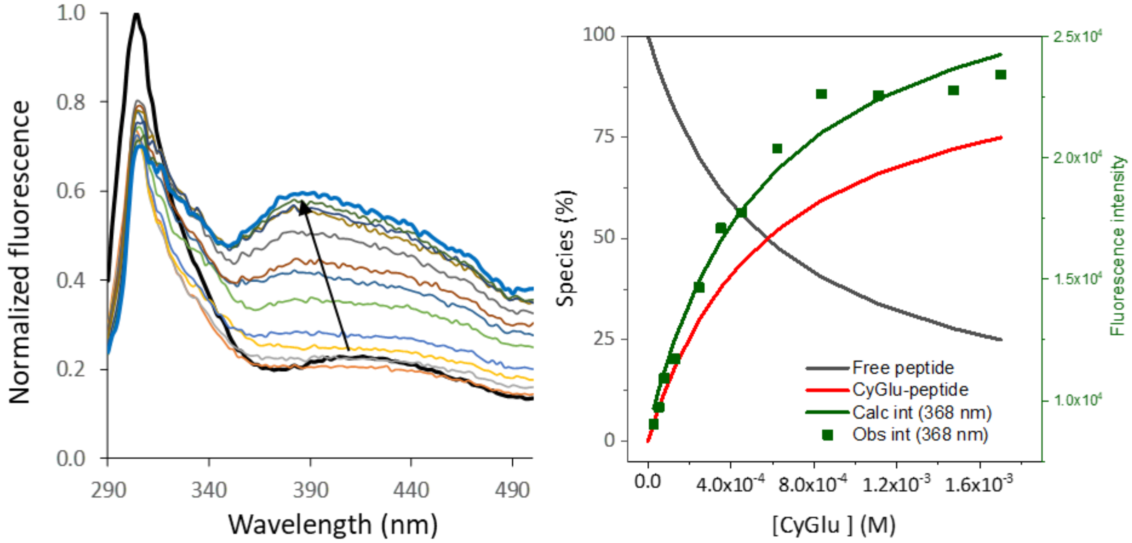

Figure S4. Normalized emission spectra (left, [polyE$_4$Y] = 2·10^{-5} M, λexc = 276 nm) of polyE$_4$Y in buffered water (50 mM TRIS-HCl buffer) upon addition of CyGlu. Right: fitting (HypSpec) to a 1 : 1 model of the titration data and species distribution. Log β = 2.76 ± 0.03, K_d = 1738 ± 84 μM.

Titration of polyK_4Y with CyOrn

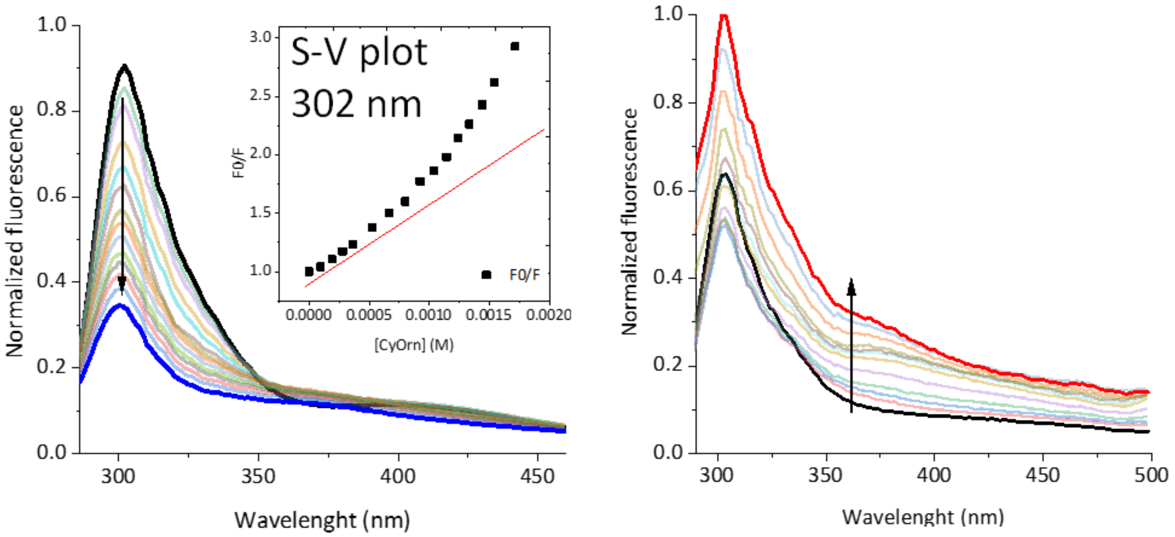

Figure S5. Normalized emission spectra of polyK_4Y (λexc = 276 nm) in buffered water (50 mM TRIS-HCl buffer) upon addition of CyOrn. Left: [polyK_4Y] = $2{\cdot}10^{-4}$M, the inset shows the Stern-Volmer plot at 302 nm. Right: [polyK_4Y] = $2{\cdot}10^{-5}$ M. No reliable fitting was possible in any of the cases.

Titration of polyK_4Y with CyLys

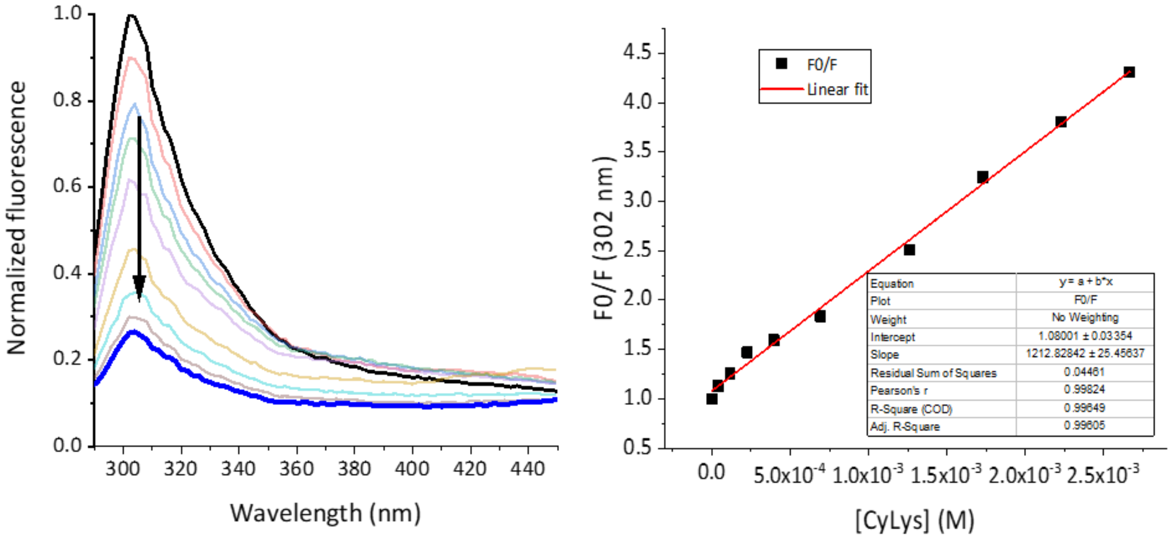

Figure S6. Normalized emission spectra of polyK_4Y (left, [polyK_4Y] = $2{\cdot}10^{-5}$ M, λexc = 276 nm) in buffered water (50 mM TRIS-HCl buffer) upon addition of CyLys; and Stern-Volmer plot at 302 nm (right) rendering K_{SV} = 1212 ± 25 M^{-1}.

Titration of polyK$_4$Y with CyHis

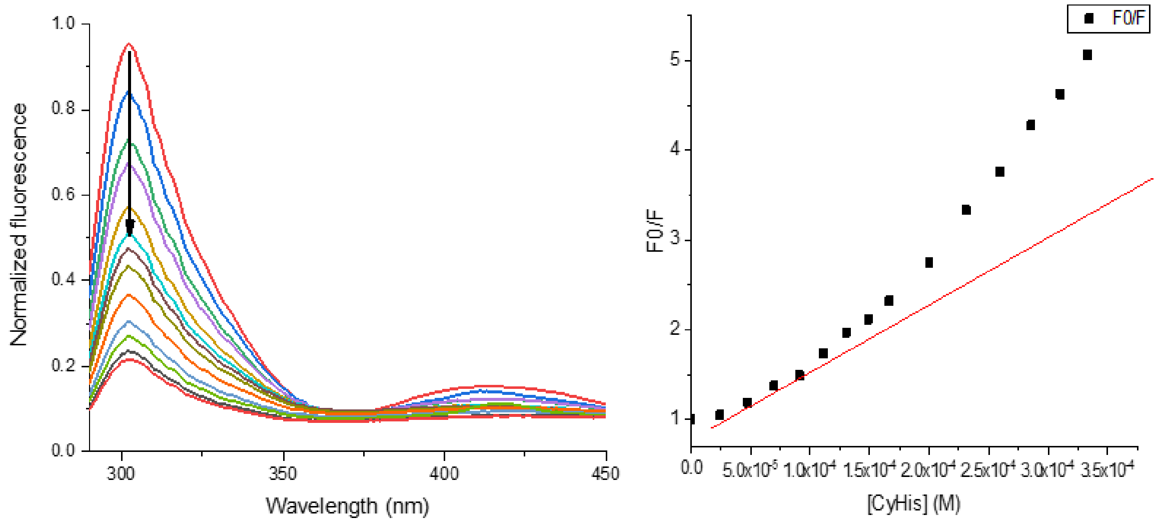

Figure S7. Normalized emission spectra of polyK$_4$Y (left, [polyK$_4$Y] = 2·10^{-4} M, λexc = 276 nm) in buffered water (50 mM TRIS-HCl buffer) upon addition of CyHis; and Stern-Volmer plot (right) at 301 nm (red line shows a linear response). Since the quenching showed deviation from linearity, we repeated the titration with a more diluted sample.

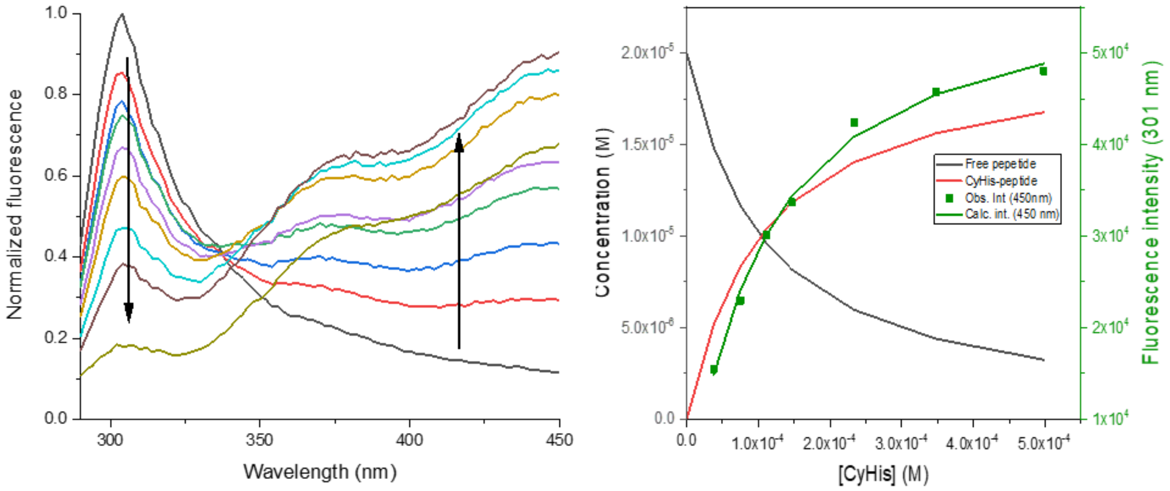

Figure S8. Normalized emission spectra of polyK$_4$Y (left, [polyK$_4$Y] = 2·10^{-5} M, λexc = 276 nm) in buffered water (50 mM TRIS-HCl buffer) upon addition of CyHis. Species distribution and fluorescence intensity at 450 nm (observed and calculated, right) of the titration with a fitting model for the formation of 1 : 1 complex using HypSpec. Log β = 4.08 ± 0.01, K_d = 93 ± 2 µM.

Titration of polyK$_4$Y with CyAsp

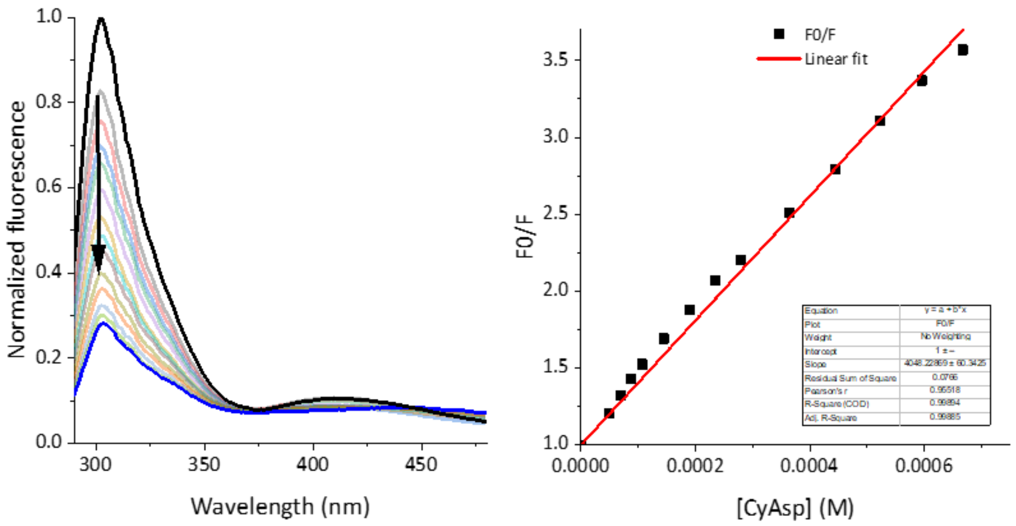

Figure S9. Normalized emission spectra of polyK$_4$Y (left, [polyK$_4$Y] = $2{\cdot}10^{-4}$ M, λexc = 276 nm) in buffered water (50 mM TRIS-HCl buffer) upon addition of CyAsp. Right: Stern-Volmer plot of the data rendering a K_{sv} = 4048 ± 60 M^{-1}.

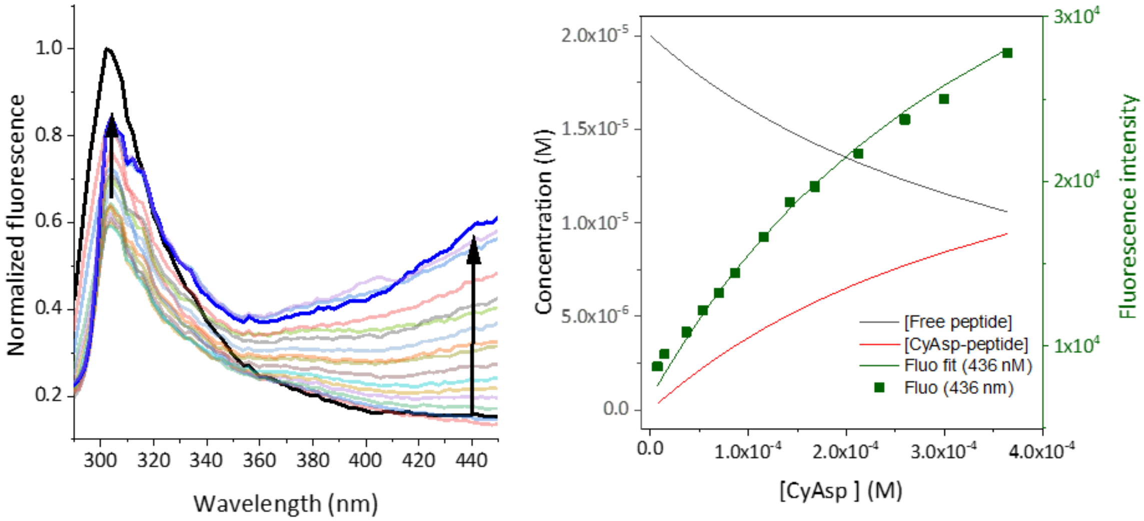

Figure S10. Normalized emission spectra of polyK$_4$Y (left, [polyK$_4$Y] = $2{\cdot}10^{-5}$ M, λexc = 276 nm) in buffered water (50 mM TRIS-HCl buffer) upon addition of CyAsp. Right: fitting of the data to a 1 : 1 complex using HypSpec. Log β = 3.40 ± 0.01, K_d = 398 ± 10 µM

Titration of polyK$_4$Y with CyGlu

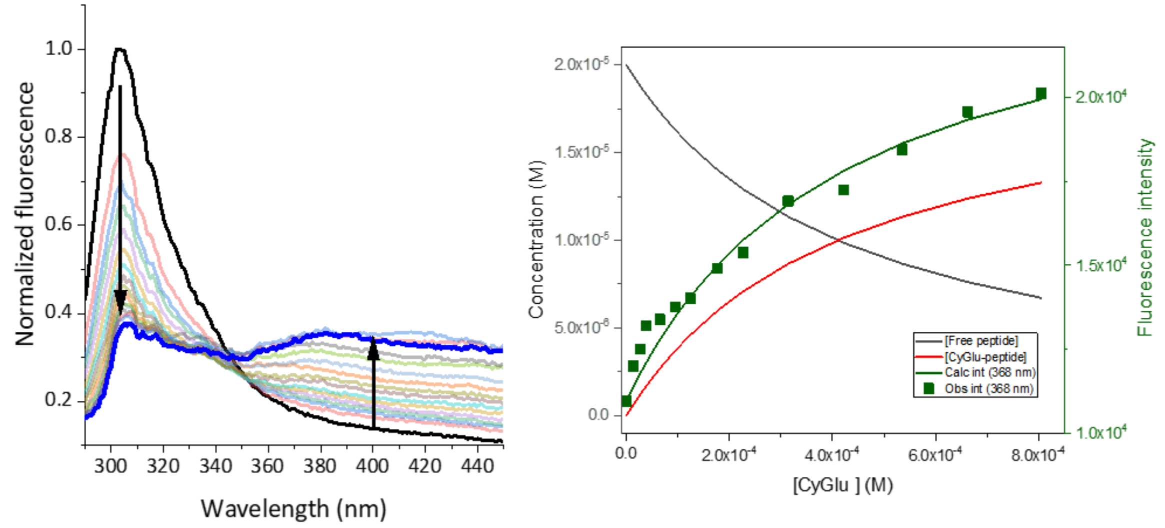

Figure S11. Normalized emission spectra of polyK$_4$Y (left, [polyK$_4$Y] = 2·10^{-5} M, λexc = 276 nm) in buffered water (50 mM TRIS-HCl buffer) upon addition of CyGlu. Right: fitting of the data at 368 nm to a 1 : 1 complex using HypSpec. Log β = 3.398 ± 0.009, K_d = 400 ± 10 µM.

Titration of polyE$_6$K$_3$Y with CyOrn

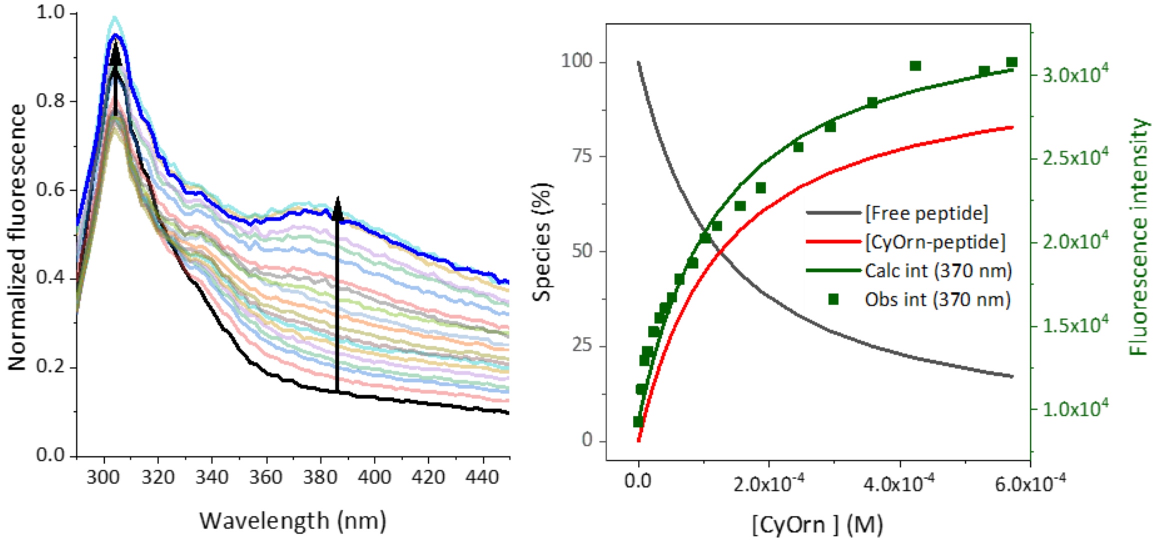

Figure S12. Normalized emission spectra of polyE$_6$K$_3$Y (left, [polyE$_6$K$_3$Y] = 2·10^{-5} M, λexc = 276 nm) in buffered water (50 mM TRIS-HCl buffer) upon addition of CyOrn. Right: fitting of the data to a 1 : 1 complex using HypSpec with species distribution, for fluorescence intensity at 370 nm (observed and calculated). Log β = 3.94 ± 0.007, K_d = 114 ± 2 µM.

Titration of polyE_6K_3Y with CyLys

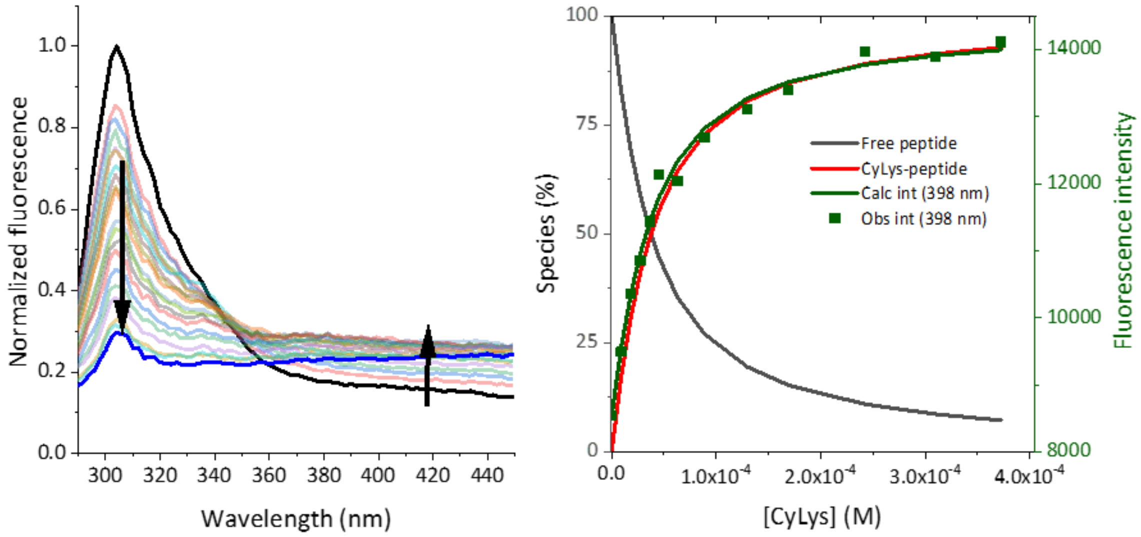

Figure S13. Normalized emission spectra of polyE_6K_3Y (left, [polyE_6K_3Y] = $2\cdot10^{-5}$ M, λexc = 276 nm) in buffered water (50 mM TRIS-HCl buffer) upon addition of CyLys. Right: fitting of the data to a 1 : 1 complex using HypSpec with species distribution, for fluorescence intensity at 398 nm (observed and calculated). Log β = 4.56 ± 0.01, K_d = 27.5± 0.6 µM.

Titration of polyE_6K_3Y with CyHis

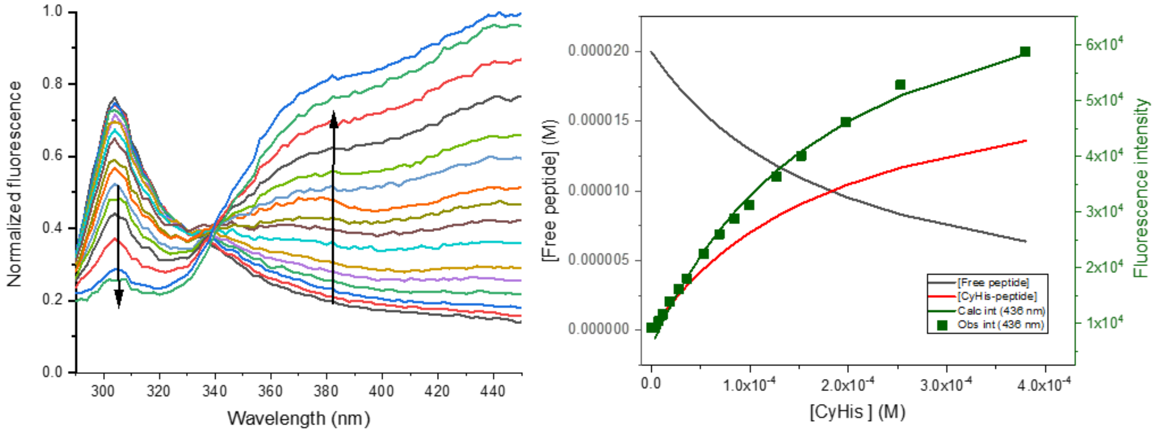

Figure S14. Normalized emission spectra of polyE_6K_3Y (left, [polyE_6K_3Y] = $2\cdot10^{-5}$ M, λexc = 276 nm) in buffered water (50 mM TRIS-HCl buffer) upon addition of CyHis. Right: fitting of the data for fluorescence intensity at 436 nm (observed and calculated) to a 1 : 1 complex using HypSpec with species distribution. Log β = 3.710 ± 0.005, K_d = 194 ± 3 µM.

Titration of polyE$_6$K$_3$Y with CyAsp

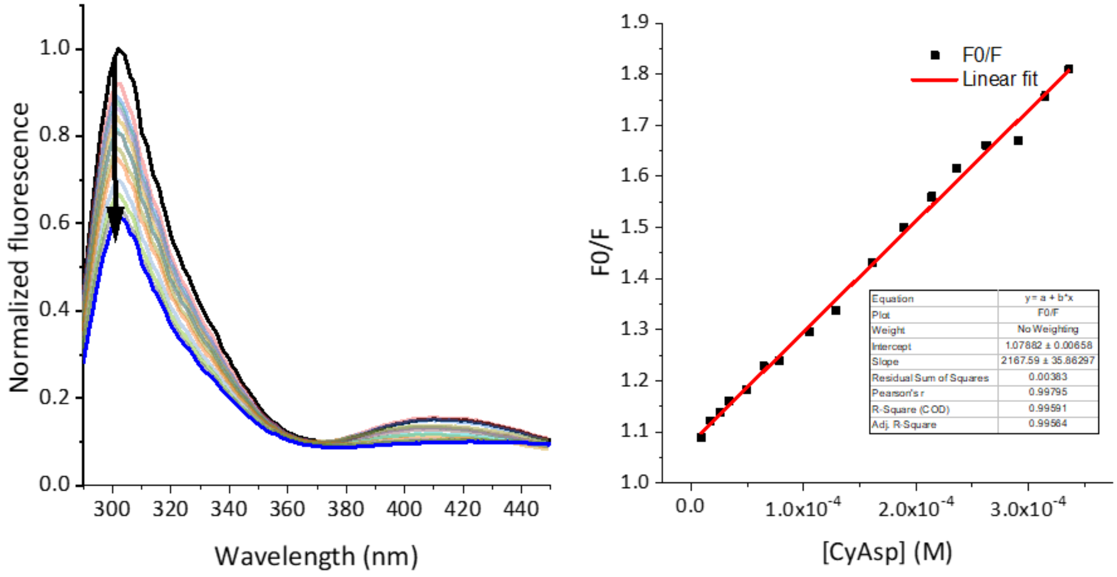

Figure S15. Normalized emission spectra of polyE$_6$K$_3$Y (left, [polyE$_6$K$_3$Y] = $2 \cdot 10^{-4}$ M, λexc = 276 nm) in buffered water (50 mM TRIS-HCl buffer) upon addition of CyAsp. Right: Stern-Volmer plot of the titration and linear fit of the data rendering a K_{SV} = 2168 ± 36 M^{-1}.

Titration of polyE$_6$K$_3$Y with CyGlu

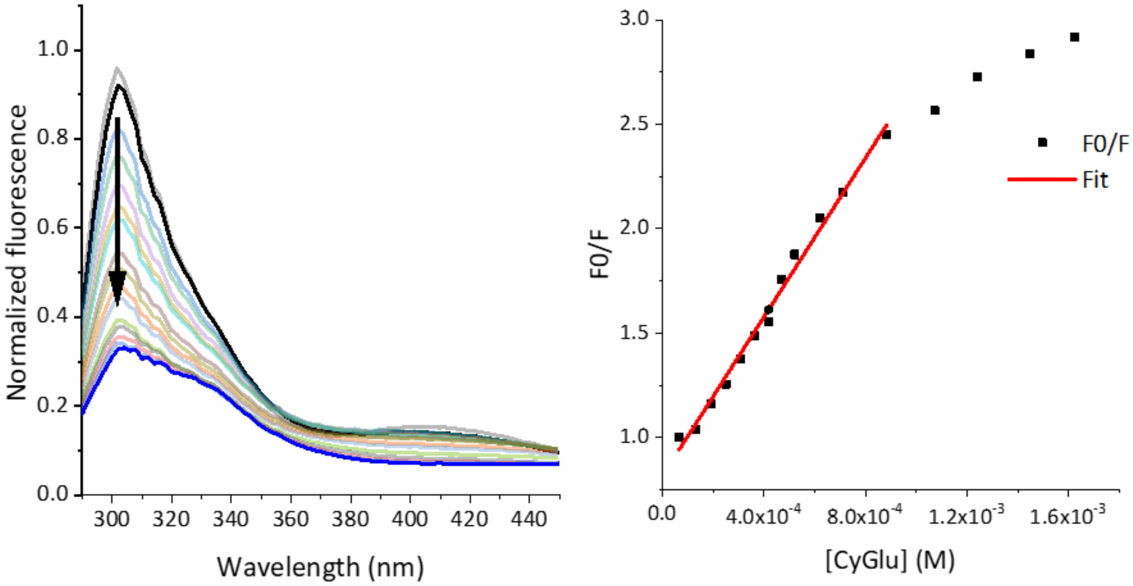

Figure S16. Normalized emission spectra of polyE$_6$K$_3$Y (left, [polyE$_6$K$_3$Y] = $2 \cdot 10^{-4}$ M, λexc = 276 nm) in buffered water (50 mM TRIS-HCl buffer) upon addition of CyGlu. Right: Stern-Volmer plot at 302 nm shows a linear trend up to 1 mM CyGlu (fitting in red), rendering a K_{sv} = 1902 ± 56 M^{-1}.

NMR characterization of pseudopeptidic cages

Table S1. Measured diffusion constants for pseudopeptidic cages. Samples: 1 mM CyLys in D_2O, 15 mM HEPES-d_{18}, 50 mM NaCl, pH=7.0; 1 mM CyOrn in D_2O, 50 mM TRIS-d_{11}, pH=4.7; 1 mM in D_2O, 15 mM HEPES-d_{18}, 50 mM NaCl, pH=7.0; 0.4 mM CyHis in D_2O, 15 mM HEPES-d_{18}, 50 mM NaCl, pH=7.0. The hydrodynamic radius, r_H, was calculated from the obtained diffusion constant. The results are comparable to the structurally similar **4a** cage previously measured.[8]

Sample	D (10^{-10} m^2/s)	r_H (10^{-10} m)[b]	r_H (10^{-10} m)[c]
CyLys in D_2O	2.03 ± 0.011	9.57	10.95
CyOrn in D_2O	2.15 ± 0.018	9.04	10.42
CyAsp in D_2O	2.19 ± 0.010	8.87	10.26
CyHis in D_2O	2.30 ± 0.021	8.45	9.83
4a in $CDCl_3$[a]	4.86 ± 0.15	8.26	10.79

[a]Structurally similar cage from previous work.[8]
[b]Calculated with the Stokes-Einstein equation for a sphere.
[c]Calculated with the SEGWE model proposed by Evans et al.[9]

Table S2. Comparison of measured T_1 values for a sample of 1 mM CyLys in D_2O, 15 mM HEPES-d_{18}, pH=7.0 using either Bruker library's standard inversion-recovery sequence (t1ir) or one with presaturation during relaxation delay (d1) for the suppression of the water signal (t1irpr). Both sequences provide comparable results for all protons with the exception of the alpha proton, which is the closest to the resonance of water.

Sequence	T_1 H_{ar} (s)	T_1 H_a (s)	T_1 H_{bz} (s)	T_1 H_ε (s)	T_1 H_b (s)
t1ir	$1.22 \pm 4.5 \cdot 10^{-4}$	$0.94 \pm 1.7 \cdot 10^{-4}$	$0.61 \pm 2.2 \cdot 10^{-4}$	$0.86 \pm 1.2 \cdot 10^{-4}$	$0.53 \pm 2.3 \cdot 10^{-4}$
t1irpr	$1.24 \pm 1.9 \cdot 10^{-4}$	$1.10 \pm 1.5 \cdot 10^{-4}$	$0.64 \pm 8.9 \cdot 10^{-4}$	$0.89 \pm 4.9 \cdot 10^{-4}$	$0.54 \pm 8.5 \cdot 10^{-4}$

Table S3. Comparison of measured T_2 values for samples of 1 mM CyOrn in D_2O, 50 mM tris-d_{11}, pH=4.5-4.9 using both standard CPMG and CPMG-PROJECT sequences with (cpmgpr1d, cpmg_esgp2d, project_cpmgpr1d) and without (project_cpmg) suppression of the water signal. For the standard CPMG sequences, good fitting of the data was obtained only for the aromatic and benzylic proton signals (black). J-modulation distorted the phase of the rest of the signals, which resulted in poor fitting (red). Good fitting was obtained for all of the signals with the CPMG-PROJECT sequences, which suppress J-modulation.

Sample	pH	Sequence	T_2 H_{ar} (s)	T_2 H_a (s)	T_2 H_α (s)	T_2 H_d (s)	T_2 H_δ (s)
1	4.9	cpmg_esgp2d	$0.22 \pm 1.1 \cdot 10^{-5}$	$0.06 \pm 5.7 \cdot 10^{-6}$	$0.08 \pm 4.5 \cdot 10^{-6}$	$0.09 \pm 2.9 \cdot 10^{-6}$	$0.11 \pm 3.6 \cdot 10^{-6}$
1	4.9	cpmgpr1d	$0.21 \pm 1.6 \cdot 10^{-5}$	$0.03 \pm 5.0 \cdot 10^{-6}$	$0.03 \pm 4.2 \cdot 10^{-6}$	$0.09 \pm 5.3 \cdot 10^{-6}$	$0.03 \pm 2.7 \cdot 10^{-6}$
2[a]	4.7	project_cpmg	$0.21 \pm 3.0 \cdot 10^{-5}$	$0.22 \pm 2.3 \cdot 10^{-5}$	$0.16 \pm 1.7 \cdot 10^{-5}$	$0.08 \pm 1.2 \cdot 10^{-5}$	$0.28 \pm 1.8 \cdot 10^{-5}$
3[a]	4.5	project_cpmgpr1d	$0.18 \pm 6.7 \cdot 10^{-6}$	$0.24 \pm 8.1 \cdot 10^{-6}$	$0.16 \pm 5.3 \cdot 10^{-6}$	$0.08 \pm 4.5 \cdot 10^{-6}$	$0.26 \pm 5.3 \cdot 10^{-6}$

[a]Each spectrum was phased individually for a better adjustment

NMR characterization of commercial polypeptides (prior to purification by chromatography)

Table S4. Different concentration/pH/salt concentration effects on T_1 values for the same batch (samples 1-4) and between batches (samples 1-4 and samples 5-6) of polyE$_4$Y. A comparison between different batches is shown in blue and red (not exactly the same polymer concentrations, but same added salt and pH conditions). The buffer for all the samples was 15 mM HEPES-d$_{18}$.

Sample	Batch	Concentration (mM)	pH	NaCl concentration (mM)	T_1 $H_{ar(ortho)(Y)}$ (s)	T_1 $H_{ar(meta)(Y)}$ (s)	T_1 $H_{\gamma(E)}$ (s)	T_1 $H_{\beta(E)}$ (s)
1	A	1.0	7.0	-	1.63 ± $1.4 \cdot 10^{-3}$	2.25 ± $1.7 \cdot 10^{-3}$	0.48 ± $1.6 \cdot 10^{-4}$	0.48 ± $1.6 \cdot 10^{-4}$
2	A	1.0	7.0	50	1.11 ± $6.9 \cdot 10^{-4}$	1.36 ± $7.5 \cdot 10^{-4}$	0.33 ± $8.8 \cdot 10^{-5}$	0.33 ± $8.8 \cdot 10^{-5}$
3	A	0.19	7.0	50	0.94 ± $1.5 \cdot 10^{-3}$	1.11 ± $1.5 \cdot 10^{-3}$	0.31 ± $2.7 \cdot 10^{-4}$	0.29 ± $2.2 \cdot 10^{-4}$
4	A	1.0	12.1	50	1.84 ± $8.7 \cdot 10^{-15}$	2.84 ± $1.8 \cdot 10^{-3}$	0.56 ± $1.2 \cdot 10^{-4}$	0.52 ± $1.2 \cdot 10^{-4}$
5	B	2.0	7.0	50	1.65 ± $8.7 \cdot 10^{-15}$	2.43 ± $1.0 \cdot 10^{-3}$	0.49 ± $8.1 \cdot 10^{-5}$	0.48 ± $8.6 \cdot 10^{-5}$
6	B	0.65	7.0	50	1.61 ± $8.7 \cdot 10^{-15}$	2.33 ± $2.5 \cdot 10^{-3}$	0.46 ± $1.8 \cdot 10^{-4}$	0.46 ± $2.3 \cdot 10^{-4}$

Table S5. Different concentration/pH/salt concentration effects on T_2 values for the same batch (samples 1-4) and between batches (samples 1-4 and samples 5-6) of polyE$_4$Y. A comparison between different batches is shown in blue and red (not exactly the same polymer concentrations, but same added salt and pH conditions). The buffer for all the samples was 15 mM HEPES-d$_{18}$.

Sample	Batch	Concentration (mM)	pH	NaCl concentration (mM)	T_2 $H_{ar(ortho)(Y)}$ (s)	T_2 $H_{ar(meta)(Y)}$ (s)	T_2 $H_{\gamma(E)}$ (s)	T_2 $H_{\beta(E)}$ (s)
1	A	1	7.0	-	0.33 ± $8.8{\cdot}10^{-5}$	0.38 ± $9.6{\cdot}10^{-5}$	0.14 ± $1.1{\cdot}10^{-5}$	0.14 ± $1.6{\cdot}10^{-5}$
2	A	1	7.0	50	0.28 ± $1.2{\cdot}10^{-4}$	0.31 ± $1.2{\cdot}10^{-4}$	0.12 ± $1.5{\cdot}10^{-5}$	0.12 ± $1.7{\cdot}10^{-5}$
3	A	0.19	7.0	50	0.26 ± $3.5{\cdot}10^{-4}$	0.27 ± $3.4{\cdot}10^{-4}$	0.11 ± $4.4{\cdot}10^{-5}$	0.11 ± $4.6{\cdot}10^{-5}$
4	A	1	12.1	50	0.45 ± $2.3{\cdot}10^{-4}$	0.54 ± $2.2{\cdot}10^{-4}$	0.18 ± $2.3{\cdot}10^{-5}$	0.17 ± $2.6{\cdot}10^{-5}$
5	B	2	7.0	50	0.39 ± $7.7{\cdot}10^{-5}$	0.42 ± $7.5{\cdot}10^{-5}$	0.16 ± $9.1{\cdot}10^{-6}$	0.15 ± $9.9{\cdot}10^{-6}$
6	B	0.65	7.0	50	0.38 ± $1.6{\cdot}10^{-4}$	0.40 ± $1.5{\cdot}10^{-4}$	0.14 ± $2.0{\cdot}10^{-5}$	0.14 ± $2.1{\cdot}10^{-5}$

Table S6. Different concentration/pH/salt effects on D values for the same batch (samples 1-4 and 7-9) and between batches (samples 1-4, 7-9 and samples 5-6) of polyE$_4$Y. A comparison between different batches is shown in blue and red (not exactly the same polymer concentrations, but same added salt and pH conditions). The buffer for all the samples was 15 mM HEPES-d$_{18}$.

Sample	Batch	Concentration (mM)	pH	NaCl concentration (mM)	D $H_{ar(ortho)(Y)}$ (10^{-11} m^2/s)	D $H_{ar(meta)(Y)}$ (10^{-11} m^2/s)	D $H_{\gamma(E)}$ (10^{-11} m^2/s)	D $H_{\beta(E)}$ (10^{-11} m^2/s)
1	A	1.0	7.0	-	6.67 ± $1.5\cdot10^{-3}$	6.57 ± $1.4\cdot10^{-3}$	6.74 ± $5.7\cdot10^{-4}$	6.71 ± $6.3\cdot10^{-4}$
2	A	1.0	7.0	50	8.12 ± $2.8\cdot10^{-3}$	8.07 ± $2.6\cdot10^{-3}$	8.19 ± $1.2\cdot10^{-3}$	8.10 ± $1.4\cdot10^{-3}$
3	A	0.2	7.0	50	7.93 ± $1.3\cdot10^{-2}$	8.34 ± $1.2\cdot10^{-2}$	7.99 ± $6.4\cdot10^{-3}$	8.48 ± $7.4\cdot10^{-3}$
4	A	1.0	12.1	50	7.69 ± $5.0\cdot10^{-3}$	7.72 ± $4.0\cdot10^{-3}$	7.61 ± $1.5\cdot10^{-3}$	7.77 ± $1.7\cdot10^{-3}$
5	B	2.0	7.0	50	7.79 ± $4.0\cdot10^{-3}$	7.84 ± $3.9\cdot10^{-3}$	7.68 ± $1.3\cdot10^{-3}$	7.81 ± $1.5\cdot10^{-3}$
6	B	0.65	7.0	50	8.01 ± $7.2\cdot10^{-3}$	7.95 ± $6.5\cdot10^{-3}$	7.94 ± $2.4\cdot10^{-3}$	7.98 ± $2.7\cdot10^{-3}$
7	A	0.2	7.0	-	7.09 ± $6.8\cdot10^{-3}$	7.05 ± $6.4\cdot10^{-3}$	7.31 ± $3.2\cdot10^{-3}$	7.39 ± $3.6\cdot10^{-3}$
8	A	1.0	12.1	-	7.34 ± $2.4\cdot10^{-3}$	7.28 ± $2.1\cdot10^{-3}$	7.31 ± $7.2\cdot10^{-4}$	7.28 ± $8.1\cdot10^{-4}$
9	A	0.33	12.1	-	7.62 ± $7.4\cdot10^{-3}$	7.65 ± $6.8\cdot10^{-3}$	7.67 ± $2.4\cdot10^{-3}$	7.86 ± $2.6\cdot10^{-3}$

Purification of commercial polypeptides

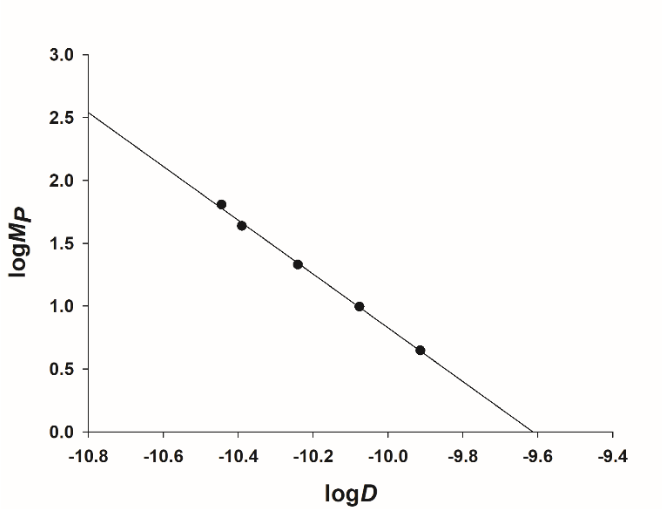

Sample	Average D (10^{-11} m^2/s)	M_p (kDa)[a]
Dextran Std 70k	3.59 ± 0.03	64.35
Dextran Std 50k	4.07 ± 0.04	43.50
Dextran Std 25k	5.74 ± 0.04	21.40
Dextran Std 12k	8.39 ± 0.02	9.89
Dextran Std 5k	12.20 ± 0.05	4.44
PSS 70k	3.70 ± 0.15	56.48[b]

[a]M_p by SEC provided by Sigma
[b]Calculated from the calibration

Figure S17. Double logarithmic plot of D against M_p. DOSY experiments were carried out at 298 K in D_2O using dilute solutions (1 mg/ml) of the dextran and PSS standards, using the BPPSTE NMR pulse sequence. M_p is the molecular weight of the highest peak in the determination of the average molecular weight of a polymer by SEC. As the accuracy of the M_p values calculated for the polypeptide fractions using dextran calibration remained unclear (this information comes from the manufacturer), we decided to measure the diffusion coefficient of a sample of Poly(4-styrenesulfonate acid) (PSS), a charged and linear aromatic polymer, more similar to tyrosine polyaminoacids. Very similar D values were obtained for the 70 kDa dextran standard ($3.59 \cdot 10^{-11}$ m^2/s) and for average M_w ~70,000 PSS ($3.70 \cdot 10^{-11}$ m^2/s).

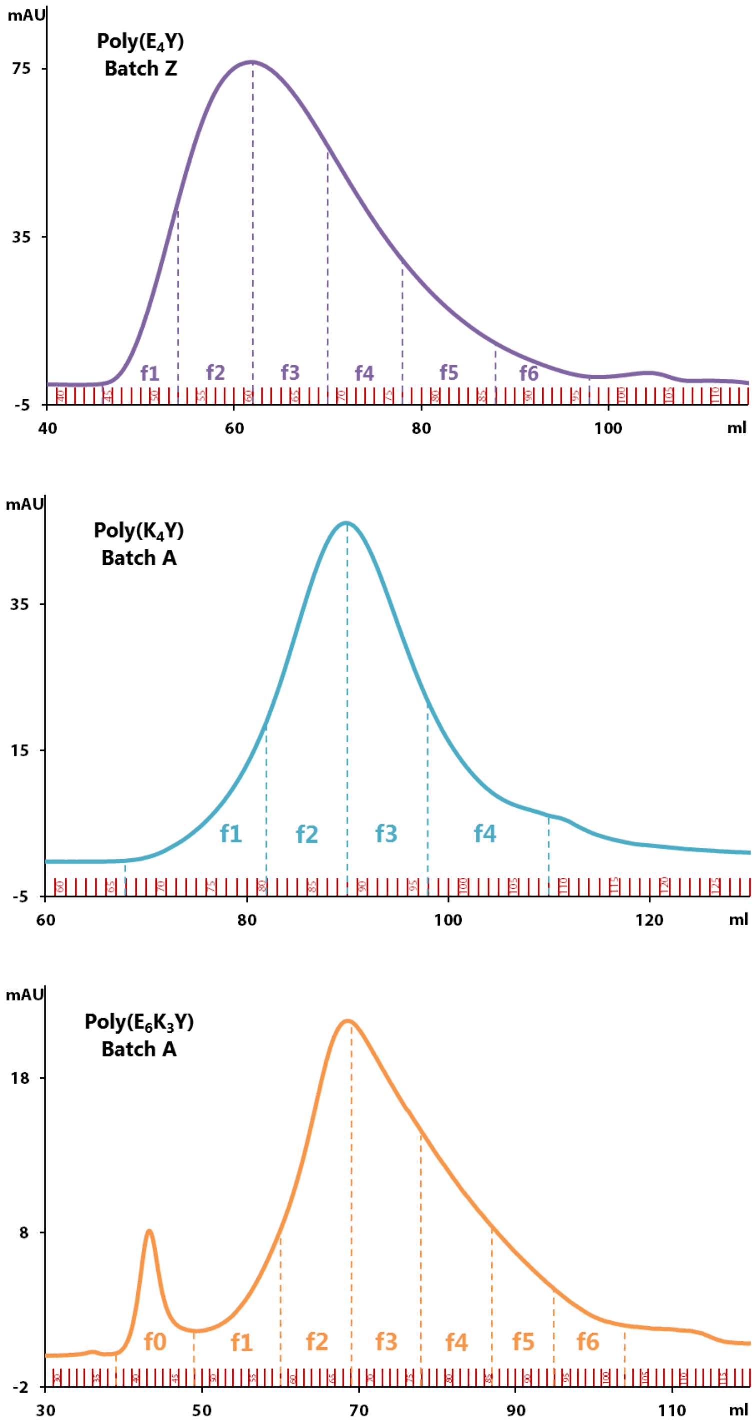

Figure S18. Size-exclusion chromatography profiles of three different commercial polypeptides: polyE_4Y Batch Z, polyK_4Y and polyE_6K_3Y (20-50 kDa, MW range provided by the supplier) with the fractions selected and collected for each polypeptide.

NMR characterization of the purified fractions of the polypeptides

Tables S7a-d. Measured diffusion coefficients for the four fractions obtained by SEC for batches B #1, B #2 and C of the 5-20 kDa polyE$_4$Y. All the samples were 0.6 mM polyE$_4$Y, 15 mM HEPES-d$_{18}$, 50 mM NaCl, pH=7.0.

**PolyE$_4$Y Batch B prior to SEC**			
**D $H_{ar(ortho)(Y)}$ (10^{-11} m^2/s)**	**D $H_{ar(meta)(Y)}$ (10^{-11} m^2/s)**	**D $H_{\gamma(E)}$ (10^{-11} m^2/s)**	**D $H_{\beta(E)}$ (10^{-11} m^2/s)**
8.01 ± 0.0072	7.95 ± 0.0065	7.94 ± 0.0024	7.98 ± 0.0027

**PolyE$_4$Y Batch B #1**				
**Fraction**	**D $H_{ar(ortho)(Y)}$ (10^{-11} m^2/s)**	**D $H_{ar(meta)(Y)}$ (10^{-11} m^2/s)**	**D $H_{\gamma(E)}$ (10^{-11} m^2/s)**	**D $H_{\beta(E)}$ (10^{-11} m^2/s)**
f1	5.41 ± 0.012	5.50 ± 0.010	5.68 ± 0.0052	5.47 ± 0.0054
f2	7.06 ± 0.015	7.28 ± 0.014	7.16 ± 0.0063	7.51 ± 0.0068
f3	10.20 ± 0.021	10.39 ± 0.021	10.50 ± 0.0085	10.67 ± 0.010
f4	15.48 ± 0.033	15.60 ± 0.033	16.69 ± 0.019	16.65 ± 0.021

**PolyE$_4$Y Batch B #2**				
**Fraction**	**D $H_{ar(ortho)(Y)}$ (10^{-11} m^2/s)**	**D $H_{ar(meta)(Y)}$ (10^{-11} m^2/s)**	**D $H_{\gamma(E)}$ (10^{-11} m^2/s)**	**D $H_{\beta(E)}$ (10^{-11} m^2/s)**
f1	5.65 ± 0.014	5.58 ± 0.013	5.50 ± 0.0066	5.50 ± 0.0065
f2	7.14 ± 0.015	7.16 ± 0.014	7.54 ± 0.0073	7.30 ± 0.0074
f3	10.11 ± 0.023	10.08 ± 0.018	10.60 ± 0.011	10.42 ± 0.011
f4	15.48 ± 0.033	14.97 ± 0.027	16.60 ± 0.022	16.11 ± 0.022

**PolyE$_4$Y Batch C**				
**Fraction**	**D $H_{ar(ortho)(Y)}$ (10^{-11} m^2/s)**	**D $H_{ar(meta)(Y)}$ (10^{-11} m^2/s)**	**D $H_{\gamma(E)}$ (10^{-11} m^2/s)**	**D $H_{\beta(E)}$ (10^{-11} m^2/s)**
f1	5.10 ± 0.012	5.15 ± 0.010	5.40 ± 0.0046	5.13 ± 0.0052
f2	7.02 ± 0.014	7.02 ± 0.013	7.20 ± 0.0061	7.11 ± 0.0062
f3	9.92 ± 0.020	9.96 ± 0.017	10.34 ± 0.0079	10.24 ± 0.086
f4	14.78 ± 0.030	14.82 ± 0.028	17.60 ± 0.015	15.79 ± 0.015

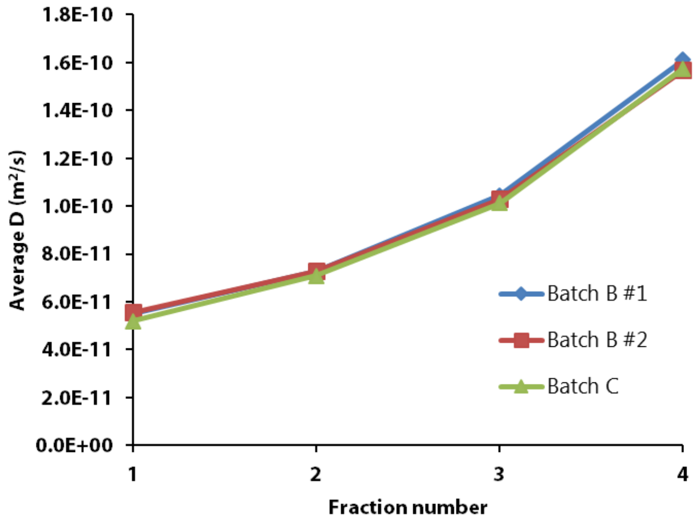

Figure S19. Representation of the D values (average of the different protons) tabulated in tables S7a-d. The measured D values were almost identical for each of the four fractions for the three runs, showing great reproducibility. Diffusion values were measured with Bruker's stebpgp1s19 sequence. 24 gradient steps were recorded where the gradient strength increased from 5% to 98% of the maximum strength. Δ was set to 150 ms and δ to 2.4 ms.

Table S8a-d. Measured T_1 for the four fractions obtained by SEC for batches B #1, B #2 and C of the 5-20 kDa polyE_4Y. All the samples were 0.6 mM polyE_4Y, 15 mM HEPES-d_{18}, 50 mM NaCl, pH=7.0. Non-selective T_1s were measured with the 2dt1irpr_cwvd sequence. 24 points were acquired with recovery delays ranging from 0.05 to 10 s.

**PolyE_4Y Batch B prior to SEC**			
**T_1 $H_{ar(ortho)(Y)}$ (s)**	**T_1 $H_{ar(meta)(Y)}$ (s)**	**T_1 $H_{\gamma(E)}$ (s)**	**T_1 $H_{\beta(E)}$ (s)**
$1.61 \pm 8.7 \cdot 10^{-15}$	$2.33 \pm 2.6 \cdot 10^{-3}$	$0.46 \pm 1.8 \cdot 10^{-4}$	$0.46 \pm 2.1 \cdot 10^{-4}$

**PolyE_4Y Batch B #1**				
**Fraction**	**T_1 $H_{ar(ortho)(Y)}$ (s)**	**T_1 $H_{ar(meta)(Y)}$ (s)**	**T_1 $H_{\gamma(E)}$ (s)**	**T_1 $H_{\beta(E)}$ (s)**
f1	$1.20 \pm 1.4 \cdot 10^{-3}$	$1.59 \pm 1.8 \cdot 10^{-14}$	$0.32 \pm 1.3 \cdot 10^{-4}$	$0.30 \pm 1.3 \cdot 10^{-4}$
f2	$1.36 \pm 3.3 \cdot 10^{-4}$	$1.82 \pm 2.9 \cdot 10^{-4}$	$0.34 \pm 1.7 \cdot 10^{-4}$	$0.35 \pm 1.7 \cdot 10^{-4}$
f3	$1.40 \pm 3.3 \cdot 10^{-4}$	$1.89 \pm 2.5 \cdot 10^{-4}$	$0.34 \pm 1.8 \cdot 10^{-4}$	$0.35 \pm 2.0 \cdot 10^{-4}$
f4	$1.27 \pm 3.1 \cdot 10^{-4}$	$1.72 \pm 2.7 \cdot 10^{-4}$	$0.28 \pm 1.5 \cdot 10^{-4}$	$0.30 \pm 1.9 \cdot 10^{-4}$

**PolyE_4Y Batch B #2**				
**Fraction**	**T_1 $H_{ar(ortho)(Y)}$ (s)**	**T_1 $H_{ar(meta)(Y)}$ (s)**	**T_1 $H_{\gamma(E)}$ (s)**	**T_1 $H_{\beta(E)}$ (s)**
f1	$1.19 \pm 1.6 \cdot 10^{-3}$	$1.52 \pm 1.8 \cdot 10^{-14}$	$0.26 \pm 1.2 \cdot 10^{-4}$	$0.29 \pm 1.4 \cdot 10^{-4}$
f2	$1.31 \pm 2.9 \cdot 10^{-4}$	$1.73 \pm 2.6 \cdot 10^{-4}$	$0.32 \pm 1.5 \cdot 10^{-4}$	$0.32 \pm 1.7 \cdot 10^{-4}$
f3	$1.29 \pm 3.7 \cdot 10^{-4}$	$1.71 \pm 3.2 \cdot 10^{-4}$	$0.30 \pm 1.7 \cdot 10^{-4}$	$0.31 \pm 1.8 \cdot 10^{-4}$
f4	$1.17 \pm 4.3 \cdot 10^{-4}$	$1.57 \pm 3.4 \cdot 10^{-4}$	$0.24 \pm 1.4 \cdot 10^{-4}$	$0.26 \pm 1.6 \cdot 10^{-4}$

**PolyE_4Y Batch C**				
**Fraction**	**T_1 $H_{ar(ortho)(Y)}$ (s)**	**T_1 $H_{ar(meta)(Y)}$ (s)**	**T_1 $H_{\gamma(E)}$ (s)**	**T_1 $H_{\beta(E)}$ (s)**
f1	$1.32 \pm 1.5 \cdot 10^{-3}$	$1.73 \pm 1.8 \cdot 10^{-14}$	$0.37 \pm 1.6 \cdot 10^{-4}$	$0.35 \pm 1.6 \cdot 10^{-4}$
f2	$1.43 \pm 2.8 \cdot 10^{-4}$	$1.93 \pm 2.4 \cdot 10^{-4}$	$0.39 \pm 6.2 \cdot 10^{-5}$	$0.38 \pm 6.5 \cdot 10^{-5}$
f3	$1.46 \pm 1.8 \cdot 10^{-14}$	$1.97 \pm 1.8 \cdot 10^{-14}$	$0.38 \pm 1.8 \cdot 10^{-4}$	$0.37 \pm 1.9 \cdot 10^{-4}$
f4	$1.38 \pm 2.7 \cdot 10^{-4}$	$1.92 \pm 2.1 \cdot 10^{-4}$	$0.34 \pm 3.4 \cdot 10^{-4}$	$0.36 \pm 8.2 \cdot 10^{-5}$

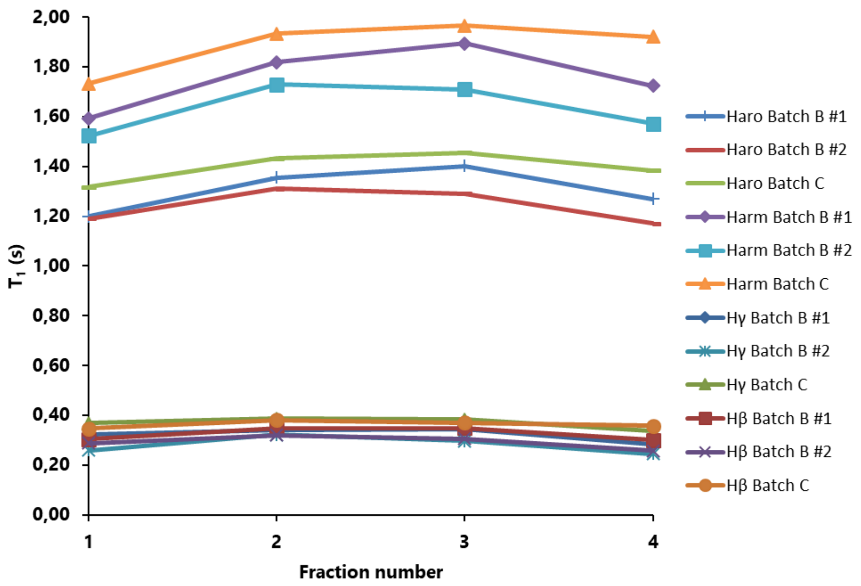

Figure S20. Representation of the T_1 values tabulated in tables S8a-d.

Table S9a-d. Measured T_2 for the four fractions obtained by SEC for batches B #1, B #2 and C of the 5-20 kDa polyE$_4$Y. All the samples were 0.6 mM polyE$_4$Y, 15 mM HEPES-d$_{18}$, 50 mM NaCl, pH=7.0. T_2s were measured with the projectcpmgpr1d sequence.

**PolyE$_4$Y Batch B prior to SEC**			
**T_2 $H_{ar(ortho)(Y)}$ (s)**	**T_2 $H_{ar(meta)(Y)}$ (s)**	**T_2 $H_{\gamma(E)}$ (s)**	**T_2 $H_{\beta(E)}$ (s)**
$0.38 \pm 1.6 \cdot 10^{-4}$	$0.40 \pm 1.5 \cdot 10^{-4}$	$0.14 \pm 2.0 \cdot 10^{-5}$	$0.14 \pm 2.1 \cdot 10^{-5}$

**PolyE$_4$Y Batch B #1**				
**Fraction**	**T_2 $H_{ar(ortho)(Y)}$ (s)**	**T_2 $H_{ar(meta)(Y)}$ (s)**	**T_2 $H_{\gamma(E)}$ (s)**	**T_2 $H_{\beta(E)}$ (s)**
f1	$0.26 \pm 2.0 \cdot 10^{-4}$	$0.27 \pm 1.8 \cdot 10^{-4}$	$0.08 \pm 1.7 \cdot 10^{-5}$	$0.07 \pm 1.7 \cdot 10^{-5}$
f2	$0.30 \pm 2.7 \cdot 10^{-4}$	$0.32 \pm 2.2 \cdot 10^{-4}$	$0.09 \pm 2.1 \cdot 10^{-5}$	$0.09 \pm 2.7 \cdot 10^{-5}$
f3	$0.34 \pm 2.3 \cdot 10^{-4}$	$0.37 \pm 2.3 \cdot 10^{-4}$	$0.10 \pm 2.6 \cdot 10^{-5}$	$0.10 \pm 2.9 \cdot 10^{-5}$
f4	$0.43 \pm 4.1 \cdot 10^{-4}$	$0.48 \pm 3.8 \cdot 10^{-4}$	$0.10 \pm 3.0 \cdot 10^{-5}$	$0.11 \pm 3.8 \cdot 10^{-5}$

**PolyE$_4$Y Batch B #2**				
**Fraction**	**T_1 $H_{ar(ortho)(Y)}$ (s)**	**T_1 $H_{ar(meta)(Y)}$ (s)**	**T_1 $H_{\gamma(E)}$ (s)**	**T_1 $H_{\beta(E)}$ (s)**
f1	$0.24 \pm 2.5 \cdot 10^{-4}$	$0.26 \pm 2.3 \cdot 10^{-4}$	$0.06 \pm 1.8 \cdot 10^{-5}$	$0.06 \pm 2.0 \cdot 10^{-5}$
f2	$0.28 \pm 2.3 \cdot 10^{-4}$	$0.30 \pm 2.1 \cdot 10^{-4}$	$0.08 \pm 2.1 \cdot 10^{-5}$	$0.08 \pm 2.2 \cdot 10^{-5}$
f3	$0.32 \pm 3.1 \cdot 10^{-4}$	$0.34 \pm 2.5 \cdot 10^{-4}$	$0.09 \pm 2.4 \cdot 10^{-5}$	$0.09 \pm 2.8 \cdot 10^{-5}$
f4	$0.38 \pm 3.3 \cdot 10^{-4}$	$0.40 \pm 2.7 \cdot 10^{-4}$	$0.09 \pm 2.7 \cdot 10^{-5}$	$0.09 \pm 3.0 \cdot 10^{-5}$

**PolyE$_4$Y Batch C**				
**Fraction**	**T_1 $H_{ar(ortho)(Y)}$ (s)**	**T_1 $H_{ar(meta)(Y)}$ (s)**	**T_1 $H_{\gamma(E)}$ (s)**	**T_1 $H_{\beta(E)}$ (s)**
f1 (1C)	$0.24 \pm 2.1 \cdot 10^{-4}$	$0.28 \pm 1.8 \cdot 10^{-4}$	$0.07 \pm 3.4 \cdot 10^{-5}$	$0.08 \pm 2.1 \cdot 10^{-5}$
f2 (2C)	$0.32 \pm 2.6 \cdot 10^{-4}$	$0.35 \pm 2.2 \cdot 10^{-4}$	$0.08 \pm 8.2 \cdot 10^{-5}$	$0.11 \pm 2.5 \cdot 10^{-5}$
f3 (3C)	$0.35 \pm 2.6 \cdot 10^{-4}$	$0.38 \pm 2.2 \cdot 10^{-4}$	$0.12 \pm 2.7 \cdot 10^{-5}$	$0.11 \pm 3.1 \cdot 10^{-5}$
f4 (4C)	$0.44 \pm 3.3 \cdot 10^{-4}$	$0.48 \pm 2.7 \cdot 10^{-4}$	$0.10 \pm 8.1 \cdot 10^{-5}$	$0.14 \pm 4.0 \cdot 10^{-5}$

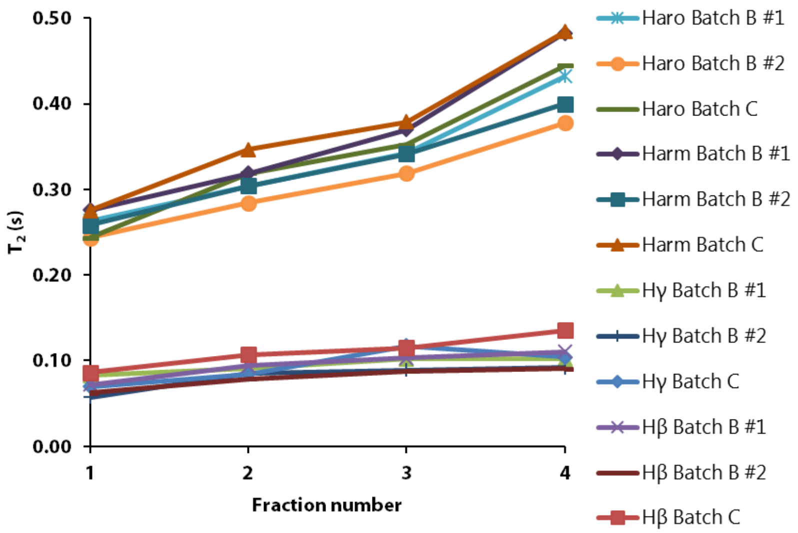

Figure S21. Representation of the T_1 values tabulated in tables S9a-d.

Tables S10a-c. Measured D, T_1 and T_2 values for the six fractions obtained by SEC for batch Z of the 20-50 kDa polyE$_4$Y. All the samples were 0.6 mM polyE$_4$Y, 15 mM HEPES-d$_{18}$, 50 mM NaCl, pH=7.0. Diffusion values were measured with Bruker's stebpgp1s19 sequence. 24 gradient steps were recorded where the gradient strength increased from 5% to 98% of the maximum strength. Δ was set to 150 ms and δ to 2.4 ms. Non-selective T_1s were measured with the 2dt1irpr_cwvd sequence. 24 points were acquired with recovery delays ranging from 0.05 to 10 s. T_2s were measured with the projectcpmgpr1d sequence

**PolyE$_4$Y Batch Z**				
**Fraction**	**D $H_{ar(ortho)(Y)}$ (10^{-11} m^2/s)**	**D $H_{ar(meta)(Y)}$ (10^{-11} m^2/s)**	**D $H_{\gamma(E)}$ (10^{-11} m^2/s)**	**D $H_{\beta(E)}$ (10^{-11} m^2/s)**
f1 (1Z)	2.23 ± 0.011	2.25 ± 0.0093	2.19 ± 0.020	2.22 ± 0.0051
f2 (2Z)	2.85 ± 0.0078	2.86 ± 0.0068	2.81 ± 0.050	2.83 ± 0.0036
f3 (3Z)	3.97 ± 0.0088	3.96 ± 0.0075	3.95 ± 0.22	3.94 ± 0.0038
f4 (4Z)	5.70 ± 0.011	5.70 ± 0.011	5.64 ± 0.18	5.71 ± 0.0054
f5 (5Z)	8.54 ± 0.016	8.56 ± 0.015	8.68 ± 0.094	8.69 ± 0.0077
f6 (6Z)	14.40 ± 0.086	14.11 ± 0.084	15.30 ± 0.13	15.65 ± 0.050

**PolyE$_4$Y Batch Z**				
**Fraction**	**T_1 $H_{ar(ortho)(Y)}$ (s)**	**T_1 $H_{ar(meta)(Y)}$ (s)**	**T_1 $H_{\gamma(E)}$ (s)**	**T_1 $H_{\beta(E)}$ (s)**
f1 (1Z)	$1.21 \pm 2.0\cdot10^{-3}$	$1.57 \pm 1.8\cdot10^{-14}$	$0.39 \pm 2.2\cdot10^{-4}$	$0.34 \pm 2.4\cdot10^{-4}$
f2 (2Z)	$1.36 \pm 1.8\cdot10^{-3}$	$1.84 \pm 1.8\cdot10^{-14}$	$0.35 \pm 3.1\cdot10^{-3}$	$0.38 \pm 6.4\cdot10^{-5}$
f3 (3Z)	$1.37 \pm 4.0\cdot10^{-4}$	$1.87 \pm 3.0\cdot10^{-4}$	$0.41 \pm 5.6\cdot10^{-5}$	$0.39 \pm 6.6\cdot10^{-5}$
f4 (4Z)	$1.40 \pm 3.2\cdot10^{-4}$	$1.88 \pm 3.0\cdot10^{-4}$	$0.40 \pm 5.7\cdot10^{-5}$	$0.39 \pm 6.8\cdot10^{-5}$
f5 (5Z)	$1.37 \pm 1.7\cdot10^{-3}$	$1.85 \pm 1.8\cdot10^{-14}$	$0.43 \pm 1.7\cdot10^{-4}$	$0.37 \pm 1.8\cdot10^{-4}$
f6 (6Z)	$1.36 \pm 5.1\cdot10^{-3}$	$1.88 \pm 1.8\cdot10^{-14}$	$0.46 \pm 5.7\cdot10^{-4}$	$0.35 \pm 4.7\cdot10^{-4}$

**PolyE$_4$Y Batch Z**				
**Fraction**	**T_2 $H_{ar(ortho)(Y)}$ (s)**	**T_2 $H_{ar(meta)(Y)}$ (s)**	**T_2 $H_{\gamma(E)}$ (s)**	**T_2 $H_{\beta(E)}$ (s)**
f1 (1Z)	$0.19 \pm 2.0\cdot10^{-4}$	$0.21 \pm 1.8\cdot10^{-4}$	$0.06 \pm 3.3\cdot10^{-5}$	$0.07 \pm 2.3\cdot10^{-5}$
f2 (2Z)	$0.23 \pm 1.9\cdot10^{-4}$	$0.25 \pm 1.6\cdot10^{-4}$	$0.07 \pm 4.0\cdot10^{-5}$	$0.08 \pm 2.1\cdot10^{-5}$
f3 (3Z)	$0.26 \pm 2.0\cdot10^{-4}$	$0.28 \pm 1.7\cdot10^{-4}$	$0.10 \pm 2.2\cdot10^{-5}$	$0.09 \pm 2.3\cdot10^{-5}$
f4 (4Z)	$0.28 \pm 2.2\cdot10^{-4}$	$0.30 \pm 1.9\cdot10^{-4}$	$0.10 \pm 2.3\cdot10^{-5}$	$0.10 \pm 2.6\cdot10^{-5}$
f5 (5Z)	$0.32 \pm 2.3\cdot10^{-4}$	$0.35 \pm 2.4\cdot10^{-4}$	$0.13 \pm 2.7\cdot10^{-5}$	$0.10 \pm 2.4\cdot10^{-5}$
f6 (6Z)	$0.41 \pm 9.4\cdot10^{-4}$	$0.44 \pm 8.6\cdot10^{-4}$	$0.17 \pm 9.9\cdot10^{-5}$	$0.12 \pm 8.7\cdot10^{-5}$

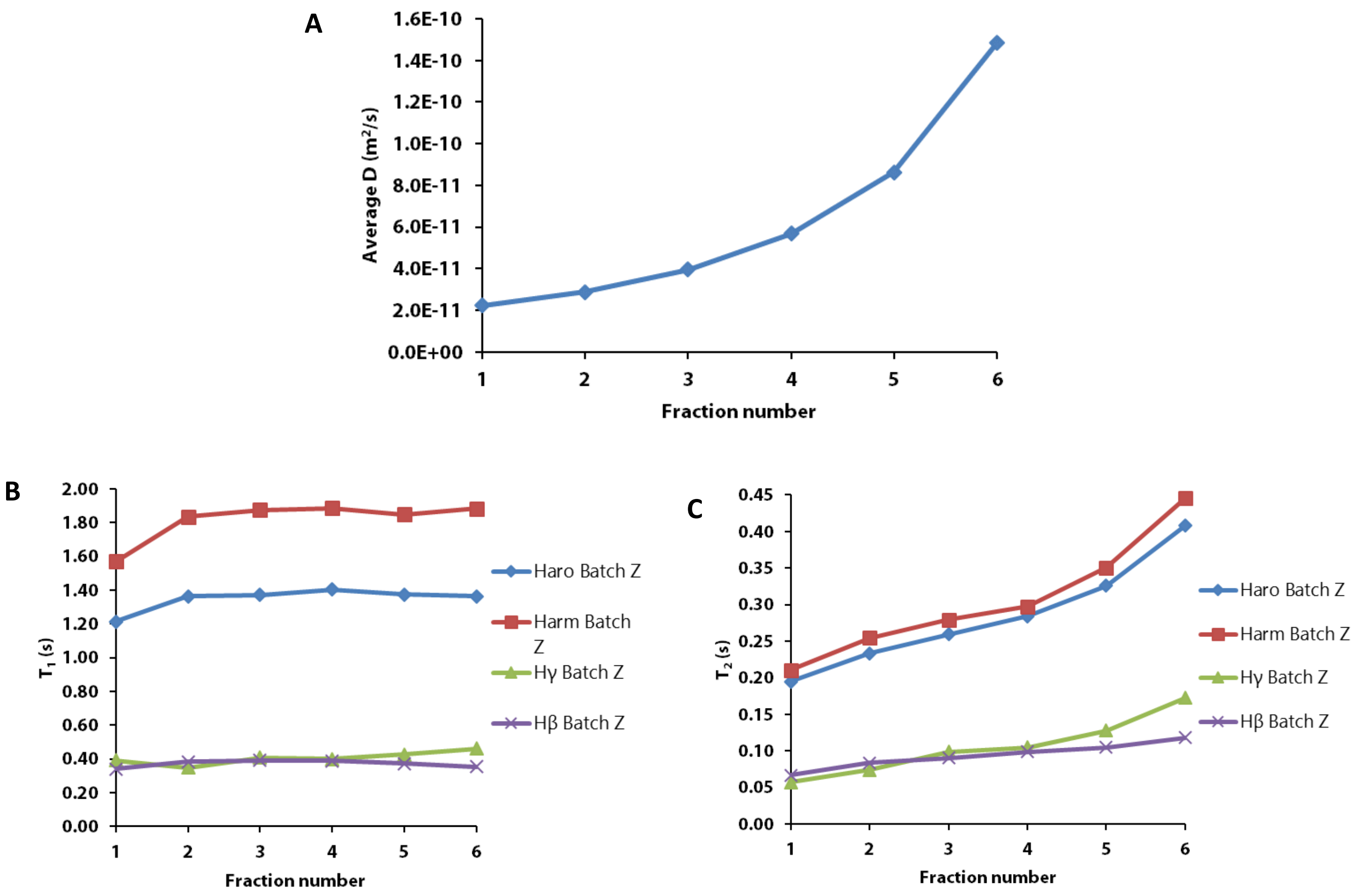

Figure S22. Representation of the D (average of the different protons), T_1 and T_2 values tabulated in tables S10a-c.

Table S11. Averaged diffusion coefficients for each of the fractions obtained by SEC for polyE$_4$Y batches C (5-20 kDa) and Z (20-50 kDa) and the M_p values calculated using the dextran calibration (Figure S2). The diffusion coefficient of poly(4-styrenesulfonate acid) (PSS), a charged, linear aromatic polymer more similar to tyrosine polyaminoacids was also measured and the corresponding M_p was calculated from the dextran calibration and is shown for comparison (in blue). Averaged coefficients were obtained averaging the four different protons measured of each sample. Fraction used for NMR interaction studies in green.

PolyE$_4$Y			
**Fraction/Batch**	**Average D (10^{-11} m^2/s)**	**M_p (kDa) [20-50 kDa Batch Z]**	**M_p (kDa) [20-5 kDa Batch C]**
1Z	2.22 ± 0.024	168.66	
2Z	2.86 ± 0.024	97.82	
PSS	3.70 ± 0.004	56.48	
3Z	3.95 ± 0.014	48.97	
1C	5.20 ± 0.140		27.26
4Z	5.69 ± 0.033	22.45	
2C	7.09 ± 0.086		14.11
5Z	8.62 ± 0.080	9.22	
3C	10.11 ± 0.210		6.54
6Z	14.86 ± 0.730	2.86	
4C	15.75 ± 1.320		2.53

Table S12. Averaged diffusion coefficients for each of the fractions obtained by SEC for polyK$_4$Y (20-50 kDa) and the M$_p$ values calculated using the dextran calibration (Figure S2). Averaged coefficients were obtained averaging the four different protons measured of each sample. Fraction used for NMR interaction studies in green.

PolyK$_4$Y		
Fraction/Batch	Average D (10^{-11} m^2/s)	M$_p$ (kDa) [20-50 kDa Batch A]
1A	5.25 ± 0.08	26.64
2A	6.39 ± 0.15	17.52
3A	8.03 ± 0.24	10.72
4A	9.99 ± 0.46	6.71

Table S13. Averaged diffusion coefficients for each of the fractions obtained by SEC for polyE$_6$K$_3$Y (20-50 kDa) and the M$_p$ values calculated using the dextran calibration (Figure S2). Averaged coefficients were obtained averaging the four different protons measured of each sample. Fraction used for NMR interaction studies in green.

PolyE$_6$K$_3$Y		
Fraction/Batch	Average D (10^{-11} m^2/s)	M$_p$ (kDa) [20-50 kDa Batch A]
2A	2.83 ± 0.04	100.28
3A	4.09 ± 0.08	45.54
4A	6.32 ± 0.06	17.92
5A	10.01 ± 0.29	6.68

Tables S14a-c. Measured D, T_1 and T_2 values for the four fractions obtained by SEC for batch A of the 20-50 kDa polyK$_4$Y. All the samples were 0.6 mM polyK$_4$Y, 15 mM HEPES-d$_{18}$, 50 mM NaCl, pH=7.0.

**PolyK$_4$Y batch A**					
**Fraction**	**D $H_{ar(ortho)(Y)}$ (10^{-11} m^2/s)**	**D $H_{ar(meta)(Y)}$ (10^{-11} m^2/s)**	**D $H_{\beta(Y)+\varepsilon(K)}$ (10^{-11} m^2/s)**	**D $H_{\beta+\delta(K)}$ (10^{-11} m^2/s)**	**D $H_{\gamma(K)}$ (10^{-11} m^2/s)**
PolyK$_4$Y batch A prior to SEC	7.02 ± 3.4·10^{-3}	6.99 ± 2.9·10^{-3}	7.38 ± 8.1·10^{-4}	7.21 ± 5.2·10^{-4}	7.46 ± 1.0·10^{-3}
f1 (1A)	5.21 ± 1.7·10^{-2}	5.17 ± 1.4·10^{-2}	5.38 ± 3.7·10^{-3}	5.21 ± 3.2·10^{-3}	5.29 ± 6.2·10^{-3}
f2 (2A)	6.37 ± 1.5·10^{-2}	6.39 ± 1.3·10^{-2}	6.68 ± 4.2·10^{-3}	6.43 ± 3.3·10^{-3}	6.66 ± 6.7·10^{-3}
f3 (3A)	7.83 ± 2.0·10^{-2}	7.75 ± 1.7·10^{-2}	8.33 ± 6.2·10^{-3}	8.07 ± 4.3·10^{-3}	8.18 ± 9.1·10^{-3}
f4 (4A)	9.53 ± 2.7·10^{-2}	9.45 ± 2.3·10^{-2}	10.34 ± 9.3·10^{-3}	10.23 ± 6.5·10^{-3}	10.41 ± 1.3·10^{-2}

**PolyK$_4$Y batch A**					
**Fraction**	**T_1 $H_{ar(ortho)(Y)}$ (s)**	**T_1 $H_{ar(meta)(Y)}$ (s)**	**T_1 $H_{\beta(Y)+\varepsilon(K)}$ (s)**	**T_1 $H_{\beta+\delta(K)}$ (s)**	**T_1 $H_{\gamma(K)}$ (s)**
PolyK$_4$Y batch A prior to SEC	1.65 ± 8.7·10^{-15}	2.57 ± 5.5·10^{-3}	0.82 ± 3.6·10^{-4}	0.50 ± 1.8·10^{-4}	0.47 ± 6.8·10^{-4}
f1 (1A)	1.52 ± 1.8·10^{-14}	1.97 ± 2.2·10^{-3}	0.77 ± 3.0·10^{-4}	0.47 ± 1.4·10^{-4}	0.43 ± 2.4·10^{-4}
f2 (2A)	1.51 ± 1.8·10^{-14}	1.97 ± 1.6·10^{-3}	0.75 ± 2.2·10^{-4}	0.46 ± 1.2·10^{-4}	0.43 ± 2.2·10^{-4}
f3 (3A)	1.55 ± 1.8·10^{-14}	2.06 ± 1.9·10^{-3}	0.76 ± 2.3·10^{-4}	0.47 ± 1.3·10^{-4}	0.43 ± 2.3·10^{-4}
f4 (4A)	1.53 ± 1.8·10^{-14}	2.04 ± 2.1·10^{-3}	0.74 ± 2.8·10^{-4}	0.47 ± 1.4·10^{-4}	0.43 ± 2.6·10^{-4}

**PolyK$_4$Y batch A**					
**Fraction**	**T_2 $H_{ar(ortho)(Y)}$ (s)**	**T_2 $H_{ar(meta)(Y)}$ (s)**	**T_2 $H_{\beta(Y)+\varepsilon(K)}$ (s)**	**T_2 $H_{\beta+\delta(K)}$ (s)**	**T_2 $H_{\gamma(K)}$ (s)**
PolyK$_4$Y batch A prior to SEC	0.31 ± 2.2·10^{-4}	0.36 ± 2.2·10^{-4}	0.24 ± 4.0·10^{-5}	0.18 ± 1.8·10^{-5}	0.19 ± 3.7·10^{-5}
f1 (1A)	0.27 ± 3.1·10^{-4}	0.29 ± 2.9·10^{-4}	0.22 ± 5.7·10^{-5}	0.15 ± 2.3·10^{-5}	0.15 ± 4.4·10^{-5}
f2 (2A)	0.29 ± 2.6·10^{-4}	0.31 ± 2.3·10^{-4}	0.24 ± 5.3·10^{-5}	0.16 ± 2.0·10^{-5}	0.17 ± 4.1·10^{-5}
f3 (3A)	0.30 ± 3.4·10^{-4}	0.34 ± 2.8·10^{-4}	0.25 ± 5.8·10^{-5}	0.18 ± 2.4·10^{-5}	0.19 ± 5.5·10^{-5}
f4 (4A)	0.34 ± 3.2·10^{-4}	0.36 ± 2.5·10^{-4}	0.27 ± 6.9·10^{-5}	0.19 ± 3.2·10^{-5}	0.20 ± 7.0·10^{-5}

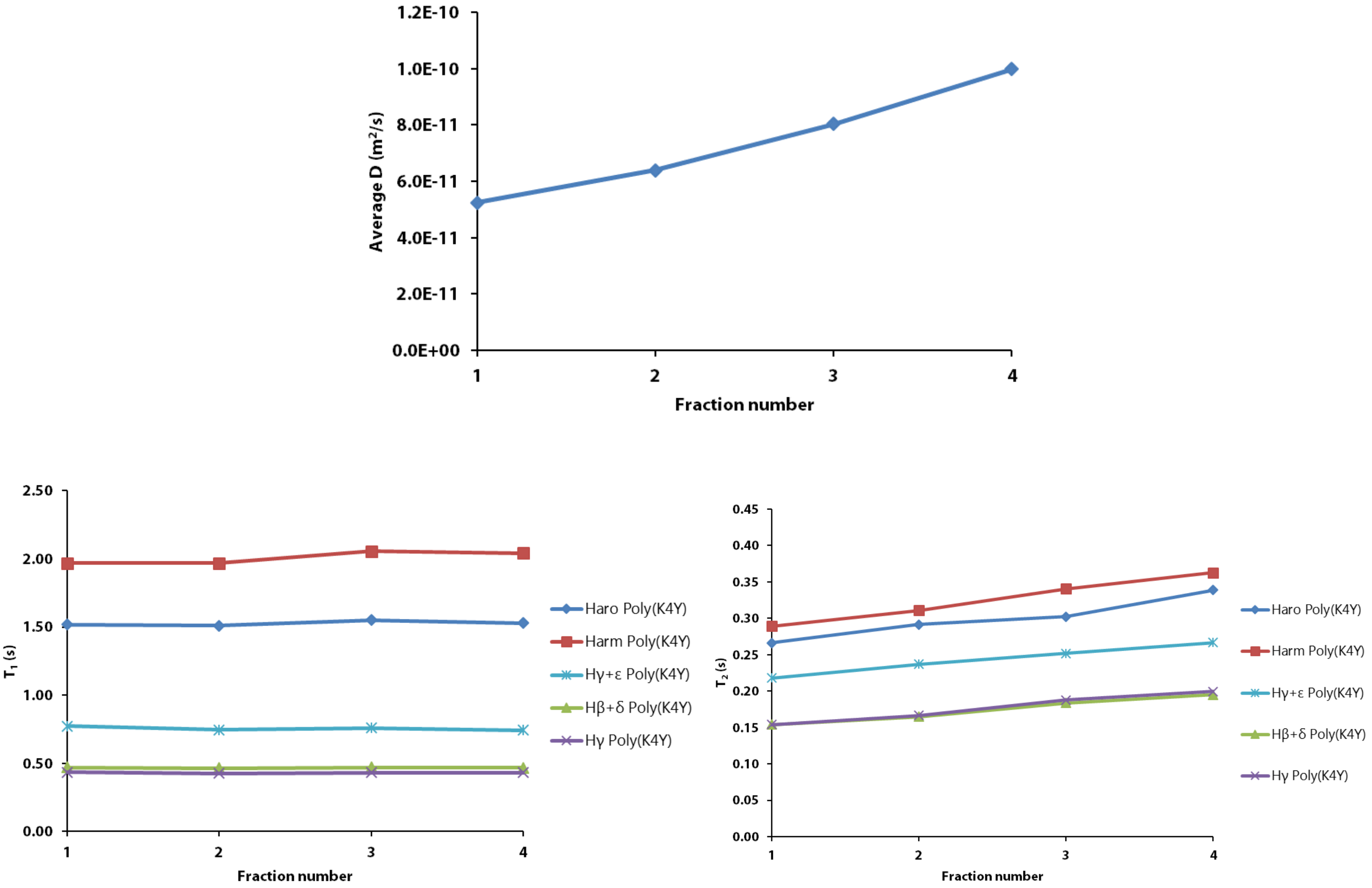

Figure S23. Representation of the D (average of the different protons), T_1 and T_2 values tabulated in tables S14a-c.

Tables S15a-c. Measured D, T_1 and T_2 values for four of the six fractions obtained by SEC for batch A of the 20-50 kDa polyE_6K_3Y. All the samples were 0.6 mM polyE_6K_3Y, 15 mM HEPES-d_{18}, 50 mM NaCl, pH=7.0.

**PolyE_6K_3Y batch A**							
**Fraction**	**D $H_{ar(ortho)(Y)}$ (10^{-11} m^2/s)**	**D $H_{ar(meta)(Y)}$ (10^{-11} m^2/s)**	**D $H_{\beta(Y)+\varepsilon(K)}$ (10^{-11} m^2/s)**	**D $H_{\gamma(E)}$ (10^{-11} m^2/s)**	**D $H_{\beta(E)}$ (10^{-11} m^2/s)**	**D $H_{\beta+\delta(K)}$ (10^{-11} m^2/s)**	**D $H_{\gamma(K)}$ (10^{-11} m^2/s)**
f2 (2A)	$2.86 \pm 7.8\cdot10^{-3}$	$2.83 \pm 6.2\cdot10^{-3}$	$2.97 \pm 3.3\cdot10^{-3}$	$2.86 \pm 2.3\cdot10^{-3}$	$2.87 \pm 1.9\cdot10^{-3}$	$2.88 \pm 2.7\cdot10^{-3}$	$2.90 \pm 4.7\cdot10^{-3}$
f3 (3A)	$4.00 \pm 1.0\cdot10^{-2}$	$3.99 \pm 8.4\cdot10^{-3}$	$4.18 \pm 3.8\cdot10^{-3}$	$4.05 \pm 2.8\cdot10^{-3}$	$4.09 \pm 2.4\cdot10^{-3}$	$4.13 \pm 3.4\cdot10^{-3}$	$4.19 \pm 5.8\cdot10^{-3}$
f4 (4A)	$6.27 \pm 1.5\cdot10^{-2}$	$6.25 \pm 1.2\cdot10^{-2}$	$6.45 \pm 5.2\cdot10^{-2}$	$6.33 \pm 4.2\cdot10^{-3}$	$6.33 \pm 3.2\cdot10^{-3}$	$6.29 \pm 4.2\cdot10^{-3}$	$6.32 \pm 6.7\cdot10^{-3}$
f5 (5A)	$10.32 \pm 4.5\cdot10^{-2}$	$10.34 \pm 4.1\cdot10^{-2}$	$10.22 \pm 1.6\cdot10^{-2}$	$9.98 \pm 1.3\cdot10^{-2}$	$9.92 \pm 1.0\cdot10^{-2}$	$9.69 \pm 1.2\cdot10^{-2}$	$9.61 \pm 2.0\cdot10^{-2}$

**PolyE_6K_3Y batch A**							
**Fraction**	**T_1 $H_{ar(ortho)(Y)}$ (s)**	**T_1 $H_{ar(meta)(Y)}$ (s)**	**T_1 $H_{\beta(Y)+\varepsilon(K)}$ (s)**	**T_1 $H_{\gamma(E)}$ (s)**	**T_1 $H_{\beta(E)}$ (s)**	**T_1 $H_{\beta+\delta(K)}$ (s)**	**T_1 $H_{\gamma(K)}$ (s)**
f2 (2A)	$1.37 \pm 1.6\cdot10^{-3}$	$1.76 \pm 1.8\cdot10^{-14}$	$0.70 \pm 2.9\cdot10^{-4}$	$0.42 \pm 1.2\cdot10^{-4}$	$0.44 \pm 1.2\cdot10^{-4}$	$0.48 \pm 1.9\cdot10^{-4}$	$0.43 \pm 2.7\cdot10^{-4}$
f3 (3A)	$1.45 \pm 6.3\cdot10^{-5}$	$1.86 \pm 1.8\cdot10^{-14}$	$0.71 \pm 2.9\cdot10^{-4}$	$0.43 \pm 1.3\cdot10^{-4}$	$0.44 \pm 1.1\cdot10^{-4}$	$0.47 \pm 1.8\cdot10^{-4}$	$0.43 \pm 2.6\cdot10^{-4}$
f4 (4A)	$1.35 \pm 1.8\cdot10^{-3}$	$1.75 \pm 1.8\cdot10^{-14}$	$0.69 \pm 2.4\cdot10^{-4}$	$0.38 \pm 1.1\cdot10^{-4}$	$0.42 \pm 9.4\cdot10^{-5}$	$0.48 \pm 1.6\cdot10^{-4}$	$0.44 \pm 2.3\cdot10^{-4}$
f5 (5A)	$1.34 \pm 3.2\cdot10^{-3}$	$1.79 \pm 1.8\cdot10^{-14}$	$0.76 \pm 4.6\cdot10^{-4}$	$0.36 \pm 1.9\cdot10^{-4}$	$0.40 \pm 1.7\cdot10^{-4}$	$0.47 \pm 2.8\cdot10^{-4}$	$0.42 \pm 4.1\cdot10^{-4}$

**PolyE_6K_3Y batch A**							
**Fraction**	**T_2 $H_{ar(ortho)(Y)}$ (s)**	**T_2 $H_{ar(meta)(Y)}$ (s)**	**T_2 $H_{\beta(Y)+\varepsilon(K)}$ (s)**	**T_2 $H_{\gamma(E)}$ (s)**	**T_2 $H_{\beta(E)}$ (s)**	**T_2 $H_{\beta+\delta(K)}$ (s)**	**T_2 $H_{\gamma(K)}$ (s)**
f2 (2A	$0.25 \pm 2.5\cdot10^{-4}$	$0.26 \pm 2.0\cdot10^{-4}$	$0.17 \pm 4.0\cdot10^{-5}$	$0.08 \pm 1.4\cdot10^{-5}$	$0.08 \pm 1.2\cdot10^{-5}$	$0.13 \pm 2.6\cdot10^{-5}$	$0.12 \pm 3.8\cdot10^{-5}$
f3 (3A	$0.25 \pm 2.0\cdot10^{-4}$	$0.26 \pm 1.8\cdot10^{-4}$	$0.18 \pm 4.3\cdot10^{-5}$	$0.09 \pm 1.5\cdot10^{-5}$	$0.09 \pm 1.3\cdot10^{-5}$	$0.14 \pm 2.9\cdot10^{-5}$	$0.13 \pm 5.5\cdot10^{-5}$
f4 (4A	$0.28 \pm 3.2\cdot10^{-4}$	$0.29 \pm 2.5\cdot10^{-4}$	$0.18 \pm 3.7\cdot10^{-5}$	$0.08 \pm 1.3\cdot10^{-5}$	$0.09 \pm 1.1\cdot10^{-5}$	$0.15 \pm 2.5\cdot10^{-5}$	$0.13 \pm 3.5\cdot10^{-5}$
f5 (5A	$0.31 \pm 4.6\cdot10^{-4}$	$0.33 \pm 4.4\cdot10^{-4}$	$0.20 \pm 7.2\cdot10^{-5}$	$0.09 \pm 1.5\cdot10^{-5}$	$0.10 \pm 2.4\cdot10^{-5}$	$0.17 \pm 5.0\cdot10^{-5}$	$0.15 \pm 8.2\cdot10^{-5}$

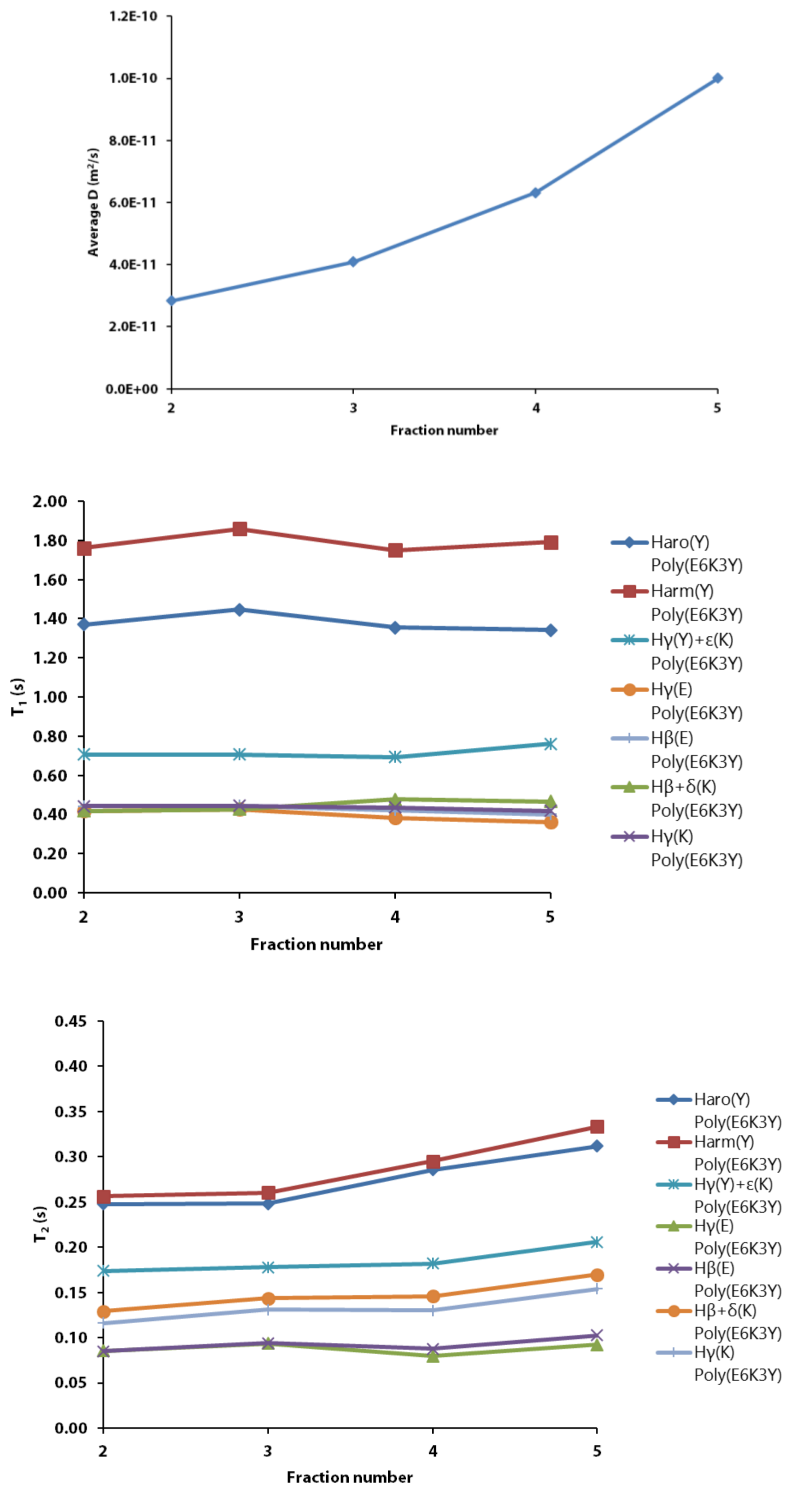

Figure S24. Representation of the D, T_1 and T_2 values tabulated in tables S15a-c.

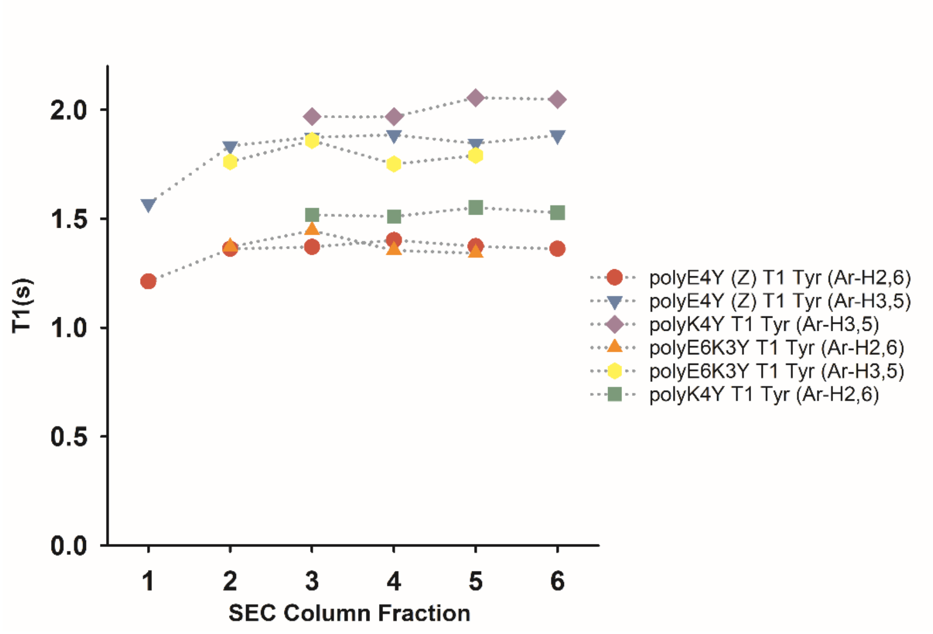

Figure S25. Representation of the variation of T_1 relaxation time in collected fractions. To account for the differences in molecular weight, greater for polyE$_4$Y and polyE$_6$K$_3$Y than for polyK$_4$Y, fractions (3-6) of polyK$_4$Y correspond to collected fractions 1-4.

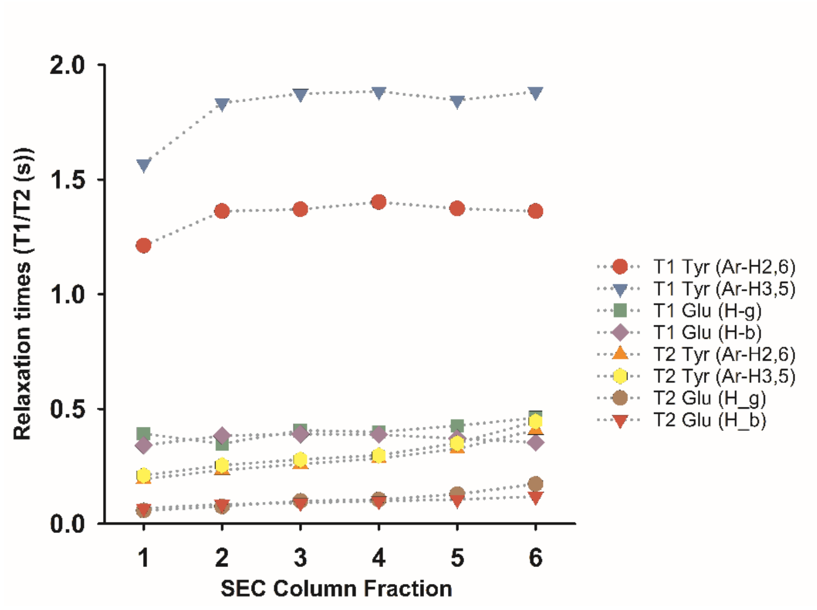

Figure S26. Comparison of T_1 and T_2 relaxation times of Tyr and Glu resonances in polyE$_4$Y.

Studies of cages binding to polypeptides by NMR

Table S16. Solubility tests in the media used for NMR screening for mixtures with different compositions of the CyLys cage and polyE$_4$Y. Samples prepared in 15 mM HEPES-d18, D_2O, pH=7.0 buffer.

Entry	CyLys conc. (mM])	Poly(E4Y) conc. (mM)	Cage:PP	NaCl conc. (mM)	pH before PP addition	Result
1	1.48	0.70	2:1	-	7.0	Precipitated upon addition
2	1.48	0.14	10:1	-	7.0	Precipitated upon addition
3	1.77	0.036	50:1	-	7.0	Precipitated after 1d
4	0.70	0.35	2:1	-	7.0	Turbid, eventually dissolved
5	0.40	0.20	2:1	-	7.0	Dissolved completely
6	0.42	3.00	1:7	50	7.35	Dissolved completely
7	0.75	3.00	1:4	50	7.35	Dissolved completely
8	1.07	3.00	1:2.8	50	7.35	Dissolved completely
9	1.30	0.65	2:1	50	7.35	Dissolved completely
10	1.30	0.65	2:1	-	7.35	Dissolved completely
11	1.48	0.72	2:1	-	7.35	Precipitate (small quantity)
12	1.48	0.33	4.5:1	-	7.35	Precipitate (small quantity)
13	1.61	0.72	2.2:1	-	4.80	Precipitated upon addition

Table S17. Solubility tests in the media used for NMR screening for mixtures with the same composition of the CyLys cage and polyE$_4$Y (1.43 mM CyLys, 0.73 mM polyE$_4$Y, 15 mM HEPES-d$_{18}$) for different pH values before polypeptide addition.

Entry	pH before polypeptide addition	Result
1	4.96	Precipitated upon addition
2	5.48	Dissolved completely
3	6.06	Dissolved completely
4	6.52	Dissolved completely
5	6.93	Precipitate (small quantity)
6	7.50	Precipitate (small quantity)
7	7.96	Precipitated upon addition
8	8.44	Precipitated upon addition
9	8.93	Precipitate (small quantity)

Table S18. Translational diffusion coefficients measured by NMR for the combination of the three cages (CyHis, CyLys and CyAsp) with some of the three types of polypeptides (polyE$_4$Y fc3, polyK$_4$Y fc1 and polyE$_6$K$_3$Y fc3), in D_2O, 15 mM HEPES-d$_{18}$, 50 mM NaCl, pH=7 or 7.5

entry	cage	polypeptide	Sample composition (mM)	D_{cage}[1] (10^{-10} m^2/s)	$D_{polypeptide}$
1	**CyAsp**	--	0.4	2.24-2.31	--
2	**CyAsp**	polyE$_4$Y	0.4:2.0	2.06-2.22	0.43 (Tyr)[2], 0.33 (Asp)[5]
3	**CyAsp**	polyK$_4$Y	0.4:0.5	2.02-1.93	1.01 (Tyr)[2], 0.64 (Lys+Tyr)[4]
	**CyAsp**	polyK$_4$Y	0.4:2.0	**1.41-1.35**	0.60 (Tyr)[2], 0.58 (Lys+Tyr)[4]
4	**CyAsp**	polyE$_6$K$_3$Y	0.4:0.5	2.14-2.26	0.42 (Lys+Tyr)[4]
5	**CyHis**	--	0.4	2.24-2.31	--
6	**CyHis**	polyE$_4$Y	0.4:0.8	2.05-2.12	0.38 (Asp)[5]
7	**CyHis**	polyK$_4$Y	0.4:0.8	2.06-2.21	n.a. (overlapping)
8	**CyHis**	polyE$_6$K$_3$Y	0.4:0.8	**1.69-2.01**	0.47 (Lys+Tyr)[4]
9	**CyLys**	--	0.4	2.04-2.25	
10	**CyLys**	polyE$_4$Y	0.4:2.0	**0.50-0.63**	0.51 (Tyr)[2], 0.50 (Asp)[5]
11	**CyLys**	polyE$_6$K$_3$Y	0.4:0.8	**1.63-1.76**	0.72 (Tyr)[2], 0.60 (Asp)[5]
12	**--**	polyE$_4$Y (fc3)	0.6	--	0.40 (Tyr)[2], 0.40 (Asp)[3]
13	**--**	polyK$_4$Y (fc1)	0.6	--	0.52 (Tyr)[2], 0.54 (Lys+Tyr)[4]
14	**--**	polyE$_6$K$_3$Y (fc3)	0.6	--	0.40 (Tyr)[2], 0.42 (Lys+Tyr)[4]

[1] Minimum and maximum D value for different cage resonances [2] Tyr aromatic resonances [3] β/γCH Asp resonances [4] Tyr βCH and Lys εCH resonances [5] γCH Asp resonances

Table S19. T_2 relaxation times measured by NMR for the combination of the three cages (CyHis, CyLys and CyAsp) with some of the three types of polypeptides (polyE$_4$Y fc3, polyK$_4$Y fc1 and polyE$_6$K$_3$Y fc3), in D_2O, 15 mM HEPES-d$_{18}$, 50 mM NaCl, pH=7 or 7.5

entry	cage	polypeptide	Sample composition (mM)	T_2 H_{ar}	T_2 H_{bz}	T_2 H_α	T_2 H_β
1	**CyAsp**	--	0.4	231.1	99.0	205.0	183.7
2	**CyAsp**	polyE$_4$Y	0.4:2.0	252.1	104.1	226.1[1]	192.1
3	**CyAsp**	polyK$_4$Y	0.4:0.5	210.7	83.02	194.0[1]	155.0
	**CyAsp**	polyK$_4$Y	0.4:2.0	**176.5**	**59.6**	**173.0[1]**	**123.0**
4	**CyAsp**	polyE$_6$K$_3$Y	0.4:0.5	218.8	95.3	197.6	174.5
5	**CyHis**	--	0.4	412.0	125.2	308.1	176.6
		--	0.4 (pH 7.5)	321.3	89.1	200.9	117.2
6	**CyHis**	polyE$_4$Y	0.4:0.8 (pH 7.5)	312.5	96.4	235.6	119.4
7	**CyHis**	polyK$_4$Y	0.4:0.8	396.7	115.3	244.9	170.7[1]
8	**CyHis**	polyE$_6$K$_3$Y	0.4:0.8	**244.4**	**64.9**	**243.2**	**169.0**
9	**CyLys**	--	0.4	292.8	102.9	406.8[2]	--[1]
10	**CyLys**	polyE$_4$Y	0.4:2.0	**42.4**	**12.4**	**70.6[2]**	--[1]
11	**CyLys**	polyE$_6$K$_3$Y	0.4:0.8	**204.5**	**66.7**	--[1]	--[1]
				**H_{ar}**	**H_{ar}**	**H_β Asp**	**H_γ Asp**
12	**--**	polyE$_4$Y (fc3)	0.6	259.0	274.0	90.6	98.5
13	**--**	polyK$_4$Y (fc1)	0.6	266.3	289.0		
14	**--**	polyE$_6$K$_3$Y (fc3)	0.6	248.2	260.3	94.2	93.8

[1] Overlapped resonances [2] Hε Lys

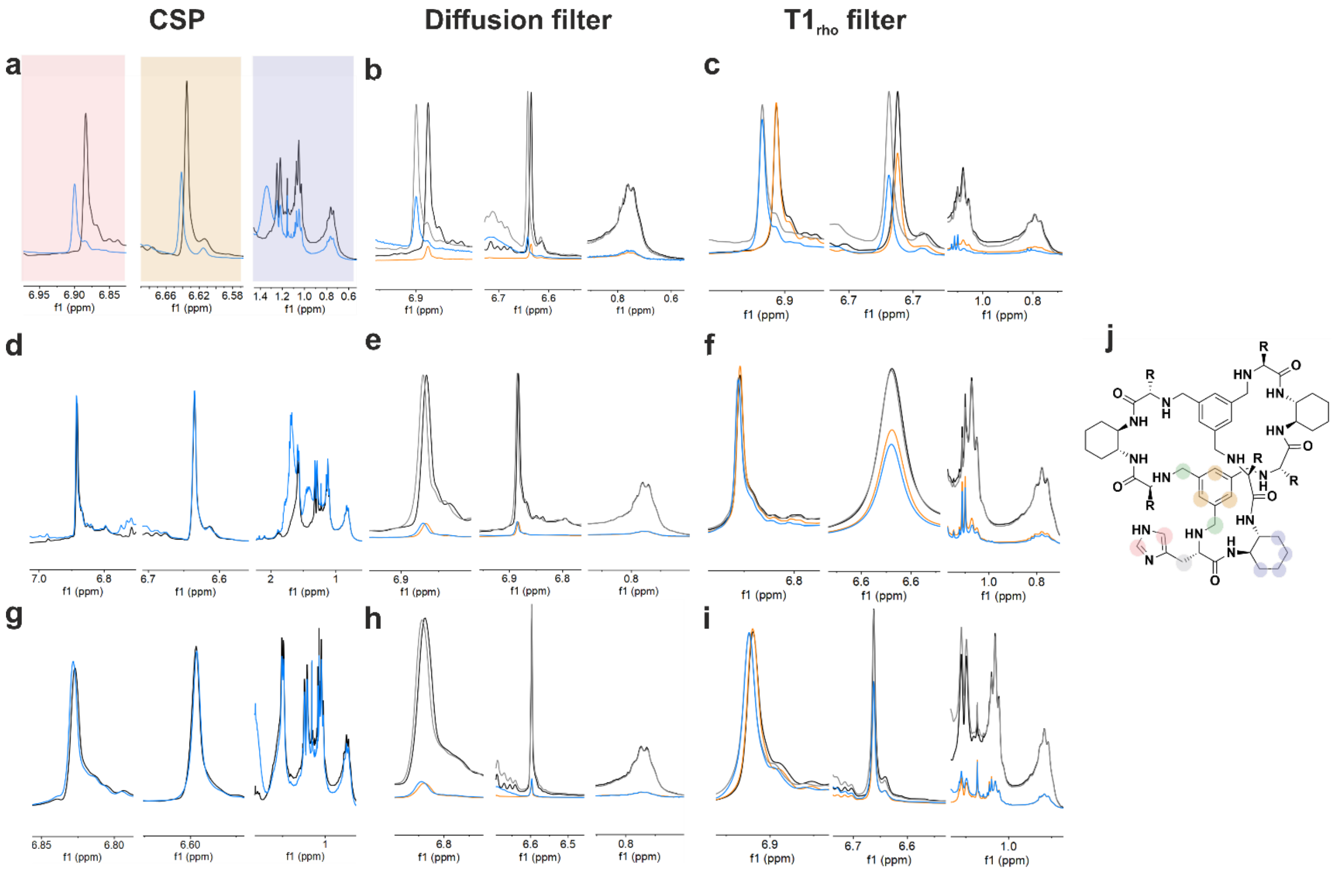

Figure S27. NMR studies of CyHis binding to polypeptides using chemical shift changes and relaxation/diffusion-edited approaches. (a, d, g) Black (without PP)/Blue (with PP) chemical shift changes.(b-c, e-f, h-i) Black /Orange, cage without polypeptide spectra, low field gradient (5%) or short T1ρ filter (10 ms)/ high field gradient (95%) or long T1ρ filter (200 ms). Grey/Blue, cage with polypeptide spectra, low field gradient (5%) or short T1ρ filter (10 ms)/ high field gradient (95%) or long T1ρ filter (200 ms). (a, b, c) polyE_6K_3Y; (d, e, f) polyK_4Y; (g, h, i) polyE_4Y.(j) The shifted protons are mapped out on the cage structure.

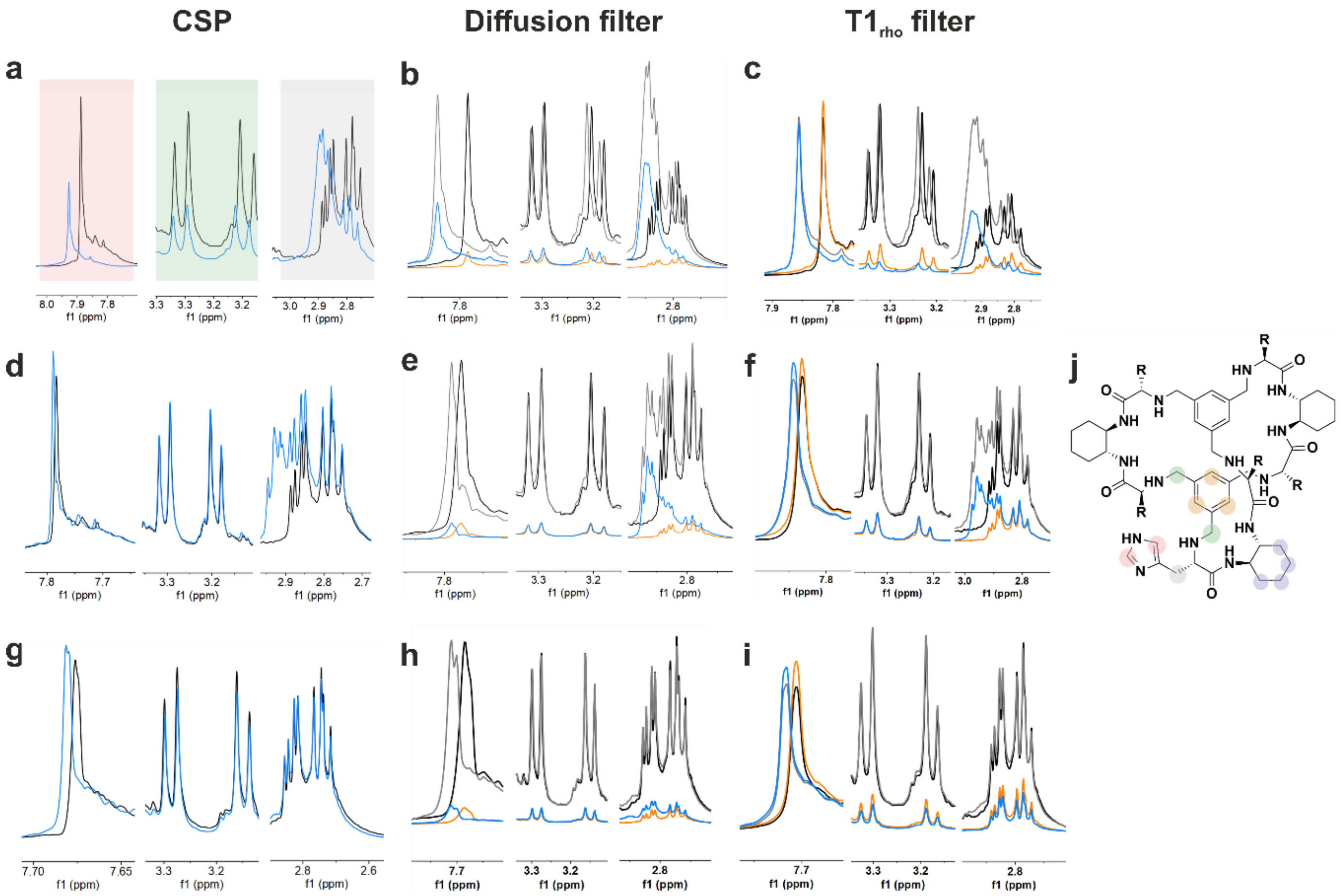

Figure S28 NMR studies of CyHis binding to polypeptides using chemical shift changes and relaxation/diffusion-edited approaches. (a, d, g) Black (without PP)/Blue (with PP) chemical shift changes.(b-c, e-f, h-i) Black /Orange, cage without polypeptide spectra, low field gradient (5%) or short T1ρ filter (10 ms)/ high field gradient (95%) or long T1ρ filter (200 ms). Grey/Blue, cage with polypeptide spectra, low field gradient (5%) or short T1ρ filter (10 ms)/ high field gradient (95%) or long T1ρ filter (200 ms). (a, b, c) polyE$_6$K$_3$Y; (d, e, f) polyK$_4$Y; (g, h, i) polyE$_4$Y.(j) The shifted protons are mapped out on the cage structure.

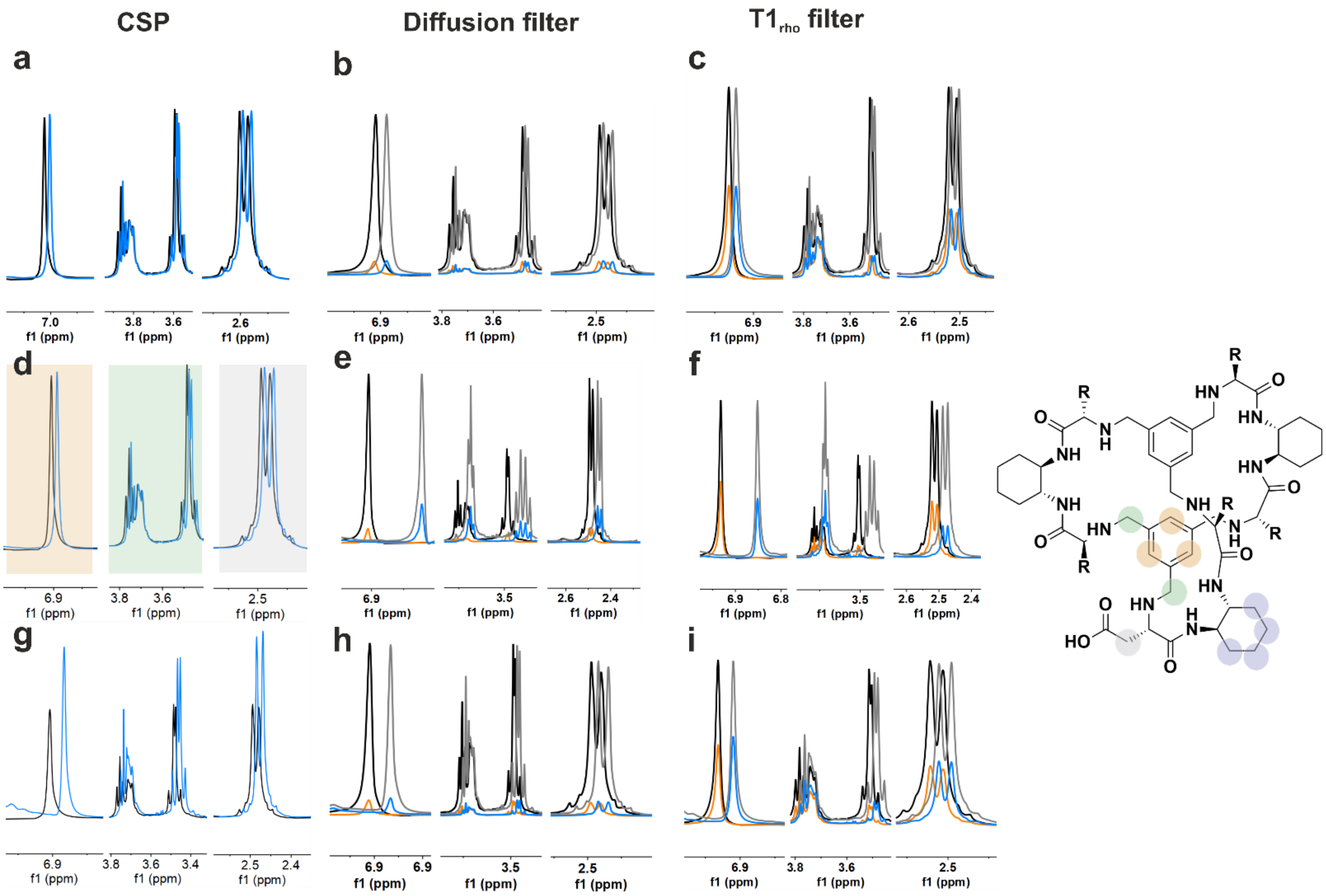

Figure S29. NMR studies of CyAsp binding to polypeptides using chemical shift changes and relaxation/diffusion-edited approaches. Black (without PP)/Blue (with PP) chemical shift changes. Black/Orange, cage without polypeptide spectra, low field gradient (5%) or short $T_{1\rho}$ filter (10 ms)/ high field gradient (95%) or long $T_{1\rho}$ filter (200 ms). Grey/Blue, cage with polypeptide spectra, low field gradient (5%) or short $T_{1\rho}$ filter (10 ms)/ high field gradient (95%) or long $T_{1\rho}$ filter (200 ms). (a, b, c) polyE$_6$K$_3$Y; (d, e, f) polyK$_4$Y; (g, h, i) polyE$_4$Y.

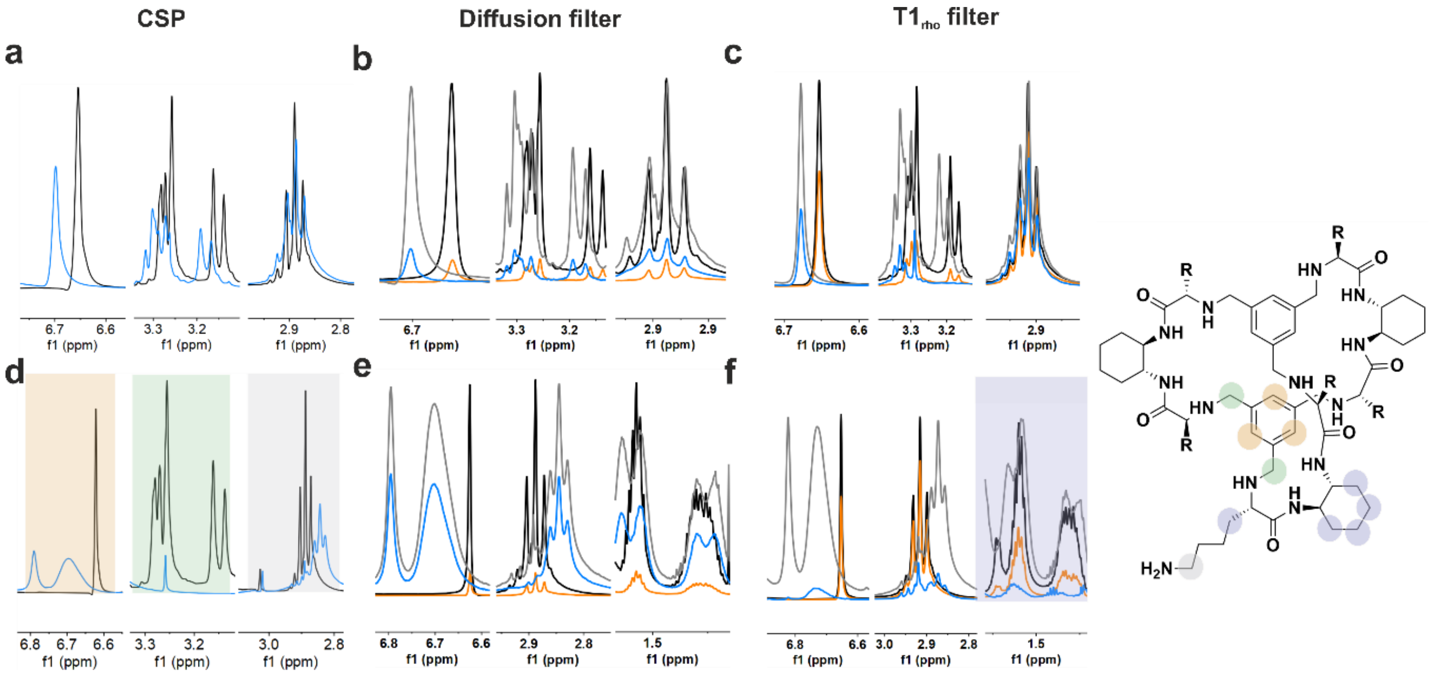

Figure S30. NMR studies of CyLys binding to polypeptides using chemical shift changes and relaxation/diffusion-edited approaches. Black (without PP)/Blue (with PP) chemical shift changes. Black/Orange, cage without polypeptide spectra, low field gradient (5%) or short $T_{1\rho}$ filter (10 ms)/ high field gradient (95%) or long $T_{1\rho}$ filter (200 ms). Grey/Blue, cage with polypeptide spectra, low field gradient (5%) or short $T_{1\rho}$ filter (10 ms)/ high field gradient (95%) or long $T_{1\rho}$ filter (200 ms). (a, b, c) polyE$_6$K$_3$Y; (d, e, f) polyE$_4$Y.

Table S20. Quantitative analysis of the relaxation/diffusion-edited NMR experiments for the combination of the three cages (CyHis, CyLys and CyAsp) with some of the three types of polypeptides (polyE_4Y fc3, polyK_4Y fc1 and polyE_6K_3Y fc3). The values are calculated for the cages tyrosines resonances. For CysAsp cage, the negative values indicate reverse behavior, with intensity increase/decrease after addition of polypeptide for relaxation and diffusion filtered experiments, respectively. Diffusion, no binding < 25%, weak < 50%, medium < 80 % and strong > 80%. relaxation, no binding < 15%, weak < 40%, medium < 60% and strong > 60%.

**entry**	**cage**	**polypeptide**	**Sample composition (mM)**	**$T_{1\rho}$ reduction (%)**	**T_2 reduction (%)**	**D change (%)**
1	**CyAsp**	polyE_4Y	0.4:2.0	-10.6	-18.9	17.3
3	**CyAsp**	polyK_4Y	0.4:2.0	23.6	32.0	62.4
4	**CyAsp**	polyE_6K_3Y	0.4:0.5	0.3	5.3	8.2
5	**CyHis**	polyE_4Y	0.4:0.8 (pH 7.5)	8.6	17.6	22.3
6	**CyHis**	polyK_4Y	0.4:0.8	-8.3	-13.8	11.6
7	**CyHis**	polyE_6K_3Y	0.4:0.8	15.0	15.4	51.2
8	**CyLys**	polyE_4Y	0.4:2.0	98.1	100	80.3
9	**CyLys**	polyE_6K_3Y	0.4:0.8	31.9	41.1	34.6

Molecular modeling of supramolecular complexes

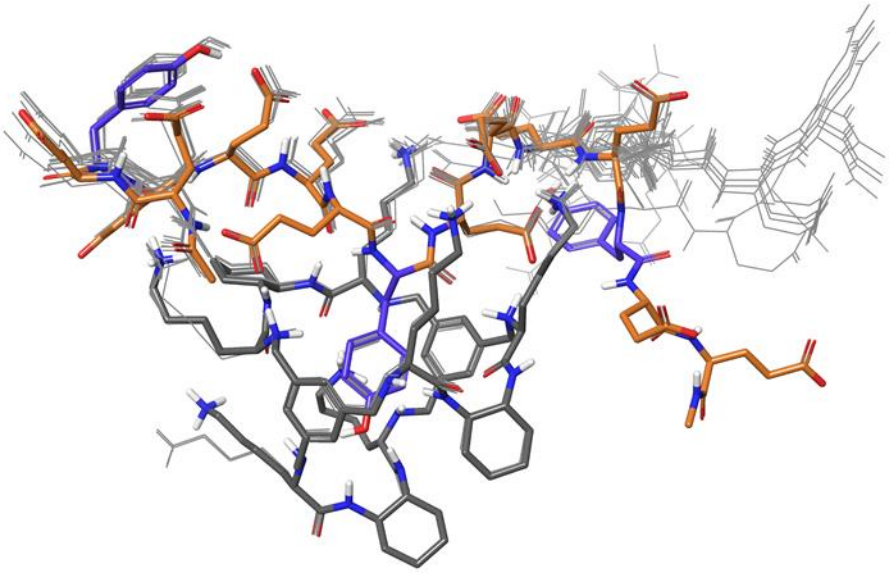

Figure S31. Superposition of the local minima within 2.5 kcal/mol for the [CyLys-polyE$_4$Y] complex. The lowest-energy minimum is shown with colored sticks while the rest are shown as grey wires.

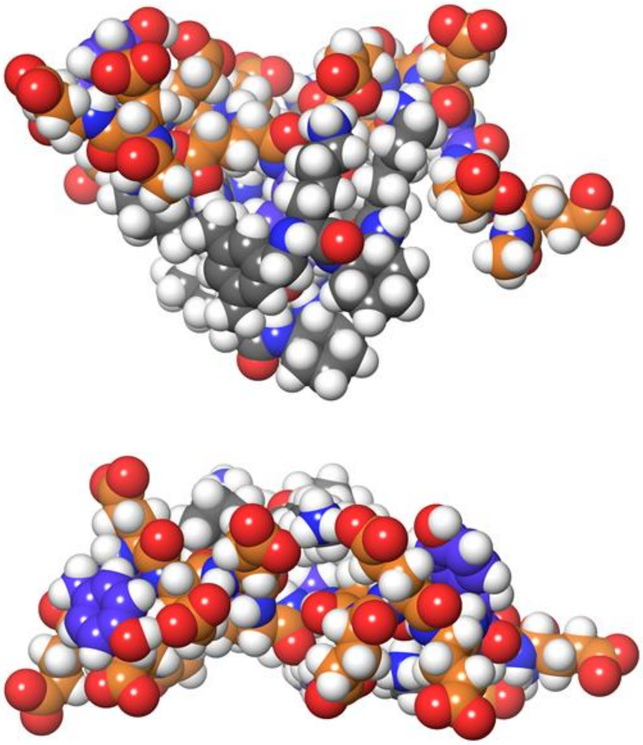

Figure S32. Two selected views of the [CyLys-polyE$_4$Y] complex model (space-filling, C-atoms: cage in grey, polyE$_4$Y in orange with **Tyr in purple**) showing the efficient inclusion of the central Tyr within the cage cavity (up) and the free exposure of the other two Tyr residues (down).

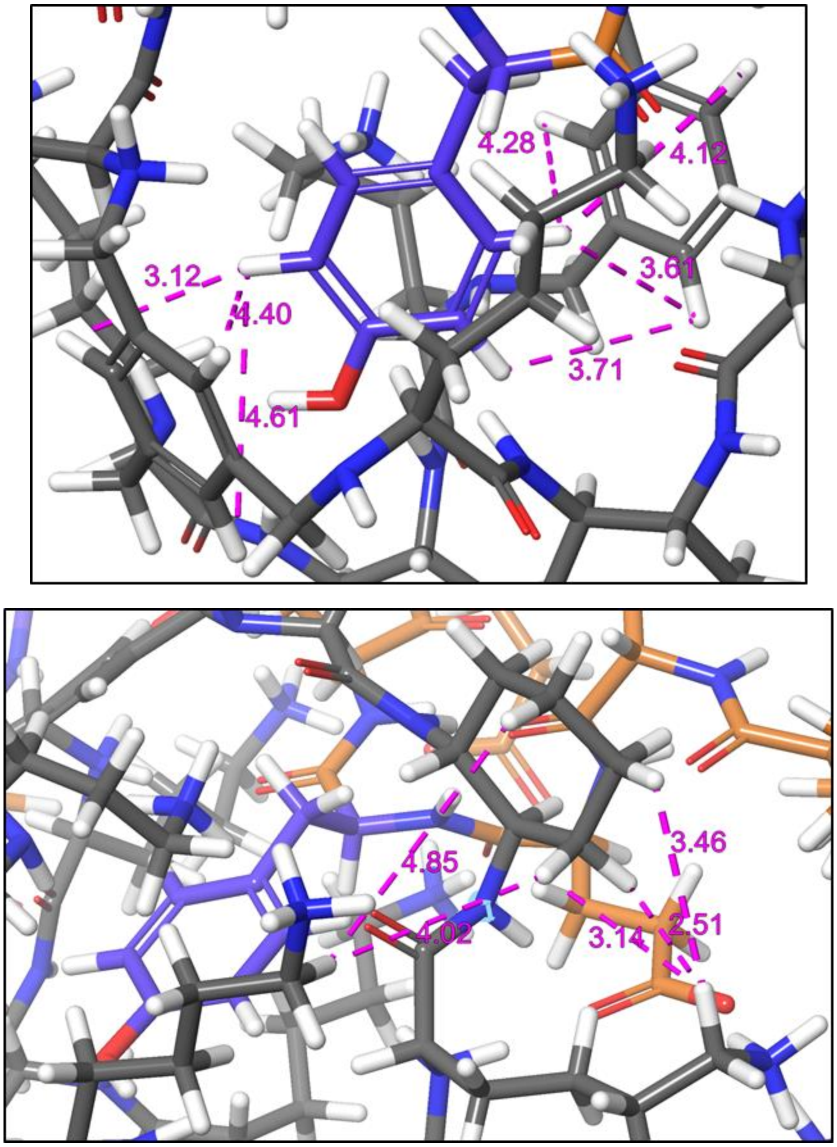

Figure S33. Distances between H atoms (Å, pink dashed lines) in the optimized minimum for the [CyLys-polyE$_4$Y] complex compatible with the observed NOEs. Up: distances for possible intermolecular NOE cross peaks between aromatic Tyr protons and cage Ar-H. Down: distances for potential intramolecular NOEs between cage cyclohexane methylenes and Lys ε-protons. Only distances <5 Å are highlighted.

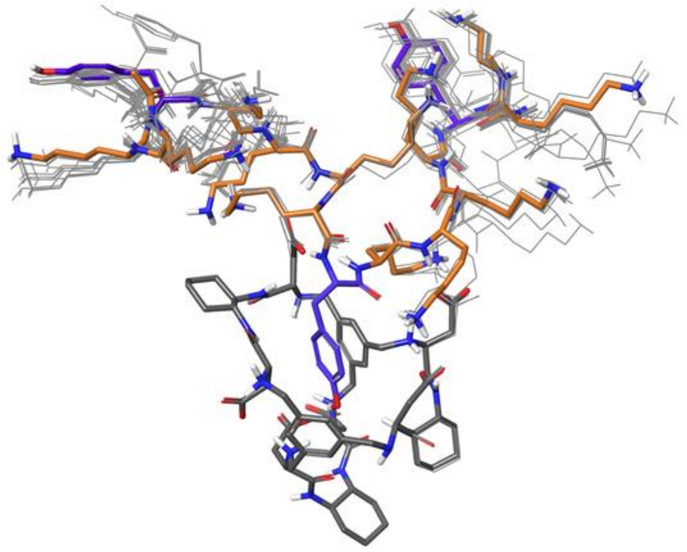

Figure S34. Superposition of the local minima within 2.5 kcal/mol for the [CyAsp-polyK_4Y] complex. The lowest-energy minimum is shown with colored sticks while the rest are shown as grey wires.

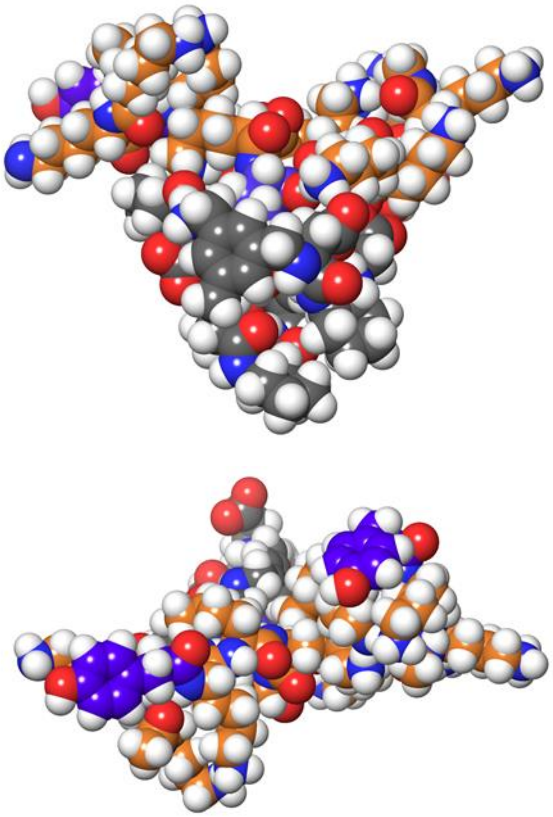

Figure S35. Two views of the [CyAsp-polyK_4Y] complex model (space filling, C-atoms: cage in grey, polyK_4Y in orange with **Tyr in purple**) displaying the inclusion of the central Tyr within the cage cavity (up) and the free exposure of the other two Tyr residues (down).

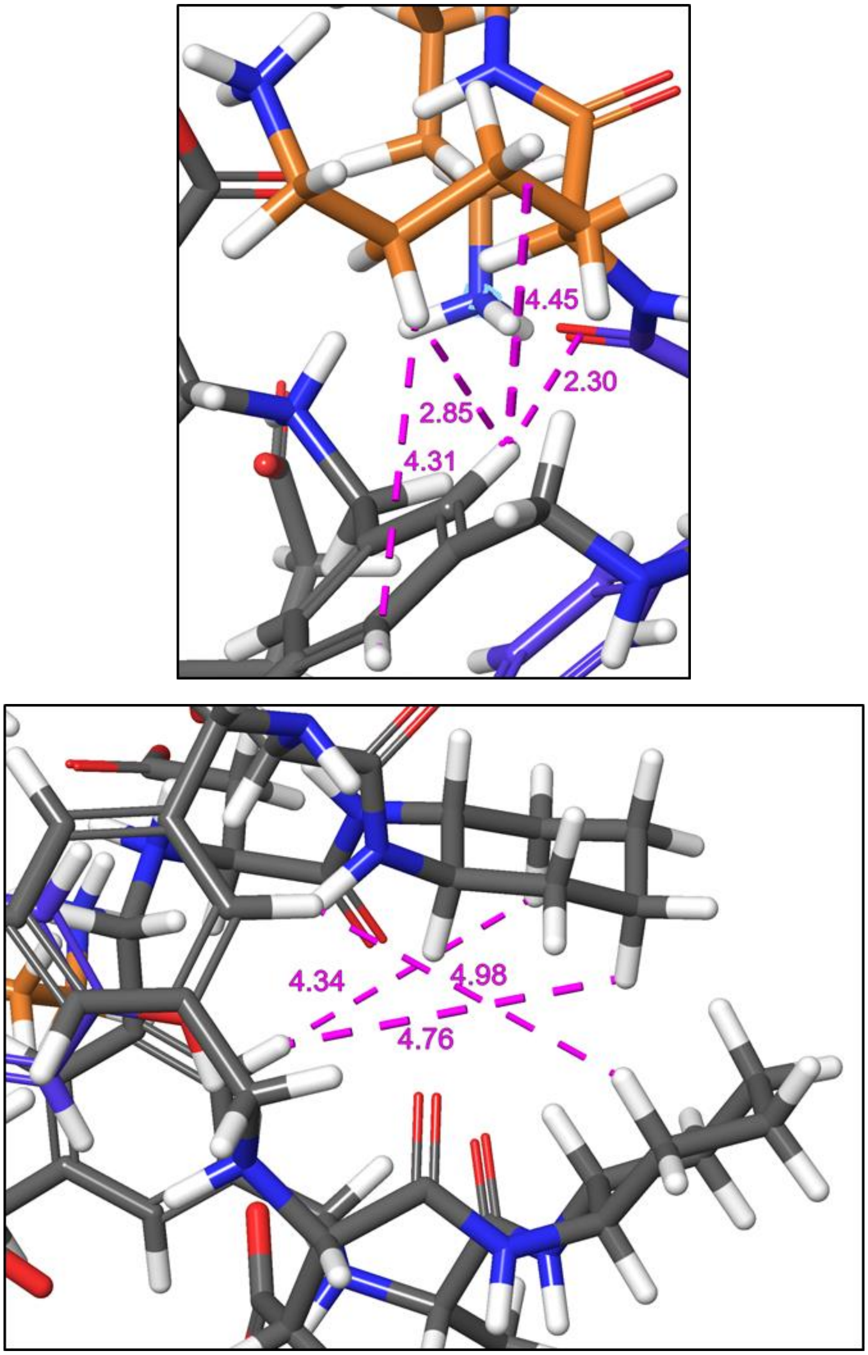

Figure S36. Distances between H atoms (Å, pink dashed lines) in the optimized minimum for the [CyAsp-polyK$_4$Y] complex compatible with the observed NOEs. Up: distances for possible intermolecular NOE cross peaks between cage Ar-H and polypeptide Lys side chain. Down: distances for potential intramolecular NOEs between cage Ar-H and cyclohexane methylenes. Only distances <5 Å are highlighted.

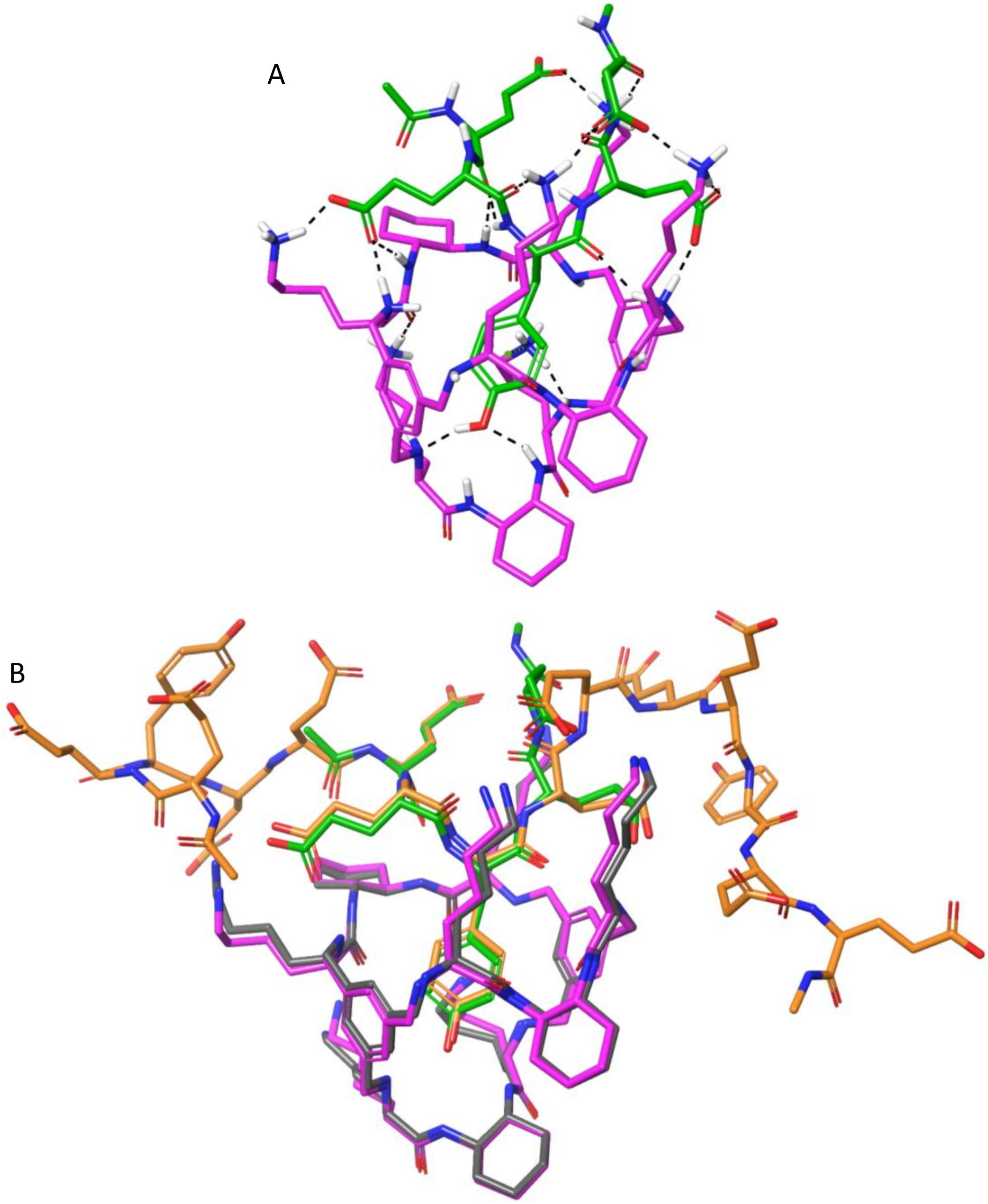

Figure S37. (A) Minimized structure for the [CyLys·Ac-EEYEE-NHMe] supramolecular complex, non-polar H-atoms are omitted for clarity and non-covalent interactions shown as dashed lines (C-atoms color code: CyLys in magenta, peptide in green). (B) Overlapping structures for the minima corresponding to [CyLys·Ac-EEYEE-NHMe] and [CyLys·Ac-EEYEEEEYEEEEYEE-NHMe] supramolecular complexes (C-atoms color code: for [CyLys·Ac-EEYEE-NHMe], CyLys in magenta, peptide in green; for [CyLys·Ac-EEYEEEEYEEEEYEE-NHMe], CyLys in grey, peptide in orange)

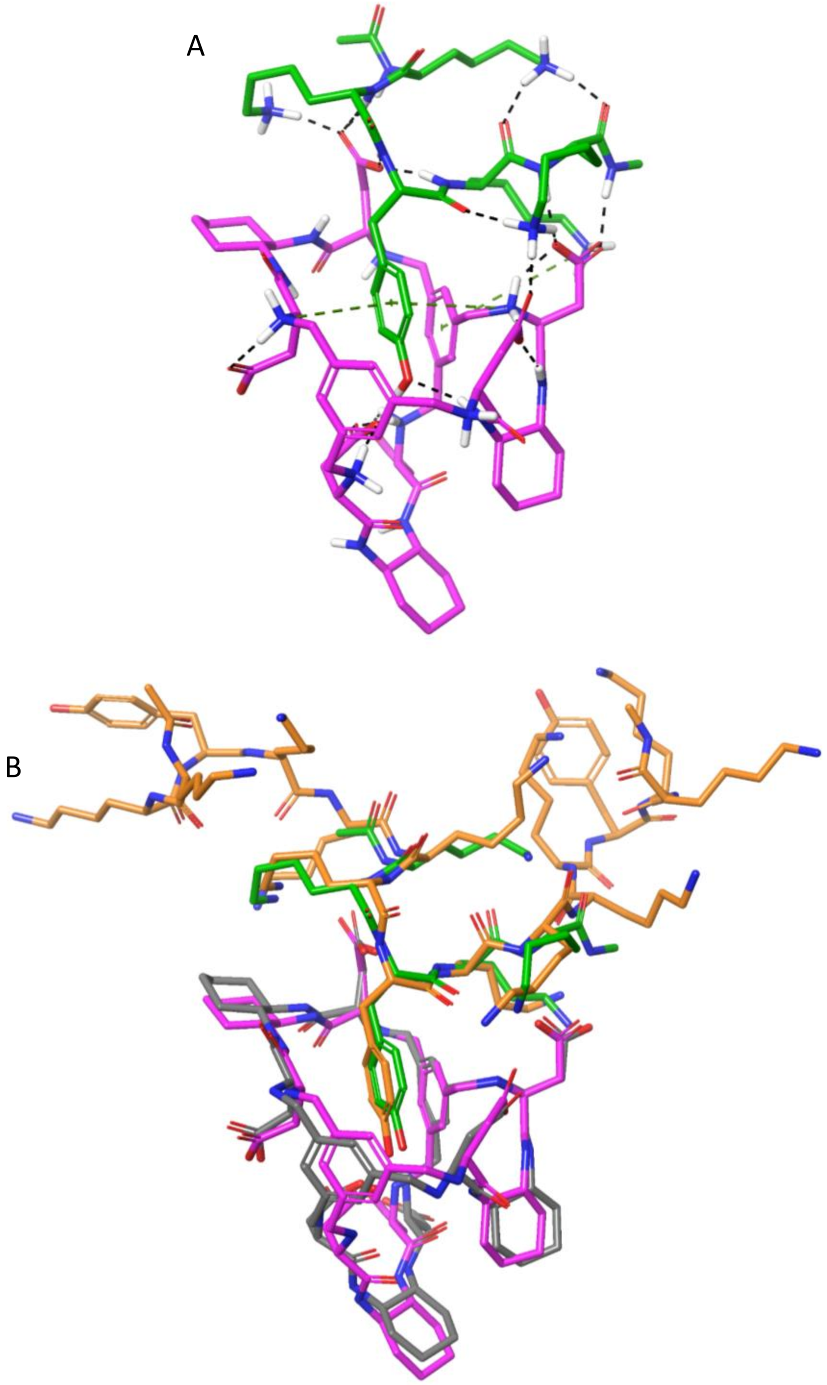

Figure S38. (A) Minimized structure for the [CyAsp·Ac-KKYKK-NHMe] supramolecular complex, non-polar H-atoms are omitted for clarity and non-covalent interactions shown as dashed lines (C-atoms color code: CyAsp in magenta, peptide in green). (B) Overlapping structures for the minima corresponding to [CyAsp·Ac-KKYKK-NHMe] and [CyAsp·Ac-KKYKKKKYKKKKYKK-NHMe] supramolecular complexes (C-atoms color code: for [CyAsp·Ac-KKYKK-NHMe], CyAsp in magenta, peptide in green; for [CyAsp·Ac-KKYKKKKYKKKKYKK-NHMe], CyAsp in grey, peptide in orange)

References

(1) Faggi, E.; Perez, Y.; Luis, S. V; Alfonso, I. Supramolecular Protection from the Enzymatic Tyrosine Phosphorylation in a Polypeptide. *Chem. Commun.* **2016,** *52 (52)*, 8142–8145. https://doi.org/10.1039/c6cc03875a.

(2) Tapia, L.; Solozabal, N.; Solà, J.; Pérez, Y.; Miller, W. T.; Alfonso, I. Modulation of Src Kinase Activity by Selective Substrate Recognition with Pseudopeptidic Cages. *Chem. - A Eur. J.* **2021**, *27 (37)*, 9542–9549. https://doi.org/10.1002/chem.202100990

(3) Kolossváry, I.; Guida, W. C. Low-Mode Conformational Search Elucidated: Application to C39H80 and Flexible Docking of 9-Deazaguanine Inhibitors into PNP. *J. Comput. Chem.* **1999**, *20* (15), 1671–1684. https://doi.org/https://doi.org/10.1002/(SICI)1096-987X(19991130)20:15<1671::AID-JCC7>3.0.CO;2-Y.

(4) Kolossváry, I.; Guida, W. C. Low Mode Search. An Efficient, Automated Computational Method for Conformational Analysis: Application to Cyclic and Acyclic Alkanes and Cyclic Peptides. *J. Am. Chem. Soc.* **1996**, *118* (21), 5011–5019. https://doi.org/10.1021/ja952478m.

(5) Goodman, J. M.; Still, W. C. An Unbounded Systematic Search of Conformational Space. *J. Comput. Chem.* **1991**, *12* (9), 1110–1117. https://doi.org/https://doi.org/10.1002/jcc.540120908.

(6) Lu, C.; Wu, C.; Ghoreishi, D.; Chen, W.; Wang, L.; Damm, W.; Ross, G. A.; Dahlgren, M. K.; Russell, E.; Von Bargen, C. D.; Abel, R.; Friesner, R. A.; Harder, E. D. OPLS4: Improving Force Field Accuracy on Challenging Regimes of Chemical Space. *J Chem Theory Comput* **2021**. https://doi.org/10.1021/acs.jctc.1c00302.

(7) Still, W. C.; Tempczyk, A.; Hawley, R. C.; Hendrickson, T. Semianalytical Treatment of Solvation for Molecular Mechanics and Dynamics. *J. Am. Chem. Soc.* **1990**, *112*, 6127–6129.

(8) Faggi, E.; Moure, A.; Bolte, M.; Vicent, C.; Luis, S. V; Alfonso, I. Pseudopeptidic Cages as Receptors for N-Protected Dipeptides. *J. Org. Chem.* **2014**, *79* (10), 4590–4601. https://doi.org/10.1021/jo500629d.

(9) Evans, R.; Deng, Z.; Rogerson, A. K.; McLachlan, A. S.; Richards, J. J.; Nilsson, M.; Morris, G. A. Quantitative Interpretation of Diffusion-Ordered NMR Spectra: Can We Rationalize Small Molecule Diffusion Coefficients? *Angew. Chem. Int. Ed. Engl.* **2013**, *52* (11), 3199–3202. https://doi.org/10.1002/anie.201207403.
